# Cellular aspects of Na^**+**^ homeostasis in plants: Quantitative approaches

**DOI:** 10.1017/qpb.2026.10040

**Published:** 2026-03-02

**Authors:** Stephen Tyerman, Tracey Ann Cuin, Jayakumar Bose, Rana Munns

**Affiliations:** 1 School of Agriculture Food and Wine, Adelaide University, Australia; 2 School of Science, Western Sydney University, Australia; 3 The University of Western Australia – Perth Campus, Australia

**Keywords:** organelle sodium, salinity, sodium homeostasis, sodium potassium interaction, sodium transport

## Abstract

Under saline conditions, plants consistently maintain cytosolic Na^+^ concentrations between 10 and 30 mM, sequestering excess Na^+^ to the vacuole. We demonstrate that this cytosolic Na^+^ homeostasis is regulated by inward Na^+^-permeable channels and outward Na^+^:H^+^ antiporters at both the plasma membrane and tonoplast. Sodium’s interplay with K^+^ transport adds complexity and selective transport is crucial to avoid conflicting ion fluxes. Our models predict that Na^+^:H^+^ antiport regulation at the plasma membrane significantly impacts cytosolic Na^+^ levels, while channel and antiport regulation are equally important at the tonoplast. The energetic implications of these transport mechanisms are discussed. In contrast to the cytosol, chloroplast Na^+^ concentrations vary significantly between species and increase with soil salinity, raising questions as to how C_4_ and CAM plants acquire pyruvate under saline conditions. However, modelling transport activity at the chloroplast membrane requires far more knowledge of the associated transport systems and the chloroplastic Na^+^ content.

## Graphical abstract

**Figure:**
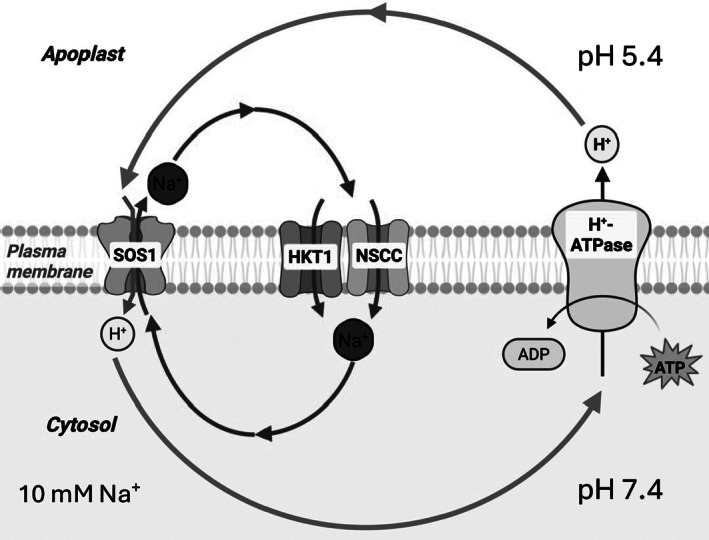

## 1. Introduction

Homeostasis is a self-regulating process in which biological systems maintain stability while the organism adjusts to changing external conditions. When the system is disturbed, regulatory processes respond to any changes to restore the original internal status or to establish a new balance between external and internal conditions. Homeostasis is not static and unvarying; it is a dynamic process *requiring energy* that can change internal conditions to survive external challenges (Billman, 2020). This concept explains how an organism can maintain its internal conditions, allowing it to adapt to and survive a variable, often hostile external environment.

The sodium ion (Na^+^) is used by all plants for generating sufficient osmotic pressure to maintain cell turgor and the volume of intracellular organelles. Because it is ubiquitous in soil and the atmosphere, plants rarely encounter Na^+^ deficiency. However, certain C_4_ and CAM species require Na^+^ for transporting pyruvate into mesophyll chloroplasts, the first step of photosynthesis, so for them, Na^+^ is an essential nutrient (Subbarao et al., 2003). Halophytes, the native flora of saline soils, need NaCl for optimal growth, and some species grow best at 100–200 mM NaCl. Nonetheless, even these plant species will suffer Na^+^ toxicity if the soil NaCl concentration exceeds a certain level, a level that varies widely with species. However, the concentration at which Na^+^ becomes toxic in the tissues of any plant or within various cell types and subcellular compartments is still largely unknown and Na^+^ homeostasis is a complex concept that can be considered at many levels in a plant.

In this article, we summarise the known data for Na^+^ concentrations in the various cell compartments. Focusing further on the cellular level, we investigate how Na^+^ fluxes might be regulated by transporters at the plasma membrane (PM) and tonoplast (TP) to maintain low cytosolic concentrations of this toxic ion whatever the external NaCl concentration. We present a quantitative model that illustrates the control of Na^+^ concentrations in the cytosol and vacuole by known Na^+^ transporters.

## 2. Na^**+**^ **concentrations in tissues and cells during changes in soil salinity**

For whole plant physiologists, Na^+^ homeostasis is considered as the steady-state tissue concentration that remains constant despite a continual influx of Na^+^ from a saline soil. This concentration differs between species even at the same level of soil salinity. Generally, the more salt tolerant the species, the higher the maintained leaf [Na^+^] (Munns et al., 2016). For example, barley (a salt-tolerant species) maintains a higher concentration than wheat (salt sensitive) at the same external NaCl supply (James et al., 2006) and the concentration in leaves of halophytes is even higher (Flowers et al., 2015).

### 2.1. ***Tissue-level Na***^***+***^ ***concentrations***

**Figure 1.**
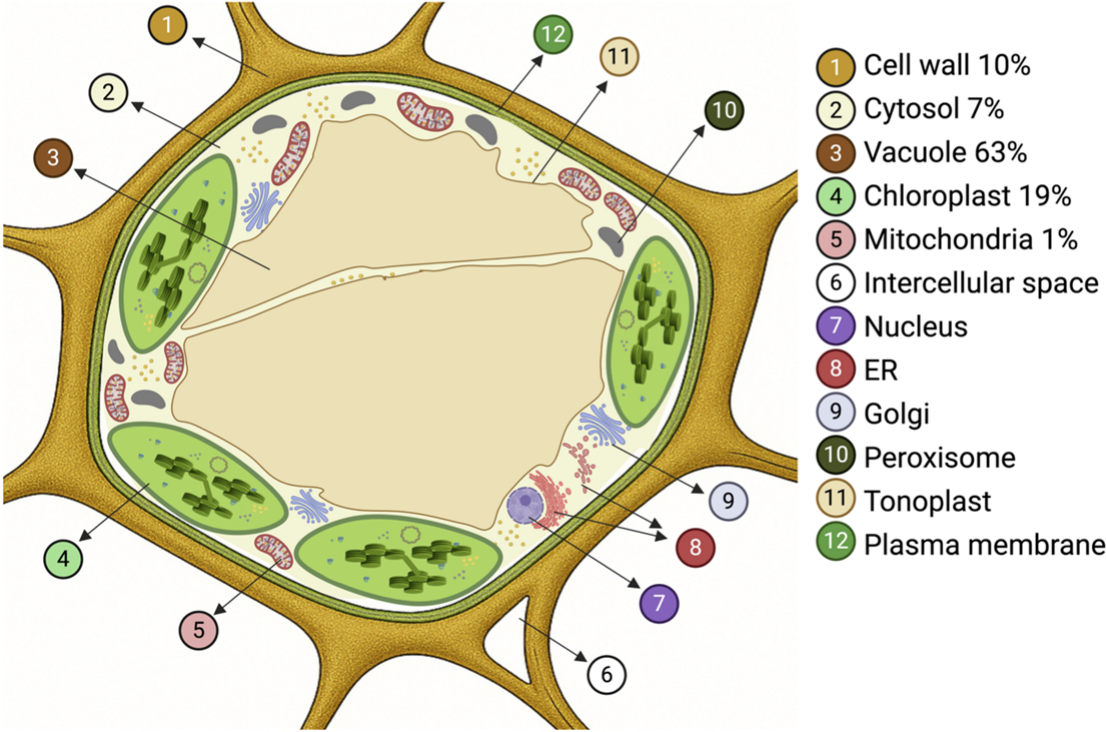
The subcellular compartments of a leaf mesophyll cell and the percent of the total cell water occupied by the wall, the cytosol and some organelles. Also shown is the plasma membrane, tonoplast and a cytoplasmic strand crossing the vacuole.

At the tissue level, the status of homeostasis is easily measured by following the [Na^+^] for individual leaves over time. For example, in durum wheat exposed to 150 mM NaCl, the [Na^+^] remained about 150 mM (tissue water basis) in a newly emerged leaf for an entire month. In contrast, in bread wheat, it remained at only 10 mM over the same time period (Rivelli et al., 2002). This very low leaf Na^+^ concentration is due to the action of the HKT1;5-D transporter located on the D genome of bread wheat, which retrieves Na^+^ from the xylem in the roots and reduces the transport of Na^+^ from roots to leaves (Munns et al., 2020a). A steady-state or plateau that is maintained in a given leaf for a period of days to months is normal for all species as long as the salt treatment is low enough to allow growth to proceed. In a very salt-sensitive cultivar of rice, tissue [Na^+^] in individual leaves of plants grown in 50 mM NaCl increased over time, that is, plants could not maintain homeostasis in this salt treatment (Yeo & Flowers, 1986). However, there is considerable variation between rice species and cultivars in their ability to limit Na^+^ accumulation in leaves, which is related to leaf sheath accumulation of Na^+^ (Goto et al., 2022) and the role of HKT1;5-mediated Na^+^ transport in roots (Shohan et al., 2019). In addition, the measured concentration of Na^+^ in leaves is essentially the concentration in the cell vacuoles ([Na^+^]_vac_) because this compartment contains about two-thirds of the tissue water and occupies at least 70% of the cell volume (Figure 1).

### 2.2. ***Na***^***+***^ ***concentrations in the cell wall***

In leaves, the cell wall holds a significant proportion of tissue water, which can vary from 3% in succulents to 40% in woody species (reviewed by Cutler et al., 1977). The concentration of Na^+^ in leaf cell walls is difficult to measure, but is likely to be similar to, or possibly lower in the leaf apoplast than in the incoming xylem sap (Grignon & Sentenac, 1991): shuttling of Na^+^ from the cell to the apoplast and *vice versa*, as predicted for K^+^ (Dreyer, 2021), will modify the [Na^+^]_wall_. Indeed, values as low as 6 mM are reported from maize leaf cells grown in 100 mM NaCl (Shahzad et al., 2019), with 15 and 30 mM measured in cotton plants subjected to 75 and 150 mM NaCl, respectively (Mühling & Läuchli, 2002) and concentrations of 6 mM in spinach grown in 100 mM (Speer & Kaiser, 1991).

These values are consistent with published data for [Na^+^] in the xylem sap (reviewed by Munns et al., 2020b), demonstrating that plants exclude around 95% of acquired Na^+^ back to the soil solution. Hence, so plants growing in soils containing 100 mM would have around 5 mM Na^+^ in the xylem flowing to leaves.

In roots, the cell wall Na^+^ concentration ([Na^+^]_wall_) is unknown although it is predicted to be similar to the soil solution in root epidermal cells. We take the external Na^+^ concentration ([Na^+^]_ext_) used in the models to be that of the concentration of NaCl supplied to the plant.

### 2.3. ***Cytosolic Na***^***+***^ ***concentrations***

The cytoplasm, the gel-like matrix between the PM and the TP, is about 30% of the protoplasmic volume of a typical leaf mesophyll cell. The cytosol is the protein-rich fluid that envelops all the membrane-bound organelles: the two energy-producing organelles, the mitochondria and chloroplasts, as well as the nucleus, the endoplasmic reticulum and peroxisomes, which all appear to have their own specific homeostatic [Na^+^].

Measurements of the cytosolic Na^+^ concentration ([Na^+^]_cyt_) are scarce due to technical difficulties in measuring concentrations in such a small volume: the cytosol is just a thin layer of 1–2 μm around the organelles. Nonetheless, the [Na^+^]_cyt_ can be expected to be below 100 mM because *in vitro* studies demonstrate that Na^+^ inhibits most cytosolic enzymes as concentrations approach this level (Flowers et al., 2015; Greenway & Osmond, 1972). The only *in vivo* measurements are on barley root cortical cells from plants grown in 200 mM NaCl where activities of 2–28 mM [Na^+^]_cyt_ are recorded alongside a K^+^ activity of 40–60 mM (Carden et al., 2003). More information is available for animals where the [Na^+^]_cyt_ of heart muscle cells is reportedly less than 10 mM (Bers et al., 2003; Hernansanz-Agustín et al., 2024). Another approach has been to measure tissues with a high proportion of cell volume as cytoplasm, that is, cells with small vacuoles such as in plant meristems. These results, together with those of Carden et al. (2003), indicate that the [Na^+^]_cyt_ falls below 10 mM for plants grown without added NaCl and up to 30 mM when grown in solutions containing 100–200 mM NaCl (Table 1). Other methods, including X-ray microanalysis and the fluorescent dye SBF1 on root, leaf and grain tissues, report a similar range of results (Table 1; Supplementary Table S1). Similar data have been obtained in cells of the green micro-alga *Chlorella emersonii* with fully developed chloroplasts. When grown at 1, 100 or 335 mM NaCl, the cells contained only 9, 18 and 21 mM Na^+^, respectively (Greenway & Setter, 1979, Supplementary Table S1).

**Table 1:** Estimates of Na^+^ in the cytosol. Data are for measurement of cytosol or for tissues containing cells with small vacuoles and no chloroplasts

Species	Tissue	[Na^+^]_cyt_ (mM)		References
		Low [Na^+^]_ext_	High [Na^+^]_ext_	
Barley (2 cvs)	Cortical cells	-	19	Carden et al., 2003
Arabidopsis	Root hairs	1	15	Halperin & Lynch, 2003
Rice (2 cvs)	Root & shoot protoplasts	2	15–25^*^	Kader & Lindberg, 2005
Rice (2 cvs)	Grain cell culture	2	25–80^*^	Anil et al., 2007
Atriplex	Root tip	5	30	Jeschke et al., 1986
Cotton	Root tip	10	25	Zhong & Läuchli, Zhong & Laüchli, 1994
Barley (2 cvs)	Shoot apex	6	20	Munns & Rawson, 1999
Bread wheat	Shoot apex	2	9	Munns & Rawson, 1999
Lettuce	Shoot apex	15	25	Lazof & Läuchli, Lazof & Laüchli, 1991
Arabidopsis	Mesophyll protoplasts	2	7	Morgan et al., 2022

*Note: Details of measurement methods, NaCl treatments, and Na^+^, K^+^ and Cl^−^ concentrations are given in* Supplementary Table S1.

* Salt-sensitive cultivars.

The limited variation in the [Na^+^]_cyt_ for plants grown in low or high concentrations of NaCl demonstrates that in terms of homeostasis, there is a permissible range within which the [Na^+^]_cyt_ is maintained for cellular function. The control of [Na^+^]_cyt_ might also be a component of a broader homeostasis such as the total cation concentration in the cytosol in conjunction with [K^+^] or osmotic regulation. Clearly though, [Na^+^]_cyt_ must be kept below a certain limit.

### 2.4. ***Mitochondrial Na***^***+***^

The Na^+^ content of plant mitochondria has not been measured. In animal cells it ranges from 5 to 50 mM (Donoso et al., 1992; Hernansanz-Agustín et al., 2024; Jung et al., 1992; Murphy & Eisner, 2009; Pike et al., 1990), two to eight times lower than the cytosol (Donoso et al., 1992; Jung et al., 1992). This concentration gradient, together with an inward directed membrane potential (V_m_) in the range −150 to −180 mV and a more alkaline matrix (Boyman et al., 2013), means there is a large driving force for Na^+^ entry. This is utilised by the mitochondrial Na^+^/Ca^2+^ exchanger (NCLX) that powers Ca^2+^ efflux from the mitochondrial matrix (Boyman et al., 2013; Palty & Sekler, 2012), likely counter-balanced by the activity of a mitochondrial Na^+^/H^+^ exchanger (NHE) that exploits the trans-inner mitochondrial H^+^ gradient generated by the respiratory chain (reviewed in Nita et al., 2015). However, it is considered unlikely that mitochondria have any major role in regulating [Na^+^]_cyt_ under basal conditions in animals (Murphy & Eisner, 2009). Whether this is different in salinity-affected plants is unknown. Nonetheless, if mitochondria in mammals and plants emanate from the same common ancestral organelle that originated from the integration of an endosymbiotic alphaproteobacterium into an Archaea cell (Roger et al., 2017), it might be expected that the mitochondrial [Na^+^] is similar and similarly regulated. However, this information is still not available.

### 2.5. ***Na***^***+***^ ***concentrations in chloroplasts***

Better studied in plants are the chloroplasts, the main component of the cytoplasm (Bowsher & Tobin, 2001) although measurements of their [Na^+^] are sparce, particularly for non-halophytes. The few reports do indicate that, in contrast to the cytosol, chloroplastic [Na^+^] varies widely between species (Table 2) and can be high in halophytes (Flowers et al., 2015) (Table 2). Nonetheless, concentrations are still far lower (about sixfold) than the leaf concentration (Supplementary Table S2). For example, Robinson and Downton (1985) recorded a concentration of about 100 mM in *Suaeda maritima* chloroplasts under both low and high salinity (350 mM NaCl) even though the leaf [Na^+^] increased from 10 mM to 650 mM and Cosentino et al. (2010) found that in the six days after supplying 400 mM NaCl, the chloroplastic Na^+^ of *Mesembryanthemum crystallinum* plateaued at about 100 mM then remained at this level, a condition termed ‘homeostatic’.

**Table 2:** Measurements of Na^+^ K^+^ concentrations (mM) in chloroplasts from plants grown in low and high NaCl

Species	No added external NaCl			High external NaCl				References
	Na^+^	K^+^	Na^+^+K^+^	NaCl_ext_	Na^+^	K^+^	Na^+^+K^+^	
Glycophyte								
Mungbean cv Jade	10	120	130	75	11	150	161	Iqbal et al. (2024)
Halophyte related								
Sugarbeet	71	167	238	–	–	–	–	Robinson and Downton (1984)
Spinach	81	195	276	200	138	109	247	Robinson and Downton (1984)
Spinach	96	210	306	200	165	121	286	Robinson et al. (1983)
Spinach	7	180	133	300	22	108	130	Schroppel-Meier and Kaiser (1988)
Spinach	7	126	133	200	32	101	133	Kaiser et al. (1983)
				350	71	156	227	Kaiser et al. (1983)
Glycophyte average	45	166	203		73	124	197	
Halophytes								
*Chloris gayana* (Rhodes grass, C_4_):								
▪ mesophyll	29	200	229	200	87	162	249	Oi et al. (2022)
▪ bundle sheath	10	116	126	200	34	139	173	Oi et al. (2022)
*Suaeda australis*	106	232	328	350	115	73	258	Robinson and Downton, (1985)
*Suaeda maritima*	–	–		340	84	22	106	Harvey et al. (1981)
*Suaeda maritima*	28	43	71	340	84	23	107	Hajibagheri et al. (1984)
*M. crystallinum*	2	133	135	20	124	97	211	Demmig and Winter (1986)
*M. crystallinum*				400	195	45	240	Demmig and Winter (1986)
*M. crystallinum*	15			400	110			Cosentino et al., 2010
Halophyte average	32	145	178		104	80	192	

*Note: Data are from aqueous extraction of chloroplasts or in situ SEM X-ray microanalysis (for further details see* Supplementary Table 2 *). Mesembryanthemum crystallinum is a facultative CAM species.*

In contrast, in glycophytic plants such as mungbean (Iqbal et al., 2024), concentrations reach only 30 mM in high NaCl, which is comparable to that measured for the cytosol in most species (Table 1). Similarly, low levels of Na^+^ have been found for other glycophytes (Supplementary Table S2). Much of the variation between species or between different publication for plants grown without added NaCl is probably due to low levels of Na^+^ occurring in soils or tap water. That the variation between experiments is real is supported by data for K^+^ and Cl^−^. In most cases when Na^+^ increases, K^+^ decreases (Table 2). Of interest in the sense of possible homeostasis of cations in the chloroplast is that the sum of Na^+^ plus K^+^ is similar in all species and increases very little in plants grown in low salinity compared to those subjected to high salinity – from 191 to 210 mM (Table 2).

### 2.6. ***Chloroplast transporters that regulate Na***^***+***^ ***influx and efflux***

With a lower or equal [Na^+^] in the chloroplast compared to the cytosol, a higher pH (Höhner et al., 2016; Sekiguchi et al., 2024) and a negative trans-envelope voltage difference with respect to cytosol (Demmig & Gimmler, 1983; Igamberdiev & Kleczkowski, 2003), Na^+^ is likely to enter the chloroplast stroma passively and require active transport for export. The transporter families that might contribute to Na^+^ import across the inner envelope membrane are the Phosphate Transporters (PHT), the Bile Acid/Sodium Symporters (BASS) and Non-Selective Cation Channels (NSCC), with Na^+^/H^+^ Antiporter (NHAD)-type carriers and a Cation/H^+^ eXchanger (CHX) family member possibly involved in chloroplastic Na^+^ export, as illustrated in Supplementary Figure S1.

Inorganic phosphate (Pi) is essential for ATP synthesis during the light reactions of photosynthesis, and PHT4 family members contribute towards its import. The activity of these transporters is dependent on co-transport with a cation, but whether this cation be H^+^ or Na^+^ is uncertain. For instance, Arabidopsis AtPHT4;1 shows Na^+^-dependent Pi transport activity when expressed in *E. coli* (Pavón et al., 2008) but H^+^-dependent activity when expressed in yeast (Guo et al., 2008). Likewise, Na^+^ has been shown to be the co-transported substrate for AtPHT4;4 when expressed in *Xenopus* oocytes and yeast in one study (Finazzi et al., 2015), but dependent on H^+^ when using a different approach when again expressed in yeast (Guo et al., 2008). Unfortunately, the co-transported cation *in planta* is still not reported nor is the transporter’s contribution towards the chloroplastic [Na^+^].

Also potentially contributing to Na^+^ uptake into the chloroplast is the Na^+^-dependent pyruvate (Pyr) transporter BASS2 (Furumoto et al., 2011), the major mediator of Pyr import into chloroplasts. This transporter is far more abundant in halophytes than in glycophytes and C_4_ plants. However, the reported high [Na^+^] in halophyte chloroplasts is perplexing: this would likely prohibit the BASS2-mediated import of Na^+^ (so also Pyr), assuming a lower [Na^+^]_cyt_. In addition, Pyr must enter mesophyll chloroplasts for regeneration of PEP as the acceptor for CO_2_ by PEP carboxylase in C_4_ photosynthesis. Consequently, we might expect the [Na^+^] in chloroplasts to be lower in mesophyll cells of C_4_ plants than in the bundle sheath cells, assuming equal [Na^+^]_cyt_. Conversely, the reverse was found in the C_4_ halophyte Rhodes grass: Na^+^ was three times higher in mesophyll chloroplasts than in bundle sheath chloroplasts (Table 2). In *Mesembryanthemum crystallinum* in CAM mode under high salinity, measurements indicate potentially high [Na^+^] in chloroplasts (Demmig & Winter, 1983), which corresponds with other data (Table 2). Under these conditions, the pH gradient across the chloroplast membrane is increased, favouring inward H^+^ movement. Some plant species have been shown to have a H^+^/OH^−^-coupled pyruvate uptake mechanism (Aoki et al., 1992) and given the unfavourable Na^+^ gradient under saline conditions, Pyr is likely taken up by a H^+^-dependent co-transporter the molecular identity of which is still awaiting discovery.

Orthologues of BASS2 have been detected in the genomes of all plants analysed so far. Arabidopsis has six BASS members, AtBASS1 to AtBASS6, but their role is not well characterised. And although Zhao et al. (2016) reported that overexpression of TaBASS2 could enhance the salt tolerance of wheat and Arabidopsis, they did not show that this was due to increased transport of Na^+^ by this transporter. Instead, TaBASS2’s overexpression appeared to inhibit the expression of ABI4 (ABA Insensitive 4), a transcription factor that links ABA (Abscisic Acid) signalling and plastid retrograde signalling pathways to the salinity response.

A significant contribution to Na^+^ influx can occur via the inner envelope membrane-located Fast Activated Cation Channel (FACC; Pottosin & Shabala, 2005). This channel is equally permeable to K^+^ and Na^+^, and it has been shown to transport Na^+^ across the chloroplast membrane. Also, two members of the Arabidopsis MechanoSensitive channel Like (MSL) family, MSL2 and MSL3, localise to the chloroplast inner envelope (Haswell & Meyerowitz, 2006), although their ion selectivity and ability to transport Na^+^ are still unknown (Pottosin & Dobrovinskaya, 2015).

The export of Na^+^ from chloroplasts has been attributed to the activity of the inner envelope-localised NHAD-type carriers. This has been clearly demonstrated for the Arabidopsis AtNHD1 (Müller et al., 2014), which transports Na^+^ against its electrochemical gradient. AtNHD1 expression in *E. coli* decreased the bacteria’s [Na^+^] and under saline conditions, Arabidopsis mutants lacking this Na^+^/H^+^ antiporter contained more Na^+^ in the chloroplast and had an 88% lower chlorophyll content compared to the wildtype, as well as showing greater NaCl sensitivity.

Interestingly, the Arabidopsis AtNHD1 gene is not induced by salinity, whereas its rice homolog OsNHAD, which localises to the chloroplast, is upregulated under salt stress. Although OsNHAD contributes to salinity tolerance (Liu et al., 2020), it is unclear whether this benefit arises from promoting Na^+^ export from the chloroplast. In RNAi knockdown plants supplied with NaCl, chloroplastic and whole-leaf Na^+^ increased, and the plants were injured or died. Expression of the halophyte *M. crystallinum* homologue McNhaD also increases under saline conditions. However, in contrast to rice and Arabidopsis, McNhaD is proposed to operate in the opposite direction – importing Na^+^ into the chloroplast stroma in exchange for H^+^ (Cosentino et al., 2010). Whether this ‘reverse action’ is to accumulate Na^+^ in the chloroplast in exchange for H^+^ can be questioned: the chloroplast is more alkaline than the cytosol (Höhner et al., 2016; Sekiguchi et al., 2024). Instead, the plant might instead be utilising the lower or at least equal chloroplastic [Na^+^] (Flowers et al., 2015; Flowers & Colmer, 2008) to maintain its trans-envelope H^+^ gradient.

Finally, AtCHX23 might be involved in Na^+^ export from Arabidopsis chloroplasts: it localises to the chloroplast envelope and *Atchx23* mutants display a high sensitivity to NaCl (Song et al., 2004). However, evidence is again circumstantial – poorer performance of knockout plants under saline conditions but no functional characterisation of the transporter to demonstrate its mediation of Na^+^.

The following section develops models for the regulation of Na^+^ in the cytosol and vacuole. Three transporter types on the PM and the TP are essential: a selective Na^+^ transporter that allows passive uptake, a Na^+^/H^+^ antiporter that promotes Na^+^ efflux and a H^+^- ATPase that likely energises the antiporter. It is tempting to speculate that a similar system could apply to the regulation of [Na^+^] in chloroplasts.

## 3. **Membrane transport of Na**^**+**^ **and regulation of cytosol and vacuole Na**^**+**^ **concentrations**

The range of [Na^+^] found in cytoplasmic compartments and at the tissue level discussed above indicates various homeostatic mechanisms that must be contingent upon the interplay of transporters at both the PM and TP to regulate cytosolic sodium ([Na^+^]_cyt_) and vacuolar ([Na^+^]_vac_) concentrations. Although many key transporters have been identified (Supplementary Table 3 and see Assaha et al., 2017), others such as certain ion-conducting aquaporins (Tyerman et al., 2021) and NSCCs (Kronzucker & Britto, 2011) are only known through biophysical evidence and remain undefined at the molecular level. Understanding how these transporters and the energising H^+^ pumps influence Na^+^ distribution and V_m_ across the PM and TP is crucial, particularly given the small volume of the cytosol (Figure 1).

Mathematical models have successfully described changes in cytosolic and vacuolar ion concentrations under salinity stress, incorporating known or approximated kinetic properties for various transporters at both the PM and TP (Foster & Miklavcic, 2015, 2019, 2020). These models reveal emergent behaviours in transient and steady-state responses that align with measured [Na^+^]_cyt_ in rice cells and protoplasts from various plant species and varieties differing in their salinity tolerance (Anil et al., 2007; Foster & Miklavcic, 2015; Kader & Lindberg, 2005) (Table 1). This highlights the role of dynamic transporter kinetics rather than a classic homeostatic feedback system of Na^+^ homeostasis.

This section adopts a steady-state approach to analysing Na^+^ homeostasis and builds on previous models for K^+^ homeostasis (Dreyer, 2021; Dreyer et al., 2024) where the relevant equations are well described. The method avoids the need for detailed kinetic properties of the associated transporters since at steady-state, net flux is zero and concentrations remain constant. The system is energised by H^+^ pumps at both the TP and PM and knowledge of their response to V_m_ is key to understanding permissible steady-states.

### 3.1. **A plasma membrane Na**^**+**^ **homeostat**

Dreyer (2021) describes a PM ‘homeostat’ where transporters achieve zero net flux and maintain constant membrane concentration gradients. This does not mean constant [Na^+^]_cyt_ or [Na^+^]_vac_ but instead implies that the homeostat adjusts to new steady gradients in response to external [Na^+^] changes. Achieving flux balance requires at least two differently energised ion transporter types, which incurs an energetic cost from H^+^ pumping for the so-called futile cycles (Britto & Kronzucker, 2006; Munns et al., 2020a; Shabala et al., 2020) that enable homeostatic flexibility (Dreyer, 2021).

**Figure 2.**
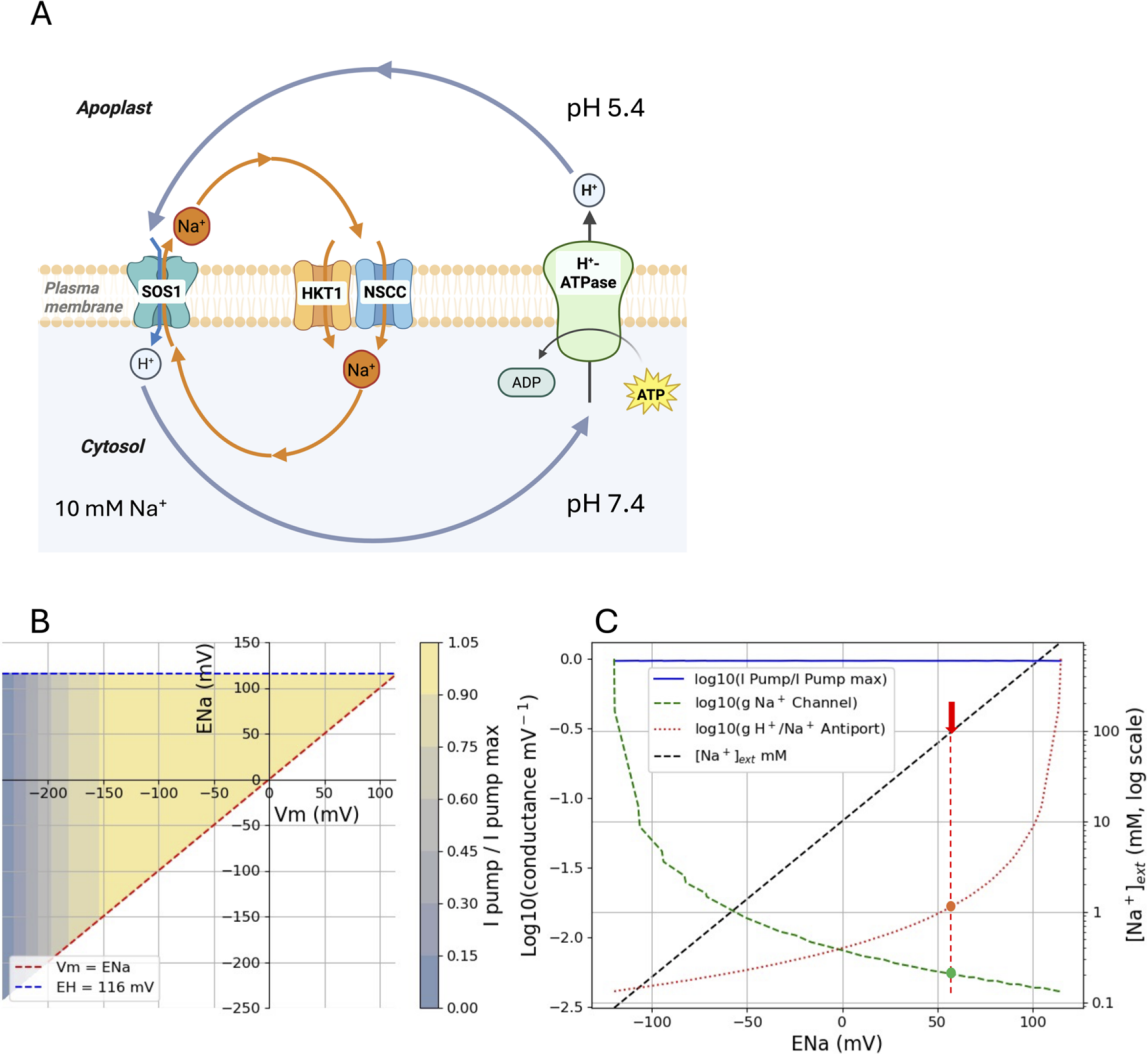
Basic Na^+^ ‘homeostat’ after Dreyer (2021) and Dreyer et al. (2024) for the steady-state cycles of Na^+^ and H^+^ unidirectional fluxes across the PM. The scheme in (A) is translated mathematically by setting a range of conductances (*g*) of the SOS1 antiport and HKT1 where present, or NSCC uniport (channel) (Supplementary Material 2.1, Figure S2). (B) The homeostat shown in (A) has limits irrespective of the activities of the transporters (*g* values) such that E_Na_ cannot be less than V_m_ or greater than the equilibrium potential for H^+^ set here as 116 mV (2 pH unit difference across the PM). The region between these limits is shaded according to the relative pump current. A particular combination of V_m_ and E_Na_ can exist anywhere within the shaded area and depends on the activities of the transporters (values of *g*). The pump has a reversal potential near −240 mV, setting the minimum limit for V_m_. The pump current becomes maximal at less negative V_m_ and is defined as given by Tyerman et al. (2001) for wheat root cortical cell protoplasts (Supplementary Material 2.1, Figure S3). (C) There is a range of *g* values within the constraints of V_m_, E_Na_ and E_H_. Setting [Na^+^]_cyt_ to 10 mM and V_m_ to −120 mV, the changes in *g* for SOS1 (antiport) and HKT1/NSCC (channel) (left *y*-axis, log scale) can be plotted against E_Na_ (*x*-axis). The dashed diagonal line plots [Na^+^]_ext_ (right axis) against E_Na_ (*x*-axis) for a constant [Na^+^]_cyt_ = 10 mM, thus at E_Na_ = 0 mV the external concentration is 10 mM. The red arrow indicates where [Na^+^]_ext_ = 100 mM and the *g* for the Na^+^ antiport (red) and channel (green) that would achieve a [Na^+^]_cyt_ of 10 mM.

Figure 2A illustrates the basic cycle at the PM. This involves a H^+^-ATPase that pumps H^+^ out of the cell, a Na^+^ channel or uniport (NSCCs and Na^+^-specific HKTs which may be present in more cells than the phloem, Supplementary Table S3) and a Na^+^:H^+^ antiport (e.g. SOS1, Zhu, 2000). Equations 35 and 36 from Dreyer et al. (2024) can be solved using linear algebra and some implemented functions available in Python for solving roots of equations. The Python coding and plotting used for the solutions are shown in Supplementary Material 2.1, and the code was optimised and debugged using generative AI (Gemini 2.5 Pro, Claude 4, GTP-5.1). For the basic Na^+^ homeostat shown in Figure 2, the steady-state V_m_ was found by solving a current-balance equation (net Na^+^ and H^+^ flux = 0) for each pair of Na^+^ channel and H^+^/Na^+^ antiporter conductances (*g*) (over a range of values) at a fixed proton equilibrium potential (E_H_). The pump current depends nonlinearly on V_m_, and once V_m_ is found, the corresponding Na^+^ equilibrium potential (E_Na_) is computed from it.

A key finding is that E_Na_ cannot be more negative than V_m_ or more positive than E_H_ (Figure 2B). The E_H_ is fixed in the simulation (blue-dashed line) and is set equivalent to a 2 pH unit difference across the PM (pH 7.4 cytosol and pH 5.4 apoplast), which generates an E_H_ voltage of approximately 116 mV, as calculated from the Nernst equation. The negative V_m_ limit is set by the reversal potential of the H^+^-ATPase, in this case, about −240 mV (Supplementary Material 2.1, Figure S3).

The Na^+^ ‘homeostat’, as defined by Dreyer (2021), sets the limits for what can be achieved for [Na^+^] gradients across the PM, so can be considered a part of the homeostatic control of [Na^+^]_cyt_ where the limits are set by the steady-state balance of fluxes via the ion ‘homeostat’. This is best illustrated in Figure 2C where the model solutions for the system are applied to a situation where the [Na^+^]_cyt_ is set at 10 mM (i.e. perfect homeostasis, see Table 1) with the PM V_m_ set at a typical plant cell V_m_ of −120 mV (e.g. for barley root cells, Bose et al., 2014). The conductances of the channel and antiporter are plotted on the left *y*-axis in Figure 2C as a log scale against the E_Na_ (*x*-axis), while the black diagonal dashed line indicates the [Na^+^]_ext_ (right *y*-axis) as a function of E_Na_. Note that this shows conductances as a function of E_Na_ for V_m_ = −120 mV, which is only one of the many solutions indicated by the shaded section in Figure 2B. Thus, at an E_Na_ of 0 mV, [Na^+^]_ext_ is 10 mM and the conductances via the channel and antiport are about equal. To adjust to a higher [Na^+^]_ext_ at steady-state, say 100 mM (marked with an arrow), and to keep [Na^+^]_cyt_ at 10 mM, the Na^+^:H^+^ antiport conductance has to increase (note the log scale) and the Na^+^ channel conductance has to decrease to achieve flux balance. It is important to realise that both the antiporter and channel must change their respective conductances for this new steady-state to occur, so both need to be regulated by various measures. This could include changes in the phosphorylation status of SOS1 (Qiu et al., 2002) and PIP2;1 as a candidate for an NSCC (Qiu et al., 2025), and by reciprocal regulation of SOS1 and HKT1 (Gámez-Arjona et al., 2024). There is clearly a limit where the antiport flux of H^+^ approaches that of the pump.

What happens if there is no adjustment in conductances when [Na^+^]_ext_ is increased from 10 mM to 100 mM? In this situation, the E_Na_ would remain constant and for this to occur there would be an increase in [Na^+^]_cyt_ at a constant V_m_. For example, if E_Na_ was 58 mV (red arrow in Figure 2C, approximately a 10-fold concentration gradient from the Nernst equation), the increase in [Na^+^]_ext_ from 10 to 100 mM would require a 10-fold increase in [Na^+^]_cyt_, say from 1 to 10 mM. This is not really Na^+^ homeostasis but may occur in some salt-sensitive species (see below).

### 3.2. ***K***^***+***^ ***and Na***^***+***^ ***interact***

Potassium homeostasis is intrinsically linked to salinity tolerance (Cuin et al., 2008; Shabala & Cuin, 2008) and Na^+^ homeostasis (Assaha et al., 2017). Modelling K^+^ and Na^+^ transport together using H^+^-ATPase and various channels and symporters (e.g. AKT or GORK for K^+^ channels, HAK or KUP for K^+^:H^+^ symport, Supplementary Table S3) allows for flux balance solutions (Supplementary Material 2.2, Figure S4). While the direct interaction of K^+^ and Na^+^ via the same transport system (e.g. NSCCs) is complex and not accounted for, our analysis sets boundaries for K^+^ and Na^+^ homeostasis. The transport of Na^+^ and K^+^ through the same channels can profoundly impact steady-state solutions and for K^+^ and Na^+^ homeostats to co-exist, at least one of the uniports or H^+^-coupled systems must exhibit high selectivity. This is fundamental because it is unlikely that opposite fluxes of Na^+^ and K^+^ can occur through the same transporter or channel. GORK channels, for instance, are highly K^+^:Na^+^ selective (Schachtman et al., 1991). Nonetheless, the extensive signalling that regulates GORK channels under abiotic stress (Adem et al., 2020; van Kleeff et al., 2018) is very relevant to Na^+^ homeostasis: the up- or down-regulation of GORK will indirectly affect the Na^+^ homeostat via the proportion of the H^+^ flux that is available for Na^+^:H^+^ antiport relative to that required for K^+^ homeostasis (Supplementary Figure S5).

The Na^+^ homeostat (as in Figure 2) plus a K^+^ homeostat that comprises a K^+^ channel and K^+^:H^+^ symport is shown in Supplementary Figure S4. From the plots of V_m_ versus E_Na_ and V_m_ versus E_K_, the addition of the K^+^ transport systems decreases the negative limit of V_m_: it becomes more positive. This makes sense with respect to the balance of the H^+^ flux through the H^+^-ATPase, which powers both Na^+^ and K^+^ cycling.

**Figure 3.**
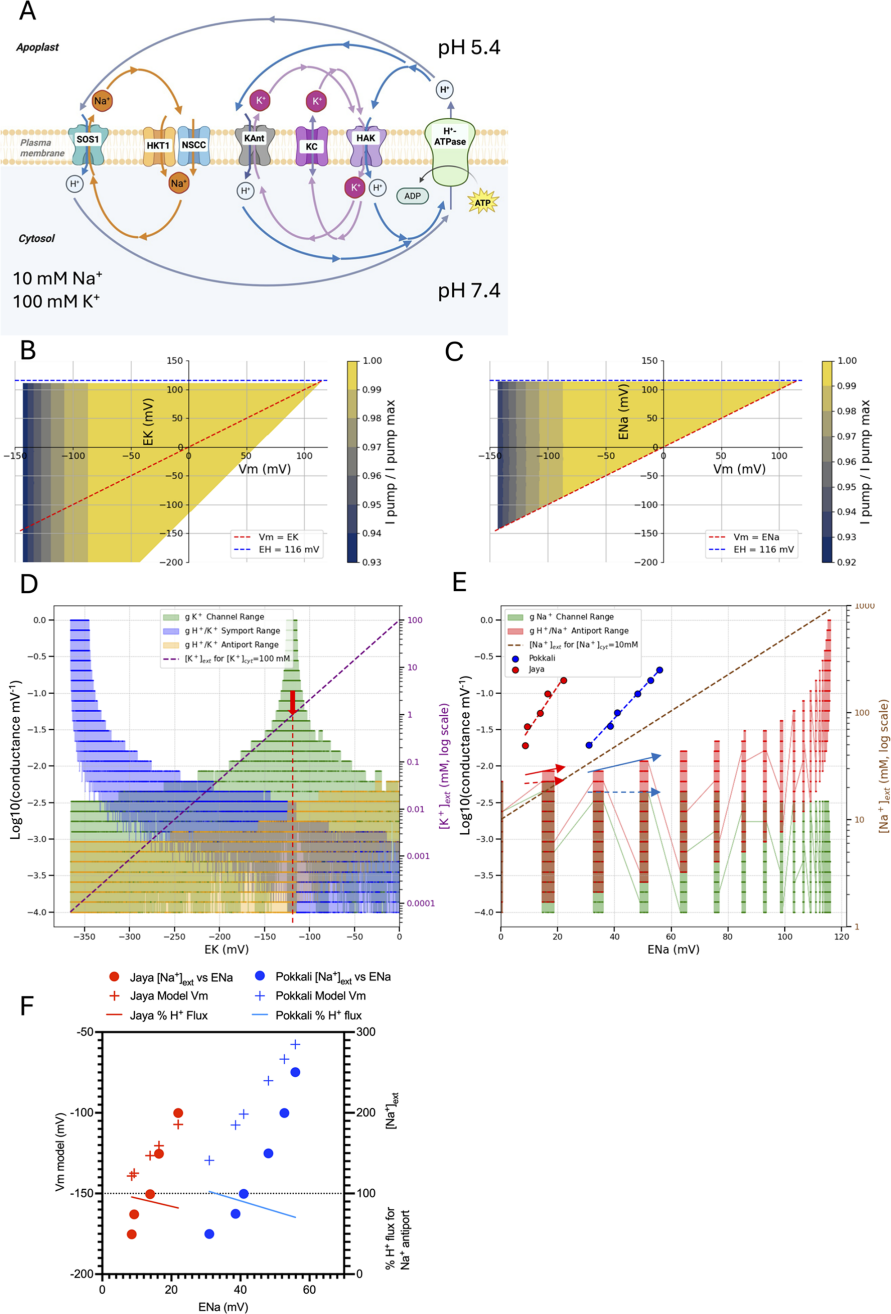
(A) Combined homeostats for K^+^ and Na^+^ where a K^+^:H^+^ antiport has been added in addition to the transporters shown in Supplementary Figure S4. (B) Modelling the steady-state for (A) where the constraints on E_K_ (B) and E_Na_ (C) are shown as a function of V_m_. (D) K^+^ conductances (K^+^ symport, antiport and channel) are plotted against E_K_. The K^+^ channels (AKT1 and GORK) can mediate either inward or outward fluxes, depending on E_K_ relative to V_m_. The constraints are V_m_ = −120 mV, [K^+^]_cyt_ = 100 mM. The red arrow indicates E_K_ for [K^+^]_ext_ = 1 mM. (E) Na^+^ conductances (Na^+^ antiport and Na^+^ channels) as a function of E_Na_ (V_m_ = −120 mV). Perfect cytoplasmic Na^+^ homeostasis is shown by the diagonal dashed line for a constant [Na^+^]_cyt_ of 10 mM. Also plotted are E_Na_ data versus [Na^+^]_ext_ from measurements of [Na^+^]_cyt_ on a cell line from two rice cultivars (Pokkali salt tolerant, Jaya sensitive, Anil et al., 2007). The fitted lines are exponential fits to the data taken from Anil et al. (2007) (their Figure 2C (R^2^ > 0.9)). Also shown is the required change in *g* between steady-states for Na^+^ permeable channels (dashed arrow) and Na^+^:H^+^ antiport (solid arrow) for the two cultivars. Note the range in *g* for any particular E_Na_ and E_K_. This is a consequence of combining the K^+^ and Na^+^ homeostats since different proportions of the circulating H^+^ flux can be allocated to the two homeostats (Supplementary Figure S5). (F) A constant Na^+^ antiport conductance is assumed but where the constraint is relaxed on maintaining a constant V_m_ for the rice cell lines shown in (E). With this constraint, the V_m_ depolarises with increased [Na^+^]_ext_ at the measured E_Na_ values. The proportion of H^+^ flux used for the antiport is shown as a percentage of the total H^+^ flux through the pump. The antiport conductance is higher for Pokkali (0.012 mV^−1^) than for Jaya (0.009 mV^−1^) for the same range of V_m_ but the Na^+^ channel conductance does not change (0.0062 mV^−1^).

When a K^+^:H^+^ antiport is added to the PM (Figure 3A), such as the, albeit disputed (de los Ríos et al., 2021) root pericycle cell-located NRT1.5/NPF7.3 (Li et al., 2017), greater flexibility in external K^+^ concentrations can be accommodated (Li et al., 2024). The changes in conductances as a function of E_K_ and E_Na_ are shown in Figure 3D and 3E, respectively, and derived from the equations and code given in Supplementary Material 2.3. In this situation, [K^+^]_ext_ can go higher than when a K^+^:H^+^ antiport is not present under the set conditions of [K^+^]_cyt_ = 100 mM. Also note that the K^+^ channel conductance becomes dominant near to where the E_K_ is close to the V_m_, that is, around −120 mV, a situation reminiscent of the K^+^-state (Beilby, 1986; Gilliham et al., 2006). This peak in K^+^ channel conductance follows the clamped V_m_ (Supplementary Material 2.5, Figure S6).

Comparing Figure 2C with Figure 3D,E indicates that there can be a range of conductances for any particular E_Na_ and E_K_. This is a consequence of combining the K^+^ and Na^+^ homeostats since different proportions of the circulating H^+^ flux can be allocated to the two homeostats (Supplementary Figure S5). The extent of the conductances for Na^+^ and K^+^ is linked by the balance allocated by the H^+^ flux. At a very negative E_K_, the symporter conductance needs to increase, as would be expected at low [K^+^]_ext_, alongside a decrease in the K^+^ channel conductance. These boundaries are helpful in suggesting various optima for the activity of the different transporters when combined and again highlight that control of only one transport system is likely to be insufficient. Nonetheless, to go from a steady-state at [Na^+^]_ext_ = 100 mM to an external concentration of 400 mM, a greater change is required for the antiport conductance relative to that for the channel even though the Na^+^ flux via both transport systems is equal but opposite, provided that the allocation of cycling H^+^ to the Na^+^ homeostat remains the same. The conductances required for steady-state can be easily translated to real unidirectional fluxes via the relationship of the conductances to the maximum pump current (see Table 3).

**Table 3:** Example of calculated unidirectional currents and fluxes across the PM that satisfies the model shown in Figure 3

				Na^+^ efflux	K^+^ efflux	K^+^ influx			% of pump
				(antiport)	(antiport)	(symport)			flux for Na^+^
	I_pump_	IK^+^_channel_	INa^+^_channel_	current = 0	current = 0	current = 2x	current	current	antiport
Transporter	H^+^-ATPase	GORK	HKT1 or NSCC?	SOS1	?	HAK			
Unidirectional current/flux (pA or equivalent)	19.3	1.04	−16.9	16.9	0.66	−1.70	−20.3	20.3	87.7
		19.3	12.5	−0.37	0.37	3.23	−15.7	−31.8	31.8	1.9

Na^+^ and K^+^ homeostats are set to values of the maximum pump current in a wheat root cortical cell of approx. 20 pA (Tyerman et al., 2001) under the two scenarios of H^+^ flux used for the Na^+^ homeostat. The solutions of the model are shown for when V_m_, set at −123 mV, is slightly positive of E_K_, E_Na_ is 63 mV, and opposite conductances via the K^+^ channel (e.g. GORK) and the Na^+^ uniport (HKT1 or NSCC).

In Figure 3E, the E_Na_ for two rice cell lines calculated using the Nernst equation from measured data for [Na^+^]_cyt_ (Anil et al., 2007) is shown as function of [Na^+^]_ext_. The E_Na_ is more positive in Pokkali compared to Jaya at the same [Na^+^]_ext_ by virtue of Pokkali maintaining lower [Na^+^]_cyt_ for a given [Na^+^]_ext_. The underlying conductances of the Na^+^:H^+^ antiport and Na^+^ channel can be read from the graph as indicated by the arrows, assuming a constant V_m_ of −120 mV. However, it is more likely that V_m_ progressively depolarises as the [Na^+^]_ext_ increases, provided rice behaves like barley rather than pea (Bose et al., 2014). Unfortunately, Anil et al. (2007) did not report V_m_.

SOS1 might be regulated by phosphorylation in an ON or OFF state as opposed to a graded way related to the intensity of a Na^+^ signal. If this is the case, the steady-state model can be used to generate data for a constant conductance via the Na^+^:H^+^ antiport to provide a range of V_m_ values that would be reasonably obtained. These are shown in Figure 3F where the modelled V_m_ is plotted against E_Na_ for both cultivars. In both cases and under a constant antiporter conductance, V_m_ depolarises over a range that could be reasonably expected from the measurements. In this simulation, E_K_ is kept constant near −116 mV and the V_m_ is predicted to depolarise more for Pokkali than for Jaya, a situation reminiscent of that observed when comparing salt-tolerant barley with salt-sensitive pea (Bose et al., 2014). In addition, the antiporter conductance is necessarily higher for Pokkali than for Jaya to obtain this range of V_m_, but the Na^+^ channel conductance is the same. Also shown on the plot is the proportion of the total H^+^ flux used for the Na^+^ antiport, which declines as E_Na_ increases and V_m_ depolarises. The relative proportion of H^+^ flux is high for the Na^+^ antiport because for the K^+^ homeostat, the flux through the K^+^ channel and co-transport systems is low when V_m_ is near to E_K_. As the V_m_ depolarises and becomes more positive of E_K_, the outward K^+^ unidirectional efflux via the K^+^ channel (GORK) increases and the inward current via the H^+^:K^+^ symport (HAK) must increase, which consumes H^+^. Depolarisation may preserve H^+^ energy for Na^+^ transport and for maintaining SOS1 (as the Na^+^:H^+^ antiport) at a constant conductance. However, K^+^ transport will become more critical as the [Na^+^]_ext_ increases, and if the H^+^:K^+^ symport (HAK) is inhibited or turned down to save energy, there will be a net loss of K^+^ from the cell.

The well-known net loss of K^+^ from cells as [Na^+^]_ext_ is increased (Shabala & Cuin, 2008) may be understood in terms of the scheme in Figure 3. This would occur through a loss of steady-state control when either the K^+^ channel is not turned down sufficiently or the K^+^:H^+^ symport activity is insufficiently increased to support a steady-state when there is no net flux of K^+^ out of the cell. High [Na^+^]_ext_ might also inhibit HAK and AKT1 due to a depolarised V_m_ and the lower affinity transport of Na^+^ via these transporters (Rodríguez-Navarro & Rubio, 2006). In addition, there is cross-talk in Na^+^ and K^+^ signalling (Luan et al., 2009) and sensing of [K^+^]_cyt_ (reviewed in Wegner et al., 2025).

### 3.3. ***A tonoplast Na***^***+***^ ***homeostat***

Similar analyses using the ‘homeostat model’ can be applied to the TP to examine vacuolar Na^+^ and K^+^ homeostasis (Supplementary Material 2.6, Figure S7). As discussed in Section 2.1, tissue [Na^+^], which largely reflects vacuolar concentrations, shows a degree of homeostasis although there can be large differences between species.

The basic TP homeostat also includes H^+^ pumps, K^+^ and Na^+^ permeable channels (e.g. TPK1, TPC1, FV, Gobert et al., 2007; Hedrich et al., 2018; Jaślan et al., 2019) and antiporters (e.g. NHX1, NHX2 and SOS1, Ramakrishna et al., 2025; Salazar et al., 2024) (Supplementary Table S3). The TP pumps (V-ATPase and H^+^-PP_i_ase) generate an electrical current as a function of V_m_ (Li et al., 2024), which is used in the homeostat model (Supplementary Figure S8). While TPC1 prevents Na^+^ efflux from the vacuole (Ivashikina & Hedrich, 2005; Jaślan et al., 2019), it does allow Na^+^ transport in the opposite direction. The elusive FV channel may facilitate Na^+^ cycling back to the cytosol for vacuolar Na^+^ homeostasis. NHX1 and NHX2, although more K^+^ permeable, also transport Na^+^ (Barragán et al., 2012) and are vital for salt tolerance (Apse et al., 1999).

Supplementary Figures S7 B and C show that the TP H^+^ pumps lead to a more restricted range of negative V_m_. For a given cytosolic [Na^+^] and [K^+^], vacuolar [Na^+^] and [K^+^] can reach high values. Increasing [Na^+^]_vac_ requires both the Na^+^ channel and antiport conductances to adjust, which contrasts with the PM where antiport adjustment dominates.

## 4. Energetics of homeostasis

The homeostat models reveal potential optima and limits for cycled fluxes (Dreyer et al., 2024). The extent to which ion channel conductances can be reduced to limit cycling is important for energy efficiency, considering the maximum pump current and the need to minimise ATP usage (Shabala et al., 2020). Under high [Na^+^]_ext_, for example, Na^+^ permeable channels need to have very low relative conductances, but because they function a long way from equilibrium, the unidirectional inward fluxes of Na^+^ can still be significant.

Table 3 presents calculated unidirectional currents and fluxes across the PM for two scenarios of pump H^+^ flux (Tyerman et al., 2001). It demonstrates that maintaining Na^+^ homeostasis at steady-state can be achieved with surprisingly few channels. For instance, with a single-channel conductance for the wheat NSCC of 20 pS (Tyerman et al., 1997), a low open probability means that only 20 channels are needed per cell. However, this also implies electrically noisy steady-states. The high selectivity and conductance of wheat K^+^ outward rectifiers (Schachtman et al., 1991) imply that K^+^-selective channels are less critical for energy efficiency although their activity does influence the proportion of H^+^ flux allocated to the K^+^ homeostat.

Another energy-saving mechanism is to ‘turn off’ a homeostat when not required. For instance, Na^+^ homeostasis may be essential when there is high [Na^+^]_ext_, but if V_m_ is near or equal to E_K_, the K^+^ homeostat can be turned down or turned off, saving circulating H^+^.

Here we have considered only two coupled homeostats (K^+^ and Na^+^), although additional homeostatic systems are likely to operate. For example, nitrate, chloride, ammonium and other nutrient ions may require homeostats similar to those described here for K^+^ and Na^+^. This would impose additional energy demands through increased H^+^ cycling. The question then arises: how does the cell determine H^+^ expenditure (i.e. energy cost) for regulating different ions? Even in the relatively simple case of K^+^ and Na^+^, the question of the cell allocation of H^+^ cycling – so energy – between K^+^ and Na^+^ homeostats remains. The fraction of cellular maintenance energy devoted to sustaining ion gradients and allocation to different ions is an open, unanswered question.

## 5. Testing the homeostat model

The homeostat cycles illustrated in Figures 2 and 3 are for situations when the cell is in steady-state, and there are no net fluxes of the ions in question. This rules out using any net flux measurement systems such as the Microelectrode Ion Flux Estimate (MIFE) technique (e.g. Tyerman et al., 2001). The only way to test whether such a homeostat is operating is to measure unidirectional fluxes. This is experimentally challenging but can be done using isotope flux analysis (Britto & Kronzucker, 2006; Kronzucker & Britto, 2011). Patch clamp can also show the unidirectional flux through a single channel, for example, in cell-attached mode with the pipette clamped at the external bath potential, and this can be combined with MIFE to ensure steady-state (e.g. Gilliham et al., 2006). However, this system has not been used in the context of a homeostat system. It may also be possible to deduce the energetics of homeostats by making accurate estimates of ATP usage for H^+^ pumping when the cell is at steady-state. The development of optogenetic tools in plants (Hedrich & Gilliham, 2025) will allow perturbation of homeostats by activation of specific ion channels by light pulses so potentially enabling examination of the energy cost of homeostasis.

## 6. Conclusions

- The range of [Na^+^]_cyt_ demonstrates that in terms of homeostasis, control is not perfect: it can vary sixfold to sevenfold depending on [Na^+^]_ext_. Such a large range is not strictly compatible with the idea of [Na^+^] homeostasis in a strict sense. More likely is a permissible range within which the [Na^+^]_cyt_ is maintained for cellular function (10–30 mM). The control of [Na^+^]_cyt_ might also be a component of a broader homeostasis such as the total cation concentration in the cytosol in conjunction with [K^+^] or osmotic regulation. Clearly though, [Na^+^]_cyt_ must be kept below a certain limit. [Na^+^]_vac_ will be higher, particularly in species that use Na^+^ for turgor generation. Whole-leaf measurements of Na^+^ largely reflect vacuolar concentrations.
- At the cell level, the homeostat concept can be utilised to make predictions as to how Na^+^ and K^+^ transport systems at the PM and TP are regulated to maintain a tolerable range of cytosolic and vacuolar [Na^+^] gradients in salinity-affected plants. The TP and PM have been treated separately here but can be combined with the homeostat model of Dreyer (2021).
- Ion homeostat models for the major organelles could help to resolve the roles of organelles in homeostatic regulation of [Na^+^]_cyt_. In glycophytes, the maintenance of lower Na^+^ in the chloroplast stroma does seem to better match the gradient requirements for metabolite transport. The situation in halophytes where chloroplast [Na^+^] can reach high levels is less clear. In both plant types, the volume fraction of chloroplasts compared to cytosol would indicate that chloroplast Na^+^ homeostasis has a large effect on cytosolic Na^+^ homeostasis, potentially acting as a [Na^+^]_cyt_ buffer. However, there is insufficient knowledge of the Na^+^ transporters and how H^+^ gradients are generated across their bounding membranes to enable a similar attempt at modelling homeostasis.
- Using likely concentration gradients across the TP and PM alongside likely V_m_, the homeostat models that incorporate known and hypothesised transport predict that Na^+^:H^+^ antiport is regulated for homeostatic control at both these membranes, but Na^+^ permeable channels might not be as regulated at the PM as it is at the TP.
- The Na^+^:H^+^ antiport (SOS1) might be regulated in an ON:OFF manner rather than in a graded way to achieve Na^+^ homeostasis over a range of [Na^+^]_cyt_. Potentially, this could save energy in signalling and H^+^ cycling. This hypothesis could be easily tested.
- There is interaction between K^+^ and Na^+^, which is complicated by the non-selectivity of some channels and transporters for these two cations. However, a functional homeostat system may only allow opposing fluxes through different channels. This means that there must be at least one type of transporter that is highly selective for either K^+^ or Na^+^. Thus, the Na^+^-specific SOS1 might be required on the TP, and a highly selective K^+^ channel is necessary on both the TP and PM. There may also be a need for a selective Na^+^ uniporter on the TP, that is, HKT.
- Cycling H^+^ for homeostatic control is also essential, but this necessitates use of ATP for the H^+^ pumps. Optima, selected during evolution to minimise ATP use, likely exist. This may entail selection for low conductance ion channels, particularly for Na^+^, but also engagement and disengagement of ion homeostats, depending on the prevailing ion gradients.
- The challenge for future models at the cell level is to allow for non-selectivity and direct ion interactions. It might also be possible to maximise ‘cellular fitness’ in models (homeostasis weighted by energy cost), recognising that perfect homeostasis could be too costly and that the cell could tolerate deviations from ideal concentrations if it significantly reduces energy consumption. In this respect, depolarisation of the V_m_ would potentially achieve this outcome, as shown for two cell models for rice cultivars.
- By reframing Na^+^/K^+^ transport models within an optimisation framework, we could gain a powerful new tool for understanding the cellular mechanisms that maintain cellular ionic homeostasis.

## Supporting information

10.1017/qpb.2026.10040.sm001 Tyerman et al. supplementary material 1 Tyerman et al. supplementary material

10.1017/qpb.2026.10040.sm002 Tyerman et al. supplementary material 2 Tyerman et al. supplementary material

## Data Availability

The authors confirm that the data and coding supporting the findings of this study are available within the article and its Supplementary Materials. Raw datasets and further code are available from the corresponding author SDT upon reasonable request. Coding in Python was optimised and debugged using generative AI (Gemini 2.5 Pro and Claude 4).

## Supplementary material

The supplementary material for this article can be found at http://doi.org/10.1017/qpb.2026.10040.
