# Supplementary material for: Cellular aspects of Na+ homeostasis in plants: Quantitative approaches
Source: Quant Plant Biol. 2026 Mar 2;7:e13. doi: 10.1017/qpb.2026.10040 (PMC13280860; doi:10.1017/qpb.2026.10040)
Supplement: Tyerman et al. supplementary material 1 — Tyerman et al. supplementary material [file S263288282610040Xsup001.pdf]

Supplementary Material

1.0 Chloroplast sodium transporters

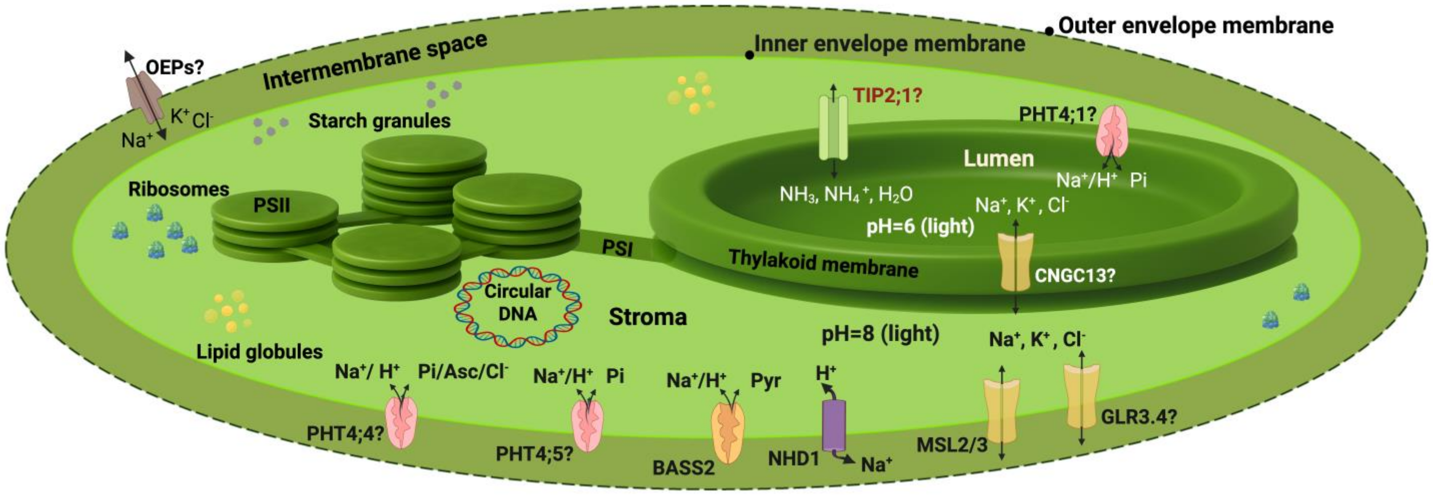

Supplementary Figure S1. Na^+ transporters on the chloroplast membrane. OEPs (Outer Envelope Protein) likely mediate Na^+ transport across the outer envelope membrane (Bölter & Soll, 2001). Members from three families of Na^+-specific transport systems on the inner envelope membrane are proposed as mediating Na^+ influx into the chloroplast lumen, with at least one antiporter responsible for its extrusion. A H^+ extrusion pump on the envelope membrane (Berkowitz & Peters, 1993) regulates stroma pH and energises transport activity (adapted from Bose et al., 2017).

2.1 Basic plasma membrane sodium homeostat, for Figure 2 (main paper).

Equations 35 and 36 from Dreyer et al. (2024) were solved for a range of conductance (*g*) values, as outlined in their paper and applied to the scheme shown in Supplementary Figure 2A (provided in the main paper as Figure 2A but reproduced here for clarity). Rather than using Excel as in Dreyer et al (2024), we used Python code. Coding in Python was optimised and debugged using generative AI (Gemini 2.5 Pro, Claude 4, GTP-5.1). Full scripts are available upon request. All programs were run on a Macbook Pro M1 with the PyCharm IDE. The output from the code shows a similar analysis to that of Dreyer (2021) where the conductances of each of the transporters are plotted against each other, against the steady-state membrane voltage (V_m) (Supplementary Figure 2B) and against the steady-state Na^+ equilibrium potential (E_{Na}) (Supplementary Figure 2C). These are shaded according to the required relative pump current.

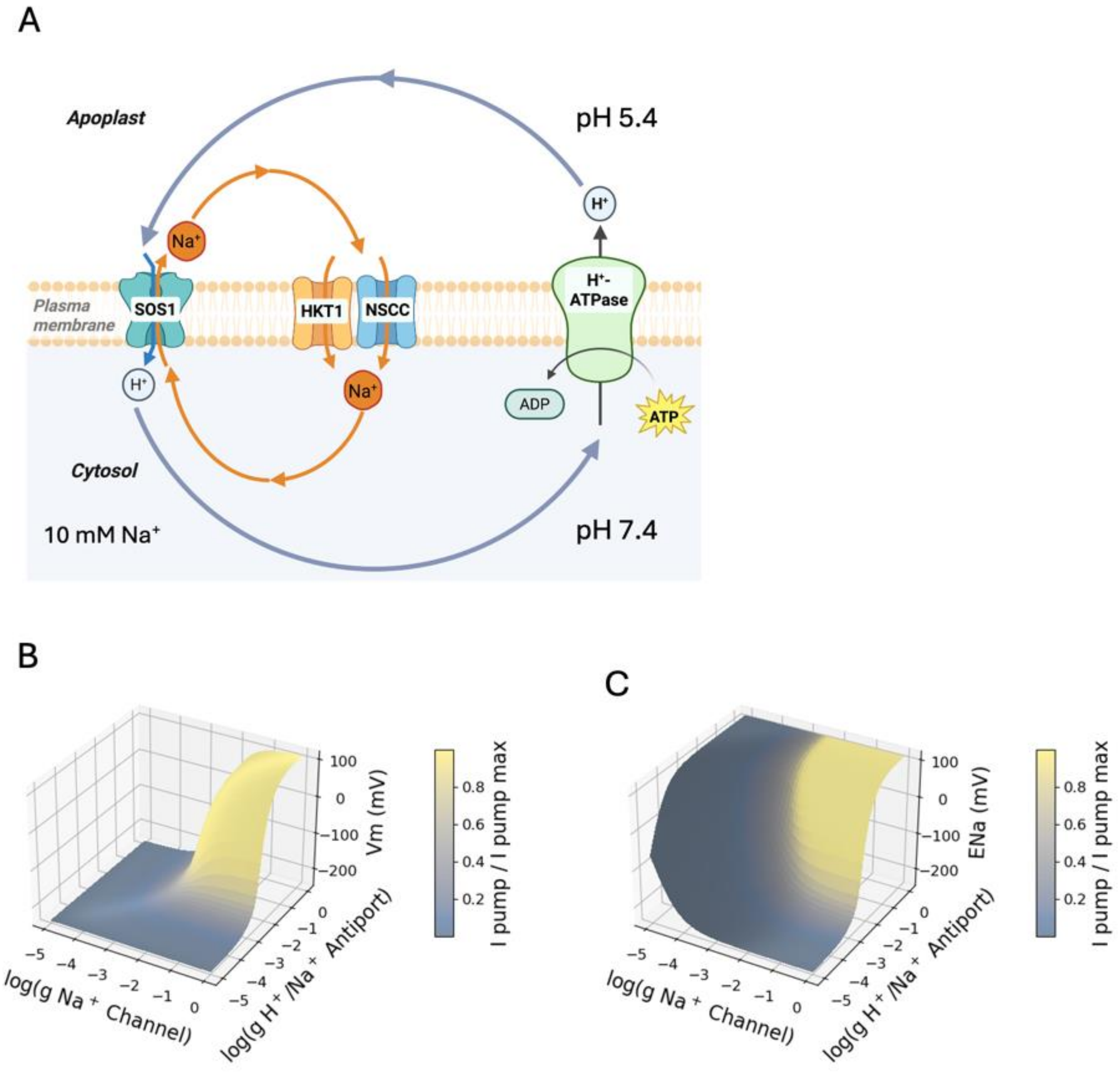

Supplementary Figure S2. Basic Na^+ "homeostat" after Dreyer (2021) and Dreyer et al. (2024) for the steady-state cycles of Na^+ and H^+ unidirectional fluxes across the PM. The scheme in (A) is translated mathematically by setting a range of conductances (*g*) of the SOS1 antiport and HKT1 and/or NSCC uniport (channel) and solving for zero net flux. These are plotted against the steady-state membrane potential (V_m) (B) and steady-state Na^+ equilibrium potential (E_{Na}) (C). They were generated using Equations 35 and 36 from Dreyer et al. (2024), provided below in the Python code and are used for Figure 2 and 3 in the main paper.

It is important to note that these conductances for Na^+ (g, units mV^{-1}) are related to the H^+-ATPase maximum pump current via the relationship: $g = m^*e_0/IH_{max}$, where m is the slope of the tangent to the relevant point on the curve of Na^+ flux versus V_m and has units of mV^{-1} s^{-1}, e_0 is the elementary charge in coulombs and IH_{max} is the maximum current though the H^+-ATPase (coulombs. s^{-1} = amps). This is a useful manipulation to eliminate redundant parameters in the solutions (Dreyer et al., 2024). For the simulations shown, we use the relative pump flux (unitless) as a function of V_m for a wheat root cortical cell according to Tyerman et al. (2001) (see Table 2 and Equation 1 in Tyerman et al., 2001) (Supplementary Figure S3).

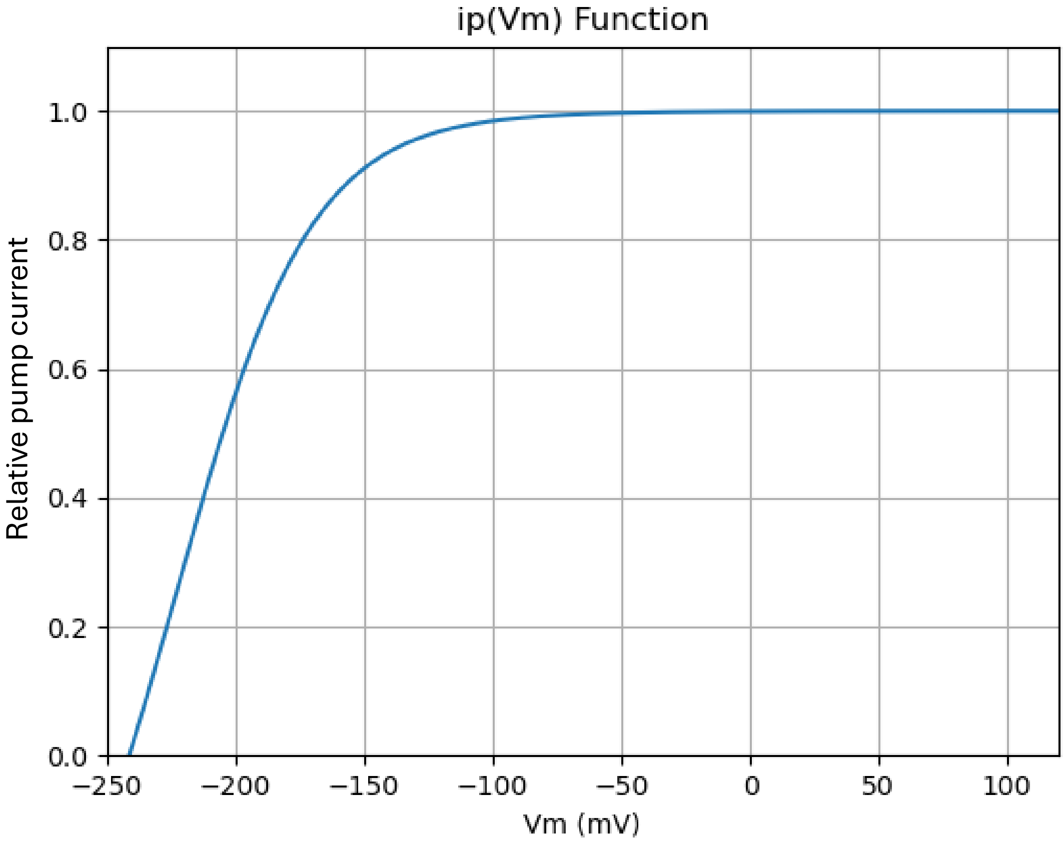

Supplementary Figure S3 for Figures 2 and 3 main paper showing H^+-ATPase function used in simulations, after Tyerman et al. (2001) for wheat root cortical cells protoplasts (Table 2, Equation 1).The equation of the curve is:

$$Ip(rel) \; = \frac{(3000 * e^{0.01946*V} - 0.25 * e^{-0.01946*V})}{(3000 * e^{0.01946*V} + 0.5 * e^{-0.01946*V} + 1.5)}$$

Python Code:

```
import numpy as np
from scipy.optimize import fsolve, brentq
import matplotlib.pyplot as plt
from mpl_toolkits.mplot3d import Axes3D
from matplotlib import cm
import matplotlib.tri as tri  # Import for triangulation
import matplotlib.colors as mcolors  # Import mcolors to use ListedColormap
import matplotlib.ticker as mticker
```

```
from matplotlib.ticker import NullFormatter
import csv # Import the csv library
# Constants (in mV)
EH = 116 # Approximately 2 pH unit gradient across PM, e.g. Cyt pH = pH 7.4  Outside = pH 5.4
Na_in = 10  # Cytoplasmic Na concentration
Vss_target = -120  # membrane potential
epsilon = 1e-12  # A small constant to avoid log(0) issues
koi0 = 0.5 #rate constants for pump
kio0 = 3000
roi = 1 # also for pump from Tyerman et al 2001
rio = 0.5
J3 = 0.0396 # F/RT for Nernst equation in units mV-1 for room temperature
# for range of g values in log ie 10^-5 to 1
start_value = -5
end_value = 0
num_points = 200 # number of points over the range of g values

# Define the function ip(Vss)
# Note the use of kio koi koi0 kio0 here does not indicate potassium rather rate constants
def ipwheat(Vss):
    Vss = np.clip(Vss, -300, EH)
    FVRT = 38.92 * Vss / 2000
    koi = koi0 * np.exp(-FVRT)
    kio = kio0 * np.exp(FVRT)
    return (kio * roi - koi * rio) / (kio + koi + rio + roi)

# Modify the colormap to lighten it for plots
def lighten_colormap(cmap, factor=0.5):
    """Lighten a colormap by the given factor."""
    new_cmap = cmap(np.arange(cmap.N))
    new_cmap[:, :3] += (1 - new_cmap[:, :3]) * factor  # Adjust RGB channels
    new_cmap[new_cmap > 1] = 1  # Ensure no color values exceed 1
    return mcolors.ListedColormap(new_cmap)

#just the Nernst Equation
def calculate_Na_out(Na_in, ENass):
    return Na_in * np.exp(ENass * J3)

def solve_for_Vss(EH, gHNaa, gNaC):
    """
    Solve for the steady-state membrane potential Vss for a given:
      EH    : proton equilibrium potential (mV)
      gHNaa : conductance of the H+/Na+ antiporter (mV-1)
      gNaC  : conductance of the Na+ channel (mV-1)
    Conceptually:
```

```
------------
At steady state, the net current across the membrane is zero:
  I_Na_channel + I_HNaa + I_pump = 0
In this simplified formulation, that steady-state condition has been
rearranged into the implicit equation below (in 'equation(Vss)').
The root of 'equation(Vss)' (i.e., where equation(Vss) = 0)
gives the steady-state membrane potential Vss.
"""
def equation(Vss):
    """
    Implicit steady-state condition for Vm:
      0 = Vss - EH + ipwheat(Vss) * (gNaC + gHNaa) / (gNaC * gHNaa)
    where:
     - Vss - EH
        represents the driving force between membrane potential (Vm = Vss)
        and the proton equilibrium potential (EH), as seen by the H+/Na+
        exchanger term in the underlying derivation.
     - ipwheat(Vss)
        is the normalized pump current (I_pump / I_pump_max) at voltage Vss.
        It is a nonlinear function that increases/decreases with Vss
        based on the kinetic model implemented in ipwheat().
     - (gNaC + gHNaa) / (gNaC * gHNaa)
        comes from combining Na+ channel and H+/Na+ antiporter conductances
        into an effective factor when balancing currents.
        Mathematically it is proportional to (1/gNaC + 1/gHNaa),
        i.e., the sum of "resistances" of the two pathways.
    The root of this function in Vss is the steady-state Vm where
    pump, Na+ channel, and H+/Na+ antiporter fluxes balance.
    """
    return Vss - EH + ipwheat(Vss) * (gNaC + gHNaa) / (gNaC * gHNaa)
# Choose an initial guess for Vss based on the antiporter conductance
# This improves robustness for the fsolve fallback and helps brentq
# (through providing a reasonable expected range).
if gHNaa < 1e-5:
    # Very small H+/Na+ antiporter conductance:
    # membrane tends to be more dominated by other pathways and can sit
    # at a relatively negative potential.
    initial_guess = -150
elif gHNaa > 1e-3:
    # Very large H+/Na+ antiporter conductance:
    # Vm is more strongly clamped near EH, and can be more depolarized.
    initial_guess = 50
else:
    # Intermediate conductance: start near 0 mV as a neutral guess.
    initial_guess = 0
```

```
    try:
        # Primary method: brentq is a robust bracketing root-finder.
        # We search for a root of equation(Vss) in the interval [-250, 150] mV.
        # This requires the function to change sign within this interval.
        Vss_solution = brentq(equation, -250, 150, xtol=1.49012e-10)
        return Vss_solution
    except ValueError:
        # A ValueError here usually means brentq could not find a sign change
        # over the interval [-250, 150]. In that case, we fall back to fsolve,
        # which is not guaranteed to find a root, but can still succeed if the
        # initial guess is close enough to a solution.
        print(f"ValueError with gHNaa={gHNaa:.2e}, gNaC={gNaC:.2e}.  Trying fsolve.")
        try:
            # fsolve is a Newton-like method using 'initial_guess'.
            Vss_solution = fsolve(equation, initial_guess, xtol=1.49012e-12)[0]
            return Vss_solution
        except Exception:
            # If fsolve also fails (e.g., no convergence), we report and return NaN.
            print(f"fsolve failed as well for gHNaa={gHNaa:.2e}, gNaC={gNaC:.2e}")
            return np.nan
    except RuntimeError as e:
        # Catch other numerical issues (e.g., too many iterations) from brentq.
        print(f"RuntimeError: {e} with gHNaa={gHNaa:.2e}, gNaC={gNaC:.2e}")
        return np.nan

# Function to calculate ENass
def calculate_ENass(Vss, gNaC, gHNaa):
    return Vss + ipwheat(Vss) * gHNaa / (gNaC * gHNaa)
# Function to calculate relative fluxes
def calculate_fluxes(gNaC, gHNaa, Vss, ENass, EH):
    JNa_NaC = gNaC * (Vss - ENass)
    JH_HNaa = gHNaa * (ENass - EH)
    JNa_HNaa = -gHNaa * (ENass - EH)
    return JNa_NaC, JH_HNaa, JNa_HNaa
# Range of g values set logarithmically
gHNaa_values = np.logspace(start_value, end_value, num_points)
gNaC_values = np.logspace(start_value, end_value, num_points)
# Create a meshgrid for the g values
gNaC_grid, gHNaa_grid = np.meshgrid(gNaC_values, gHNaa_values)
# Store Vss and ENass values in a grid
Vss_grid = np.zeros_like(gNaC_grid)
ENass_grid = np.zeros_like(gNaC_grid)
Ip_grid = np.zeros_like(gNaC_grid)
# Open CSV file to write data
file_name = 'PM_total_data.csv'
```

```
csv_headers = ['Vss', 'EH', 'ENass', 'ip_wheat(Vss)', 'gNaCss', 'gHNaass', 'JNaC', 'JHNa']
with open(file_name, 'w', newline='') as csvfile:
    writer = csv.writer(csvfile)
    writer.writerow(csv_headers)
    # Loop over each combination of gHNaa and gNaC
    for i in range(num_points):
        for j in range(num_points):
            gNaC = gNaC_grid[i, j]
            gHNaa = gHNaa_grid[i, j]
            Vss = solve_for_Vss(EH, gHNaa, gNaC)
            if np.isnan(Vss):
                Vss_grid[i, j] = np.nan
                ENass_grid[i, j] = np.nan
                Ip_grid[i, j] = np.nan
                # Prepare data for CSV row
                ENass, ip_wheat_val, JNaC, JHNa = [np.nan] * 4
            else:
                ENass = calculate_ENass(Vss, gNaC, gHNaa)
                Vss_grid[i, j] = Vss
                ENass_grid[i, j] = ENass
                Ip_grid[i, j] = ipwheat(Vss)  # Original calculation for plot data
                # Calculate additional values for CSV output
                ip_wheat_val = ipwheat(Vss)
                JNaC = gNaC * (Vss - ENass)
                JHNa = -gHNaa * (ENass - EH)
            # Write the data row to the CSV file
            writer.writerow([Vss, EH, ENass, ip_wheat_val, gNaC, gHNaa, JNaC, JHNa])
print('Output written to:' + file_name)
light_cividis = lighten_colormap(cm.cividis)
# Plotting
# Set global font sizes for this plot
plt.rcParams.update({
    'font.size': 12, 'axes.titlesize': 18, 'axes.labelsize': 16,
    'xtick.labelsize': 12, 'ytick.labelsize': 12, 'legend.fontsize': 12,
    'figure.titlesize': 20
})
fig = plt.figure(figsize=(16, 12))  # Adjusted figure size for 2x2 grid
# First subplot: 3D surface plot of Vss with color representing ip(Vss)
ax1 = fig.add_subplot(221, projection='3d')
# Use nanmin and nanmax to handle potential NaN values from failed solves
nan_mask = np.isnan(Ip_grid)
normalized_ip = np.full_like(Ip_grid, 0.5)  # Default color for NaN
if not np.all(nan_mask):
    ip_min, ip_max = np.nanmin(Ip_grid), np.nanmax(Ip_grid)
    if ip_max > ip_min:
```

```
    normalized_ip[~nan_mask] = (Ip_grid[~nan_mask] - ip_min) / (ip_max - ip_min)
# Create a color map based on normalized ip values
colors = light_cividis(normalized_ip)  # Use the colormap to get colors
# Plot the surface with Vss on the z-axis
surf1 = ax1.plot_surface(np.log10(gNaC_grid), np.log10(gHNaa_grid), Vss_grid,  # Vss on the z-axis
                        facecolors=colors,  # Use the created colors from the colormap
                        linewidth=0, antialiased=False)
ax1.set_xlabel('log(g Na$^+$ Channel)', labelpad=10)
ax1.set_ylabel('log(g H$^+$/Na$^+$ Antiport)', labelpad=10)
ax1.set_zlabel('Vm (mV)', labelpad=10)
# Adding a color bar for the ip(Vss)
if not np.all(nan_mask):
    norm = plt.Normalize(np.nanmin(Ip_grid), np.nanmax(Ip_grid))
    cbar1 = fig.colorbar(plt.cm.ScalarMappable(norm=norm, cmap=light_cividis),
                        ax=ax1, shrink=0.5, aspect=10, pad=0.2)  # Increase pad to move colorbar
    cbar1.set_label('I pump / I pump max')  # Label for the color bar
# Second subplot: 3D surface plot of ENass with color representing normalized ip(Vss)
ax2 = fig.add_subplot(222, projection='3d')
surf2 = ax2.plot_surface(np.log10(gNaC_grid), np.log10(gHNaa_grid), ENass_grid,
                        facecolors=colors,  # Use the same normalized_ip for color
                        linewidth=0, antialiased=False)
ax2.set_xlabel('log(g Na$^+$ Channel)', labelpad=10)
ax2.set_ylabel('log(g H$^+$/Na$^+$ Antiport)', labelpad=10)
ax2.set_zlabel('ENa (mV)', labelpad=10)
# Color bar for normalized ip(Vss)
if not np.all(nan_mask):
    norm_ip = plt.Normalize(np.nanmin(Ip_grid), np.nanmax(Ip_grid))
    cbar2 = fig.colorbar(plt.cm.ScalarMappable(norm=norm_ip, cmap=light_cividis),
                        ax=ax2, shrink=0.5, aspect=10, pad=0.2)
    cbar2.set_label('I pump / I pump max')  # Label for the color bar
# Third subplot: 2D contour plot of ip(Vss) vs Vss and ENass
ax3 = fig.add_subplot(223)
vss_flat = Vss_grid.flatten()
enass_flat = ENass_grid.flatten()
ip_flat = Ip_grid.flatten()
nan_mask_flat = np.isnan(vss_flat) | np.isnan(enass_flat) | np.isnan(ip_flat)
vss_flat = vss_flat[~nan_mask_flat]
enass_flat = enass_flat[~nan_mask_flat]
ip_flat = ip_flat[~nan_mask_flat]
if len(vss_flat) > 3:  # Need at least 3 points for triangulation
    vss_min = np.min(vss_flat)
    vss_max = np.max(vss_flat)
    triang = tri.Triangulation(vss_flat, enass_flat)
    contour = ax3.tricontourf(triang, ip_flat, cmap=light_cividis)
    # Color bar for contour plot
```

```
    cbar3 = fig.colorbar(contour, ax=ax3)
    cbar3.set_label('I pump / I pump max')  # Label for the color bar
    # Line Vss = ENass
    vss_range = np.linspace(max(-200, vss_min), min(150, vss_max), 50)
    ax3.plot(vss_range, vss_range, 'r--', label='Vm = ENa')
    # Draw a horizontal dashed line for EH
    ax3.axhline(y=EH, color='b', linestyle='--', label=f'EH = {EH} mV')
    ax3.legend(loc='lower left')
ax3.set_xlabel('Vm (mV)', x=0.8)
ax3.set_ylabel('ENa (mV)', position=(-0.1, 1.0), ha='right', va='bottom')
ax3.set_ylim([-250, 150])
ax3.grid(True)  # Enable grid
ax3.spines['left'].set_position('zero')
ax3.spines['bottom'].set_position('zero')
ax3.spines['right'].set_color('none')
ax3.spines['top'].set_color('none')
ax3.xaxis.set_ticks_position('bottom')
ax3.yaxis.set_ticks_position('left')
# Fourth subplot: Ip, gNaC, and gHNaa as a function of ENass
ax4 = fig.add_subplot(224)
vss_mask = np.abs(Vss_grid - Vss_target) < 2
enass_values = ENass_grid[vss_mask]
ip_values = Ip_grid[vss_mask]
gnac_values = gNaC_grid[vss_mask]
ghnaa_values = gHNaa_grid[vss_mask]
# Check if any data points match the criteria before proceeding
if enass_values.size > 0:
    # Sort values for plotting
    sort_indices = np.argsort(enass_values)
    enass_values = enass_values[sort_indices]
    ip_values = ip_values[sort_indices]
    gnac_values = gnac_values[sort_indices]
    ghnaa_values = ghnaa_values[sort_indices]
    JNaC_values = gnac_values * (Vss - enass_values)
    JHNa_values = -ghnaa_values * (enass_values - EH)
    # Calculate Na_out using the Nernst function
    Na_out_values = calculate_Na_out(Na_in, enass_values)
    # CSV EXPORT
    output_filename = 'PM_Na_out.csv'
    with open(output_filename, 'w', newline='') as csvfile:
        writer = csv.writer(csvfile)
        # First header row
        writer.writerow(['', '', '', '', '', '', '',
                         f'For Vss =', Vss_target, f'[Na$^+$]cyt =', Na_in])
        # Second row with data headers
```

```
        data_headers = ['ENa (mV)', 'ip', 'gNaC', 'gHNa', 'JNaC', 'JHNa', '[Na]ext']
        writer.writerow(data_headers)
        # Data rows
        data_rows = zip(enass_values, ip_values, gnac_values, ghnaa_values,
                JNaC_values, JHNa_values, Na_out_values)
        writer.writerows(data_rows)
    print(f"Plot data for subplot 4 successfully exported to {output_filename}")
    # Plot the values
    ax4.plot(enass_values,
         np.log10(np.abs(ip_values) + epsilon),
         label='log10(|I Pump/I Pump max|)', color='b', linestyle='-')
    ax4.plot(enass_values,
         np.log10(gnac_values + epsilon),
         label='log10(g Na$^+$ Channel)', color='g', linestyle='--')
    ax4.plot(enass_values,
         np.log10(ghnaa_values + epsilon),
         label='log10(g H$^+$/Na$^+$ Antiport)', color='r', linestyle=':')
    # Plot Na_out on a secondary y-axis
    ax4_secondary = ax4.twinx()  # Create a secondary y-axis
    ax4_secondary.set_yscale('log')
    ax4_secondary.yaxis.set_major_formatter(mticker.ScalarFormatter())
    ax4_secondary.yaxis.set_minor_formatter(NullFormatter())
    ax4_secondary.set_ylabel('[Na$^+$]$_{ext}$ (mM, log scale)', color='black')
    ax4_secondary.tick_params(axis='y', labelcolor='saddlebrown')
    ax4_secondary.plot(enass_values, Na_out_values,
              label='[Na$^+$]$_{ext}$ mM', color='black',
              linestyle='--')
else:
    # If no data is found, print a message
    print(f"Warning: No data found for Vm ≈ {Vss_target} mV. Subplot 4 and CSV export will be empty.")
# Labels and titles
ax4.set_xlabel('ENa (mV)')
ax4.set_ylabel('Log10(Values)')
ax4.legend(loc='upper left', bbox_to_anchor=(0.1, 0.95))
# Optionally adjust y-limits for secondary y-axis
# Make adjustments to ensure Na_out values are displayed properly
ymin_naout = np.min(Na_out_values, initial=1)
ymax_naout = np.max(Na_out_values, initial=1)
ax4_secondary.set_ylim((ymin_naout, ymax_naout))
ax4.grid(axis='x')         # Enable vertical grid lines for the x-axis on the primary axis
ax4_secondary.grid(axis='y')  # Enable horizontal grid lines for the Y-axis on the secondary axis
# Save the plot
plot_file = 'PMV4_plot_Fig2.png'
plt.savefig(plot_file, format='png')
print("Plot saved as: " + plot_file)
```

```python
# Show the plot
plt.show()
```

2.2 Combining Na^+ and K^+ homeostats:

In the first simulation, we consider Na^+ transport across the PM together with K^+ transport when the cell is at steady-state. It comprises a K^+:symport (1:1 H^+:K^+), a K^+ channel, a Na^+ antiport (1:1 H^+:Na^+) and a Na^+ channel.

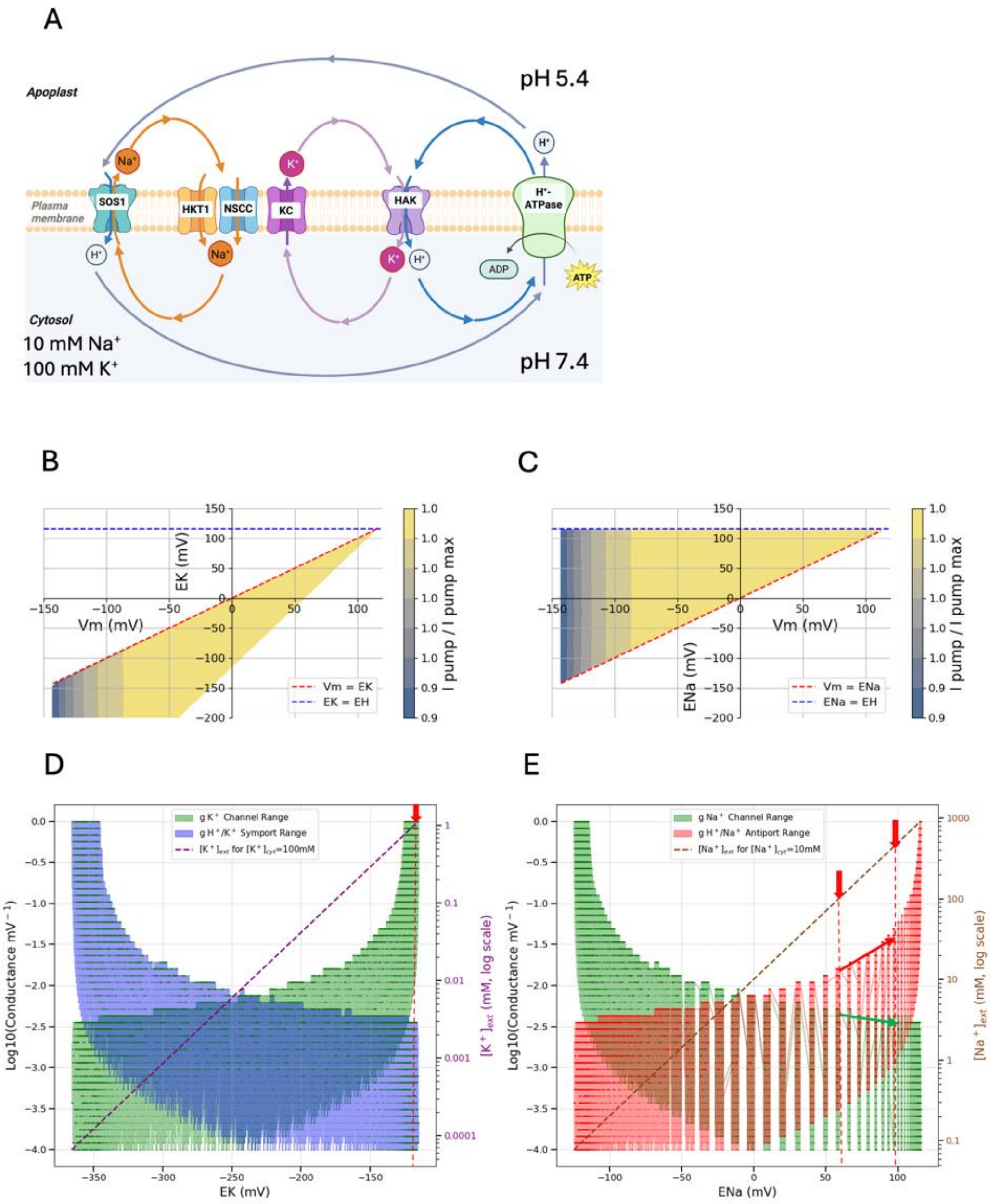

Supplementary Figure S4. (A) An additional homeostat for K^+ is combined with that for Na^+, as shown in Supplementary Figure S2. A K^+:H symport drives K^+ uptake (e.g., a HAK), utilising the H^+ gradient, and the return path for K^+ is via a K^+ channel (KC) such as GORK. These combined homeostats serve to illustrate the interactions between K^+ and Na^+ transport. The constraints on E_K and E_{Na} are shown in (B) and (C) for the scheme shown in (A). For a K^+:H^+ symport (1 H^+:1 K), E_K cannot be positive of V_m at steady-state (B) while E_{Na} (C) is constrained as before. The minimum V_m is less negative with the added transports (compare with Figure 2 B,C, main paper). (D) The conductances (log scale) of the K^+ symport and K^+ channel are plotted against E_K for the situation where V_m is held at -120 mV, $[K^+]_{cyt}$ = 100 mM, $[Na^+]_{cyt}$ = 10 mM and E_H = 116 mV. The dashed diagonal line indicates the $[K^+]_{ext}$ as a function of E_K for these constraints. With this K^+ homeostat, the system cannot operate when $[K^+]_{ext}$ is high (contrast with Figure 3, main paper) and the flux through the K^+ channel is always outward. (E) shows conductances for the Na^+ antiport and Na^+ channel as a function of E_{Na} with the same constraints as for (D). The diagonal dashed line indicates $[Na^+]_{ext}$ for perfect $[Na^+]_{cyt}$ (10 mM) homeostasis and the associated changes in *g* values required to achieve this at steady-state. The red arrows indicate where $[Na^+]_{ext}$ = 100 mM and 300 mM, and the change required between steady-states is indicated by the red arrow for the antiport and the green arrow for the channel. Note that with the combined solutions of the flux equations, there are a range of solutions for *g* values at each E_K and E_{Na} in (D) and (E), which is dependent on the proportion of the H^+ flux allocated to each homeostat.

Supplementary. Figure S4 B,C,D,E were generated from the steady-state solutions where:

1. **Net K^+ Flux = 0:**

$g_{Kc}*(V_{ss} - E_{Kss}) + g_{HKs}*(2*V_{ss} - E_H - E_{Kss}) = 0$

2. **Net Na^+ Flux = 0:**

$g_{Nac}*(V_{ss} - E_{Nass}) + g_{HNaa}*(E_H - E_{Nass}) = 0$

3. **Net H^+ Flux = 0:**

$ip(V_{ss}) + g_{HKs}*(2*V_{ss} - E_H - E_{Kss}) + g_{HNaa}*(E_{Nass} - E_H) = 0$

- **Net K^+ Flux = 0:** Describes the balance of K^+ fluxes and considers channels and symport
- **Net Na^+ Flux = 0:** Describes the balance of Na^+ fluxes, considering channels and antiport
- **Net H^+ Flux = 0:** Describes the balance of H^+ fluxes

$ip(V_{ss})$ represents a voltage-dependent H^+ pump, relative to maximum (unitless). Conductances are g_{Kc} (K^+ channel), g_{HKs} (H^+:K^+ symporter 1:1), g_{Nac} (Na^+ channel), g_{HNaa} (H^+:Na^+ antiporter 1:1) all with units mV^{-1}. V_{ss} (steady-state membrane voltage, mV), E_{Kss} (steady-state K^+ equilibrium potential, mV), E_{Nass} (steady-state Na^+ equilibrium potential, mV), E_H (given H^+ equilibrium potential, mV).

For row 1:

$(g_{Kc} + 2*g_{HKs})*V_{ss} + (- g_{Kc} - g_{HKs})*E_{Kss} + (0)*E_{Nass} = (g_{HKs})*E_H$

For row 2:

$(g_{NaC})*V_{ss} + (0)*E_{Kss} + (- g_{NaC} - g_{HNaa})*E_{Nass} = - g_{HNaa}*E_H$

For row 3:

$(2*g_{HKs})*V_{ss} + (- g_{HKs})*E_{Kss} + (g_{HNaa})*E_{Nass} = (g_{HKs} + g_{HNaa})*E_H - ip(V_{ss})$

Matrix:

M=

Row 1: $[(g_{Kc} + 2*g_{HKs}), (- g_{Kc} - g_{HKs}), 0]$

Row 2: $[(g_{NaC}), 0, (- g_{NaC} - g_{HNaa})]$

Row 3: $[(2*g_{HKs}), (- g_{HKs}), (g_{HNaa})]$

Vector of unknowns:

X =[V_{ss}

EK_{ss}

E_{Nass}]

Vector of constants:

A= [$(g_{HKs})*E_H$

$- g_{HNaa}*E_H$

$(g_{HKs} + g_{HNaa})*E_H - ip(V_{ss})$]

Note $ip(V_{ss})$ is the pump current set to max = 1 and solved iteratively for each V_{ss}

M: is a 3 x 3 matrix of co-efficients derived from the conductances (*g* values) of the different transporters

X: is a vector containing the unknowns: V_{ss}, E_{Kss} and E_{Nass}. These represent the steady-state membrane potential, the K^+ Nernst potential and the Na^+ Nernst potential, respectively

A: is a vector containing terms related to the driving forces of the ions.

Python Code:

Solve_equation function**:**

The function takes the conductances (g_{KC}, g_{NaC}, g_{HKs}, g_{HNaa}), the H^+ Nernst potential (E_H), the H^+ pump function (ip_func), an initial estimate for membrane potential (initial_V_{ss}), a tolerance, and a maximum iteration limit as input.

The coding takes an iterative approach to solve the system of equations.

Inside the for loop:

- It updates the ip_V_{ss} variable by calling ip*func(Vss)* to account for the non-linearity of the H^+ pump.
- Uses numpy.linalg.solve(M, A) to solve the linear system of equations. This gives a solution vector X containing updated estimates for V_{ss}, E_{Kss} and E_{Nass}.
- It checks for convergence by comparing the new V_{ss} with the previous one (`abs(Vssnew - Vss) < tolerance). If the difference is less than the tolerance, it considers the solution converged and returns the values.
- If the LinAlgError exception is raised, then it indicates that the equations could not be solved. will return None.
- If the maximum number of iterations is reached without convergence, the function returns None.

H^+ pump Function (ip(V_{ss})):

- This function defines the voltage-dependent H^+ pump current, ip(V_{ss}).
- The code uses the Nernst equation for the H^+ ion distribution and computes the relative rate of the pump as a function of membrane voltage V_{ss}.
- koi, kio, roi, and rio represent voltage-dependent rate constants for the pump taken from Tyerman et al. (2001) for wheat root cortex cell protoplast.

Parameter Settings:

The code sets several parameters including:

E_H: the H^+ Nernst potential.

koi0 and kio0: parameters related to the voltage dependence of the H^+ pump.

roi and rio: relative rates related to the pump.

num_points over each *g* value over a log scale. Specifies the number of points to use in the simulation.

Code (minus plotting):

```python
import numpy as np
import csv
import scipy
import matplotlib.pyplot as plt
import matplotlib.ticker as mticker
from scipy.interpolate import griddata
import matplotlib.tri as tri
from matplotlib import cm
import pandas as pd
from matplotlib.ticker import LogLocator, LogFormatter

print('K + Na, Channel and Antiport, + K:H Symport, n=1, Plasmamembrane, Na and K independent (gHKa removed)')

def solve_equations(gKC, gNaC, gHKs, gHNaa, EH, ip_func, initial_Vss=-100.0, tolerance=1e-12, max_iter=100):
    """
    Iteratively solves the system of equations.
    """
    Vss = initial_Vss
    for i in range(max_iter):
        M = np.array([
            # K Balance: gKc*(Vss-EKss) + gHKs*(2*Vss-EH-EKss) = 0
            # (gKC + 2*gHKs)*Vss + (-(gKC + gHKs))*EKss + (0)*ENaSS = gHKs*EH
            [gKC + 2 * gHKs, -(gKC + gHKs), 0],
            # Na Balance: gNaC*(Vss-ENass) + gHNaa*(EH-ENass) = 0
            # (gNaC)*Vss + (0)*EKss + (-(gNaC + gHNaa))*ENaSS = -gHNaa*EH
            [gNaC, 0, -(gNaC + gHNaa)],
            # H Balance: ip(Vss) + gHKs*(2*Vss-EH-EKss) + gHNaa*(ENass-EH) = 0
            # (2*gHKs)*Vss + (-gHKs)*EKss + (gHNaa)*ENaSS = (gHKs + gHNaa)*EH - ip(Vss)
            [2 * gHKs, -gHKs, gHNaa]
        ])
        ip_Vss = ip_func(Vss)
        A = np.array([
            # RHS of K+ flux eq
            gHKs * EH,
            # RHS of Na+ flux eq
            -gHNaa * EH,
            # RHS of H+ flux eq
            (gHKs + gHNaa) * EH - ip_Vss
        ])

        try:
            X = np.linalg.solve(M, A)
            Vss_new, EKss, ENaSS = X
            if abs(Vss_new - Vss) < tolerance:
```

```
            return Vss_new, EKss, ENaSS
        Vss = Vss_new
    except np.linalg.LinAlgError:
        return None
    return None


if __name__ == '__main__':
    # ---------------------------------------------------------------------
    # --- Simulation and Raw Data Generation ---
    # ---------------------------------------------------------------------
    # Define parameters
    Vss_target = -120
    Vss_tolerance = 5
    K_in = 100
    Na_in = 10
    EH = 116
    koi0 = 0.5
    kio0 = 700
    roi = 1
    rio = 0.5
    num_points = 20
    g_values = np.logspace(-4, 0, num_points)

    def ip(Vss):
        FVRT = 38.92 * Vss / 2000
        koi = koi0 * np.exp(-FVRT)
        kio = kio0 * np.exp(FVRT)
        return (kio * roi - koi * rio) / (kio + koi + rio + roi)

    filename = "../Modeling membrane fluxes/flux_results.csv"  # Updated filename

    # Header
    header = ["gKC", "gNaC", "gHKs", "gHNaa", "Vss", "EKss", "ENaSS",
              "JKc", "JNaC", "JKHs", "JNaH", "ip"]

    # total_iterations reflects 4 nested loops
    total_iterations = len(g_values) ** 4
    print_interval = max(1, total_iterations // 10)  # Print ~100 updates, adjusted for larger num_points
    iteration_count = 0
    with open(filename, mode='w', newline='') as csvfile:
        writer = csv.writer(csvfile)
        writer.writerow(header)
        for gKC in g_values:
            for gNaC in g_values:
```

```
            for gHNaa in g_values:
                for gHKs in g_values:
                    iteration_count += 1
                    if iteration_count % print_interval == 0:
                        percentage_complete = (iteration_count / total_iterations) * 100
                        print(
                            f"Progress: {percentage_complete:.2f}% complete ({iteration_count}/{total_iterations})",
                            flush=True)
                    result = solve_equations(gKC, gNaC, gHKs, gHNaa, EH, ip)
                    if result:
                        Vss, EKss, ENaSS = result
                        JKc = gKC * (Vss - EKss) # K channel flux
                        JNaC = gNaC * (Vss - ENaSS) # Na channel flux
                        JKHs = gHKs * (2 * Vss - EH - EKss)  # K/H Symporter flux
                        JNaH = -gHNaa * (ENaSS - EH) # Na/H Antiporter flux
                        ip_val = ip(Vss)
                        row = [gKC, gNaC, gHKs, gHNaa, Vss, EKss, ENaSS,
                               JKc, JNaC, JKHs, JNaH, ip_val]
                        writer.writerow(row)
    print(f"\nFull simulation data written to {filename}")
```

Plotting routines not shown.

2.3 Combined K^+ and Na^+ homeostats incorporating a K^+:H^+ antiport in addition to a K^+ channel and K^+:H^+ symport. For Figure 3 (Main paper).

Na^+ and K^+ together, moving in a cycle across the PM at steady-state. A K^+:symport (1:1 H^+:K^+), K^+:antiport (1:1 H^+:K^+), K^+ channel, Na^+ antiport (1:1 H^+:Na^+), Na^+ channel.

Figure 3 (Main paper) was generated from the steady-state solutions where:

1. **Net K^+ Flux = 0:**

$$g_{Kc}*(V_{ss} - E_{Kss}) + g_{HKa}*(E_H - E_{Kss}) + g_{HKs}*(2*V_{ss} - E_H - E_{Kss}) = 0$$

2. **Net Na^+ Flux = 0:**

$$g_{Nac}*(Vss - E_{Nass}) + g_{HNaa}*(E_H - E_{Nass}) = 0$$

3. **Net H^+ Flux = 0:**

$$ip(V_{ss}) + g_{HKa}*(E_{Kss} - E_H) + g_{HKs}*(2*V_{ss} - E_H - E_{Kss}) + g_{HNaa}*(E_{Nass} - E_H) = 0$$

• **Net K^+ Flux = 0:** Describes the balance of K^+ fluxes. Considers channels, symport and antiport

• **Net Na^+ Flux = 0:** Describes the balance of Na^+ fluxes. Considers channels and antiport

• **Net H^+ Flux = 0:** Describes the balance of H^+ fluxes

$ip(V_{ss})$ represents a voltage-dependent H^+ pump. Conductances are g_{Kc} (K^+ channel), g_{HKa} (H^+:K^+ antiporter 1:1), g_{HKs} (H^+:K^+ symporter 1:1), g_{Nac} (Na^+ channel), g_{HNaa} (H^+:Na^+ antiporter 1:1) all with units mV^{-1}. V_{ss} (steady-state membrane voltage, mV), E_{Kss} (steady-state K^+ equilibrium potential, mV), E_{Nass} (steady-state Na^+ equilibrium potential, mV), E_H (given H^+ equilibrium potential, mV).

For row 1:

$$(g_{Kc} + 2*g_{HKs})*V_{ss} + (- g_{Kc} - g_{HKa} - g_{HKs})*E_{Kss} + (0)*E_{Nass} = (g_{HKs} - g_{HKa})*E_H$$

For row 2:

$$(g_{NaC})*V_{ss} + (0)*E_{Kss} + (- g_{NaC} - g_{HNaa})*E_{Nass} = - g_{HNaa}*E_H$$

For row 3:

$$(2*g_{HKs})*V_{ss} + (g_{HKa} - g_{HKs})*E_{Kss} + (g_{HNaa})*E_{Nass} = (g_{HKa} + g_{HKs} + g_{HNaa})*E_H - ip(V_{ss})$$

Matrix:

M=

Row 1: $[(g_{Kc} + 2*g_{HKs}), (- g_{Kc} - g_{HKa} - g_{HKs}), 0]$

Row 2: $[(g_{NaC}), 0, (- g_{NaC} - g_{HNaa})]$

Row 3: $[(2*g_{HKs}), (g_{HKa} - g_{HKs}), (g_{HNaa})]$

Vector of unknowns:

X =$[V_{ss}$

E_{Kss}

$E_{Nass}]$

Vector of constants:

A= $[(g_{HKs} - g_{HKa})*E_H$

- g_{HNaa}*E_H

(g_{HKa} + g_{HKs} + g_{HNaa})*E_H - ip(V_{ss})]

Note ip(V_{ss}) is the pump current set to max = 1 and solved iteratively for each V_{ss}.

M: is a 3 x 3 matrix of co-efficients derived from the conductances (g values) of the different transporters.

X: is a vector containing the unknowns: V_{ss}, E_{Kss}, and E_{Nass}. These represent the steady-state membrane potential, the K^+ Nernst potential, and the Na^+ Nernst potential, respectively.

A: is a vector containing terms related to the driving forces of the ions.

Python Code:

Solve_equation function:

The function takes the conductances (g_{KC}, g_{NaC}, g_{HKa}, g_{HKs}, g_{HNaa}), the H^+ Nernst potential (E_H), the H^+ pump function (ip_func), an initial estimate for membrane potential (initial_V_{ss}), a tolerance, and a maximum iteration limit as input.

Takes an iterative approach to solve the system of equations.

The H^+ pump (ip_func) is voltage-dependent and non-linear.

Constructs the matrix M and vector A as described above using numpy.array.

Inside the for loop:

- It updates the ip_V_{ss} variable by calling ip*func(Vss)* to account for the non-linearity of the H^+ pump.
- Uses numpy.linalg.solve(M, A) to solve the linear system of equations. This gives a solution vector X containing updated estimates for V_{ss}, E_{Kss} and E_{Nass}.
- It checks for convergence by comparing the new V_{ss} with the previous one (`abs(Vssnew - Vss) < tolerance). If the difference is less than the tolerance, it considers the solution converged and returns the values.
- If the LinAlgError exception is raised, then it indicates that the equations could not be solved. will return None.
- If the maximum number of iterations is reached without convergence, the function returns None.

H^+ pump Function (ip(V_{ss})):

- This function defines the voltage-dependent H^+ pump current, ip(V_{ss}).
- The code uses the Nernst equation for the H^+ ion distribution and computes the relative rate of the pump as a function of membrane voltage V_{ss}.
- koi, kio, roi, and rio represent voltage-dependent rate constants for the pump taken from Tyerman et al. (2001) for wheat root cortex cell protoplast.

Parameter Settings:

The code sets several parameters including:

E_H: the H^+ Nernst potential.

koi0 and kio0: parameters related to the voltage dependence of the H^+ pump.

roi and rio: relative rates related to the pump.

num_points over each *g* value over a log scale. Specifies the number of points to use in the simulation.

Code (minus plotting):

```
import numpy as np
import csv
import scipy
import matplotlib.pyplot as plt
import matplotlib.ticker as mticker
from scipy.interpolate import griddata
import matplotlib.tri as tri
from matplotlib import cm
import pandas as pd
from matplotlib.ticker import LogLocator, LogFormatter

print('K + Na, Channel and Antiport, + K:H Symport,n=1, Plasmamembrane, Na and K independent')

def solve_equations(gKC, gNaC, gHKa, gHKs, gHNaa, EH, ip_func, initial_Vss=-100.0, tolerance=1e-12,
max_iter=100):
    """
    Iteratively solves the system of equations
    """
    Vss = initial_Vss
    for i in range(max_iter):
        # matrix M including
        M = np.array([
            # Row 1: K+ fluxes (gKc, gHKa, gHKs)
            [gKC + 2 * gHKs, -(gKC + gHKa + gHKs), 0],
            # Row 2: Na+ fluxes (gNaC, gHNaa)
            [gNaC, 0, -(gNaC + gHNaa)],
            # Row 3: H+ fluxes (gHKa, gHKs, gHNaa, ip)
            [2 * gHKs, gHKa - gHKs, gHNaa]
        ])

        ip_Vss = ip_func(Vss)

        A = np.array([
            # RHS of K+ flux eq
            (gHKs - gHKa) * EH,
            # RHS of Na+ flux eq
            -gHNaa * EH,
            # RHS of H+ flux eq
            (gHKa + gHKs + gHNaa) * EH - ip_Vss
        ])
```

```
        try:
            X = np.linalg.solve(M, A)
            Vss_new, EKss, ENaSS = X
            if abs(Vss_new - Vss) < tolerance:
                return Vss_new, EKss, ENaSS
            Vss = Vss_new
        except np.linalg.LinAlgError:
            return None
    return None


if __name__ == '__main__':
    # ------------------------------------------------------------------
    # --- Simulation and Raw Data Generation ---
    # ------------------------------------------------------------------
    # Define parameters
    Vss_target = -120
    Vss_tolerance = 5
    K_in = 100
    Na_in = 10
    EH = 116
    koi0 = 0.1
    kio0 = 100
    roi = 1
    rio = 0.5
    num_points = 20
    g_values = np.logspace(-4, 0, num_points)
    def ip(Vss):
        FVRT = 38.92 * Vss / 2000
        koi = koi0 * np.exp(-FVRT)
        kio = kio0 * np.exp(FVRT)
        return (kio * roi - koi * rio) / (kio + koi + rio + roi)
    # Lists to store valid results for plotting

    filename = "flux_results.csv"

    header = ["gKC", "gNaC", "gHKa", "gHKs", "gHNaa", "Vss", "EKss", "ENaSS",
              "JKc", "JNaC", "JKH", "JKHs", "JNaH", "ip"]

    total_iterations = len(g_values) ** 5
    print_interval = max(1, total_iterations // 10)  # Print ~10 updates
    iteration_count = 0
    with open(filename, mode='w', newline='') as csvfile:
        writer = csv.writer(csvfile)
        writer.writerow(header)
```

```
    for gKC in g_values:
        for gNaC in g_values:
            for gHNaa in g_values:
                for gHKa in g_values:
                    for gHKs in g_values:
                        iteration_count += 1
                        if iteration_count % print_interval == 0:
                            percentage_complete = (iteration_count / total_iterations) * 100
                            print(
                                f"Progress: {percentage_complete:.2f}% complete ({iteration_count}/{total_iterations})",
                                flush=True)

                        result = solve_equations(gKC, gNaC, gHKa, gHKs, gHNaa, EH, ip)

                        if result:
                            Vss, EKss, ENaSS = result
                            JKc = gKC * (Vss - EKss)
                            JNaC = gNaC * (Vss - ENaSS)
                            JKH = -gHKa * (EKss - EH)
                            JKHs = gHKs * (2 * Vss - EH - EKss)
                            JNaH = -gHNaa * (ENaSS - EH)
                            ip_val = ip(Vss)

                            row = [gKC, gNaC, gHKa, gHKs, gHNaa, Vss, EKss, ENaSS,
                                   JKc, JNaC, JKH, JKHs, JNaH, ip_val]
                writer.writerow(row)

print(f"\nFull simulation data written to {filename}")
```

2.4 Grouping of conductances at E_{Na} when combining K^+ and Na^+ homeostats.

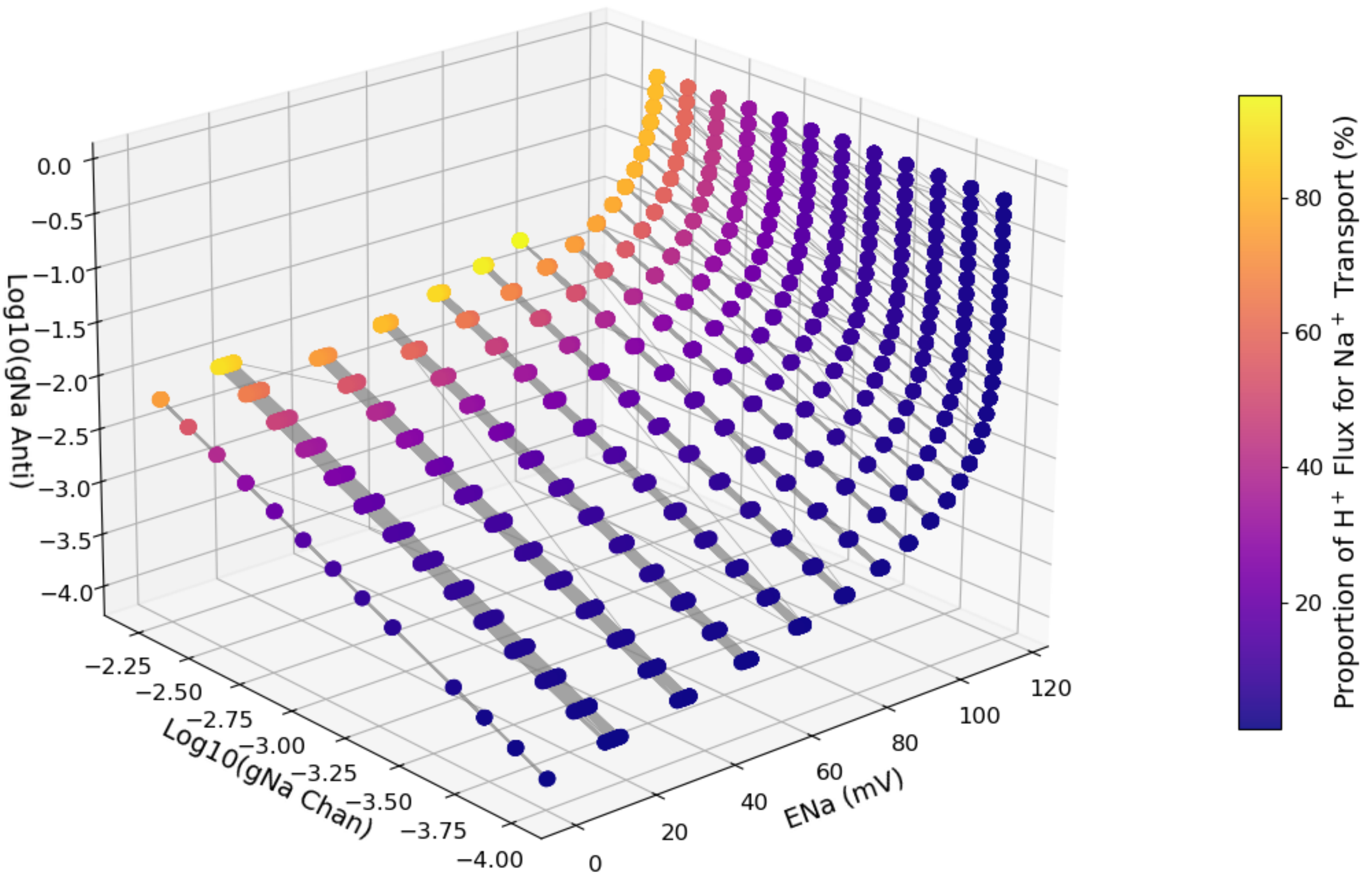

Supplementary Figure S5. For Figure 3D (main paper) showing grouping of solutions for *g* values at E_{Na} and the effect of the proportion of H^+ cycling via the Na^+ homeostat.

2.5 Effect of depolarising the clamped membrane potential in the model shown in Figure 3 main paper.

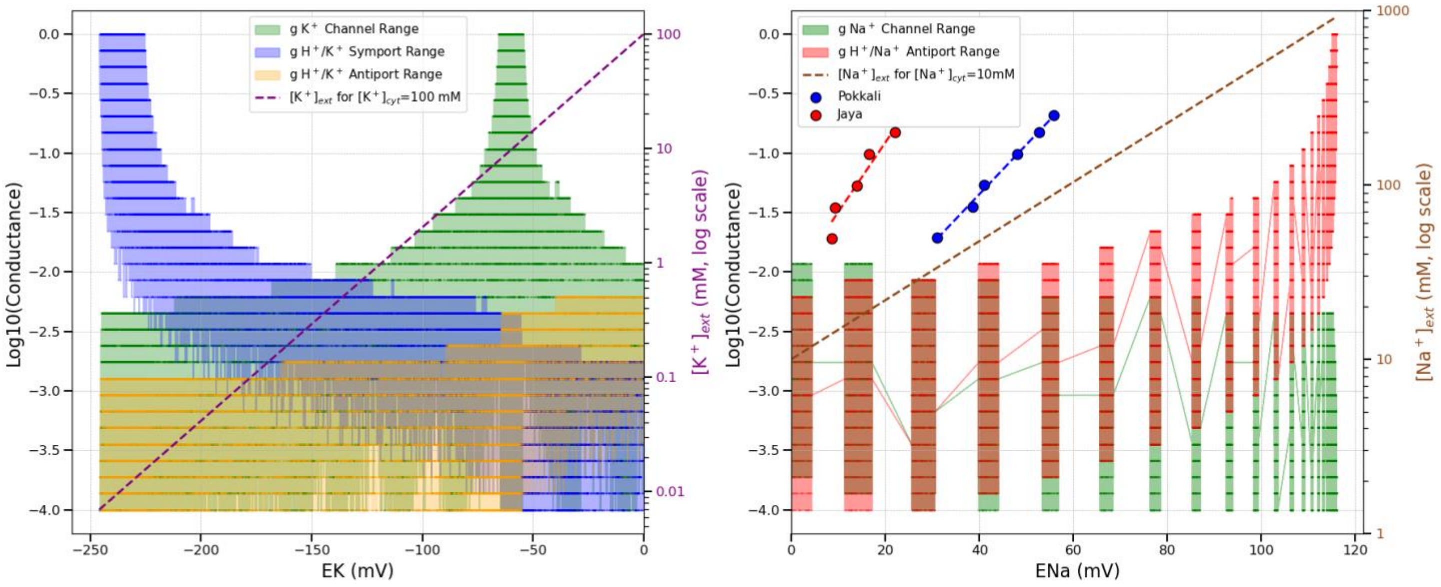

Supplementary Figure S6. Related to Figure 3 (main paper). The effect of simulating PM depolarisation to -60 mV using the code provided above. Note the peak in K^+ channel conductance near the imposed voltage.

2.6 Combining Na^+ and K^+ homeostats on the tonoplast:

Here we consider Na $^+$ and K^+ together, moving in a cycle across the tonoplast at steady-state. The scheme comprises a K^+ antiport (1:1 H^+:K^+), a K^+ channel, a Na^+ antiport (1:1 H^+:Na^+) a Na^+ channel and combined V-ATPase and PP_iase proton pumps.

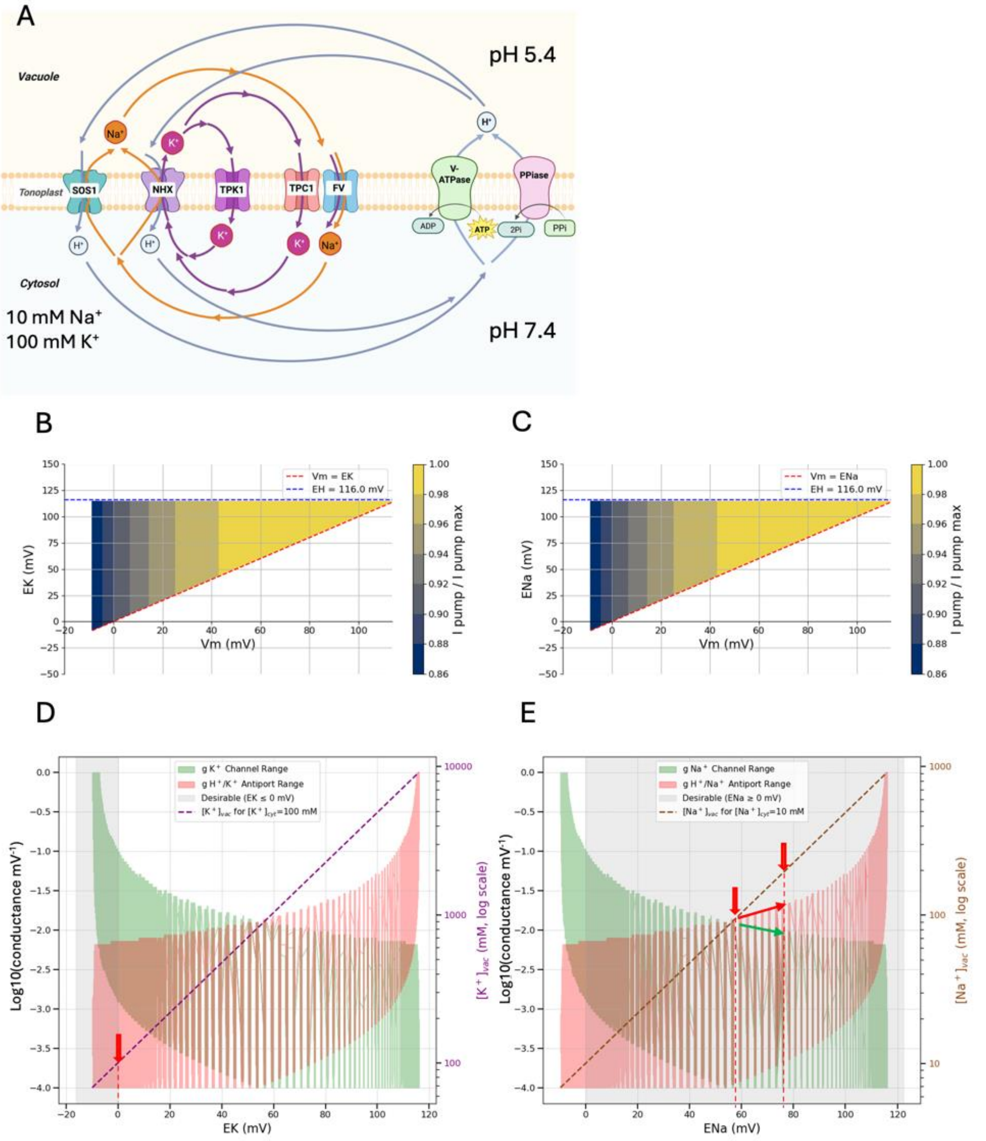

Supplementary Figure S7. (A) The homeostats for K^+ and Na^+ across the tonoplast membrane where Na^+ antiport can be via NHX1,2, which also function as K^+:H^+ antiporters, or via SOS1. The return path for K^+ is via the selective TPK1 or non-selective TPC1 and FV channels. The Na^+ return path is restricted to the non-selective FV channels since TPC1 reportedly prevents Na^+ efflux from the vacuole (Ivashikina and Hedrich, 2005; Jaślan et al., 2019) although it allows Na^+ transport into the vacuole. Two H^+ pumps (V_H^+-ATPase and H^+-PP_iase) provide energy. Note the tonoplast V_m is cytoplasm with respect to the vacuole. (B, C, D, E) The same analysis undertaken for the combined homeostats shown in (A). Here, the constraints on E_K (B) and E_{Na} (C) as a function of V_m reflect the antiport plus channel scenario for both the K^+ and Na^+ homeostats. The less negative V_m is a consequence of the less negative reversal potentials of the tonoplast H^+ pumps. (D) shows the case for perfect $[K^+]_{cyt}$ homeostasis (diagonal dashed line) where $[K^+]_{cyt}$ is maintained at 100 mM and V_m = -10 mV. The shaded area indicates where the $[K^+]_{vac}$ is ≥ 100 mM. (E) As for (D) but where the $[Na^+]_{cyt}$ is kept at 10 mM over a range of $[Na^+]_{vac}$ values. Red vertical arrows indicate E_{Na} for $[Na^+]_{vac}$ at 100 and 200 mM, and horizontal arrows indicate the changes needed in the antiport and channel conductances to achieve steady-state.

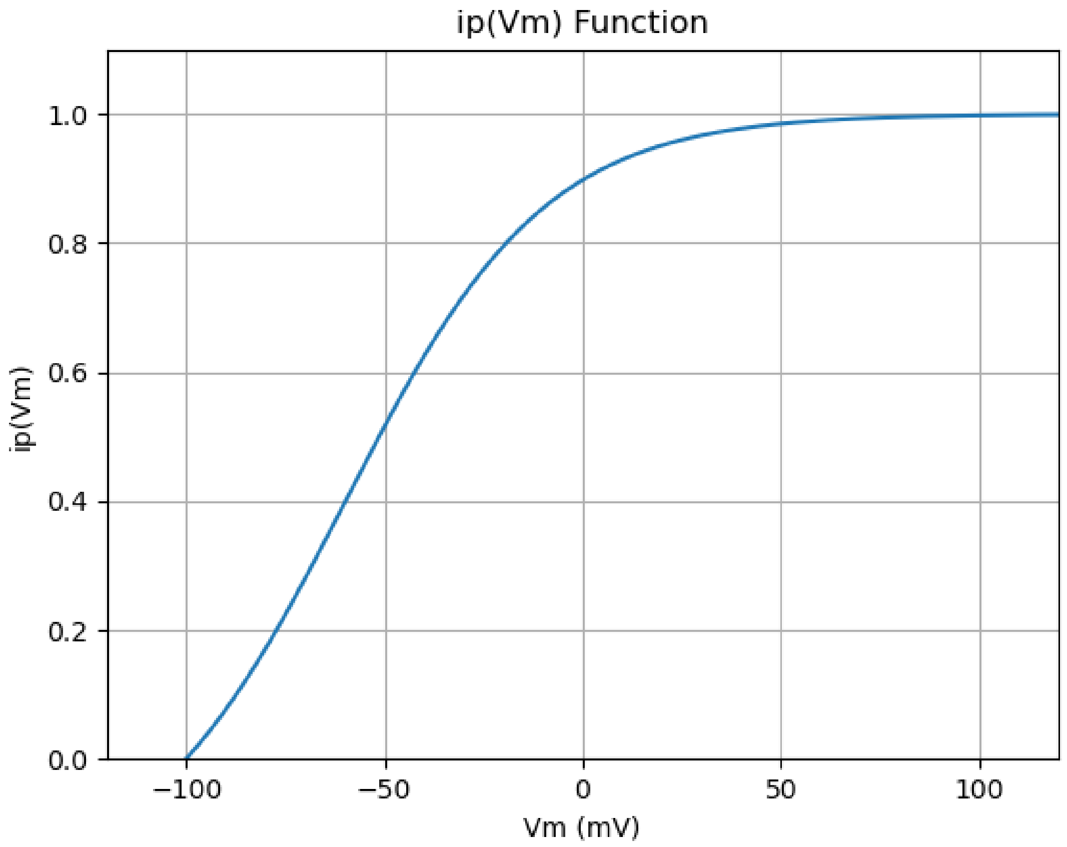

Supplementary Figure S8. Current-voltage curve for combined tonoplast pump function as used in the tonoplast simulation. Based on Li et al. (2024).The equation for the relative pump current is:

$$Ip(rel) = \frac{\left(1 - e^{-0.0396*(V+100)}\right)}{\left(1 + e^{-0.0396*(V+60)}\right)}$$

Supplementary Figure S7 B,C, D, E was generated from steady-state solutions where:

1. **Net K^+ Flux = 0:**

   g_{Kc}*(V_{ss} - E_{Kss}) + g_{HKa}*(E_H - E_{Kss}) = 0

2. **Net Na^+ Flux = 0:**

   g_{Nac}*(V_{ss} - E_{Nass}) + g_{HNaa}*(E_H - E_{Nass}) = 0

3. **Net H^+ Flux = 0:**

   ip(V_{ss}) + g_{HKa}*(E_{Kss} - E_H) + g_{HNaa}*(E_{Nass} - E_H) = 0

- **Net K^+ Flux = 0:** Describes the balance of K^+ fluxes with channels and antiport
- **Net Na^+ Flux = 0:** Describes the balance of Na^+ fluxes with channels and antiport
- **Net H^+ Flux = 0:** Describes the balance of H^+ fluxes

ip(V_{ss}) represents a voltage-dependent H^+ pump, the combined H^+ pumping activity of the tonoplast V-ATPase and PP_iase (Supplementary Figure 7). Conductances are g_{Kc} (K^+ channel), g_{HKa} (H^+:K^+ antiporter 1:1), g_{Nac} (Na^+ channel), g_{HNaa} (H^+:Na^+ antiporter 1:1) all with units mV^{-1}. V_{ss} (steady-state membrane voltage, cytoplasm with respect to the vacuole, mV), E_{Kss} (steady-state K^+ equilibrium potential, mV), E_{Nass} (steady-state Na^+ equilibrium potential, mV), E_H (given H^+ equilibrium potential, mV).

For row 1:

$(g_{Kc})*V_{ss} + (- g_{Kc} - g_{HKa})*E_{Kss} + (0)*E_{Nass} = -g_{HKa}*E_H$

For row 2:

$(g_{NaC})*V_{ss} + (0)*E_{Kss} + (- g_{NaC} - g_{HNaa})*E_{Nass} = - g_{HNaa}*E_H$

For row 3:

$(0)*V_{ss} + (g_{HKa})*E_{Kss} + (g_{HNaa})*E_{Nass} = (g_{HKa} + g_{HNaa})*E_H - ip(V_{ss})$

Matrix:

M=

Row 1: $[(g_{Kc}), (-g_{Kc} - g_{HKa}), 0]$

Row 2: $[(g_{NaC}), 0, (- g_{NaC} - g_{HNaa})]$

Row 3: $[0, (g_{HKa}), (g_{HNaa})]$

Vector of unknowns:

X =[V_{ss}

E_{Kss}

E_{Nass}]

Vector of constants:

A= [$- g_{HKa}*E_H$

$-g_{HNaa}*E_H$

$(g_{HKa} + g_{HNaa})*E_H - ip(V_{ss})$]

Note $ip(V_{ss})$ is the pump current set to max = 1 and solved iteratively for each V_{ss}.

M: is a 3 x 3 matrix of co-efficients derived from the conductances (*g* values) of the different transporters.

X: is a vector containing the unknowns: V_{ss}, E_{Kss}, and E_{Nass}. These represent the steady-state membrane potential, the K^+ Nernst potential, and the Na^+ Nernst potential, respectively.

A: is a vector containing terms related to the driving forces of the ions.

Python Code:

Solve_equation function:

The function takes the conductances (g_{KC}, g_{NaC}, g_{HKa}, g_{HNaa}), the H^+ Nernst potential (E_H), the H^+ pump function (ip_func) combined for V-ATPase and PP_iase), an initial estimate for membrane potential (initial_V_{ss}), a tolerance, and a maximum iteration limit as input.

Takes an iterative approach to solve the system of equations.

The H^+ pump (ip_func) is voltage-dependent and non-linear.

Constructs the matrix M and vector A as described above using numpy.array.

Inside the for loop:

- It updates the ip_V_{ss} variable by calling ip*func(Vss)* to account for the non-linearity of the H^+ pump.

- Uses numpy.linalg.solve(M, A) to solve the linear system of equations. This gives a solution vector X containing updated estimates for V_{ss}, E_{Kss} and E_{Nass}.
- It checks for convergence by comparing the new V_{ss} with the previous one (`abs(Vssnew - Vss) < tolerance). If the difference is less than the tolerance, it considers the solution converged and returns the values.
- If the LinAlgError exception is raised, then it indicates that the equations could not be solved, will return None.
- If the maximum number of iterations is reached without convergence, the function returns None.

H^+ pump Function (ip(V_{ss})):**

- This function defines the voltage-dependent H^+ pump current, ip(V_{ss}).
- The code uses the Nernst equation for the H^+ ion distribution and computes the relative rate of the pump as a function of membrane voltage V_{ss}.
- Uses the combined pump function from Li et al. (2024) (their Equation 2).

Parameter Settings:

The code sets several parameters including:

E_H: the H^+ Nernst potential.

Parameters related to the voltage dependence of the H^+ pumps.

num_points over each *g* value over a log scale. Specifies the number of points to use in the simulation.

Code example (minus plotting) for tonoplast:

```
import numpy as np
import csv
import scipy
import matplotlib.pyplot as plt
from scipy.interpolate import griddata
import matplotlib.tri as tri
from matplotlib import cm
import pandas as pd
from matplotlib.ticker import LogLocator, LogFormatter
print('K + Na, Channel and Antiport, tonoplast pumps, Na Channel independent of K channel conductance')
def solve_equations(gKC, gNaC, gHKa, gHNaa, EH, ip_func, initial_Vss=-20.0, tolerance=1e-12, max_iter=100):
    """
    Iteratively solves the system of equations to determine Vss, EKss, and ENaSS.
    Returns None if the solver fails.
    """
    Vss = initial_Vss
    for i in range(max_iter):
        M = np.array([
            [gKC, -(gKC + gHKa), 0],
            [gNaC, 0, -(gNaC + gHNaa)],
            [0, gHKa, gHNaa]
        ])
```

```
        ip_Vss = ip_func(Vss)
        A = np.array([
            -gHKa * EH,
            -gHNaa * EH,
            (gHKa + gHNaa) * EH - ip_Vss
        ])
        try:
            X = np.linalg.solve(M, A)
            Vss_new, EKss, ENaSS = X
            if abs(Vss_new - Vss) < tolerance:
                return Vss_new, EKss, ENaSS
            Vss = Vss_new
        except np.linalg.LinAlgError:
            return None  # Return None if solver fails
    return None  # Return None if solver fails after max_iter
if __name__ == '__main__':
    # Define parameters
    Vss_target = -10  # Define target Vss value
    Vss_tolerance = 2  # Define the tolerance for Vss matching
    K_in = 100  # Internal K concentration (mM)
    Na_in = 10  # Internal Na concentration for the fourth plot (mM)
    EH = 116

    num_points = 20  #
    g_values = np.logspace(-4, 0, num_points)
# Li et al (2024), but can be set differently see below.
    V0pump = -100
    J3 = 0.0396  # F/RT


    # Define the function ip(Vss)
    def ip(Vss):
        return (1 - np.exp(-J3 * (Vss - V0pump))) / (
                1 + np.exp(-J3 * (Vss + 60)))  # tonoplast pumps after Li et al 2024
    # Lists to store valid results only
    EKss_values = []
    ENaSS_values = []
    Vss_values = []
    ip_values = []
    gKC_values = []
    gNaC_values = []
    gHKa_values = []
    gHNaa_values = []
    filename = "../Modeling membrane fluxes/flux_results.csv"
    header = ["gKC", "gNaC", "gHKa", "gHNaa", "Vss", "EKss", "ENaSS", "JKc", "JNaC", "JKH", "JNaH", "ip"]
```

```
    total_iterations = num_points**4  # Total number of iterations of the nested loop
    print_interval = total_iterations // 10 # print roughly 10 updates
    iteration_count = 0
    with open(filename, mode='w', newline='') as csvfile:
        writer = csv.writer(csvfile)
        writer.writerow(header)
        for gKC_index, gKC in enumerate(g_values):
            for gNaC_index, gNaC in enumerate(g_values):
                for gHNaa_index, gHNaa in enumerate(g_values):
                    for gHKa_index, gHKa in enumerate(g_values):
                        iteration_count += 1
                        result = solve_equations(gKC, gNaC, gHKa, gHNaa, EH, ip)
                        if result:  # Only process if the solver returned a valid result
                            Vss, EKss, ENaSS = result
                            JKc = gKC * (Vss - EKss)
                            JNaC = gNaC * (Vss - ENaSS)
                            JKH = -gHKa * (EKss - EH)
                            JNaH = -gHNaa * (ENaSS - EH)
                            ip_val = ip(Vss)
                            EKss_values.append(EKss)
                            ENaSS_values.append(ENaSS)
                            Vss_values.append(Vss)
                            ip_values.append(ip_val)
                            gKC_values.append(gKC)
                            gNaC_values.append(gNaC)
                            gHKa_values.append(gHKa)
                            gHNaa_values.append(gHNaa)
                            row = [gKC, gNaC, gHKa, gHNaa, Vss, EKss, ENaSS, JKc, JNaC, JKH, JNaH, ip_val]
                            writer.writerow(row)
                        if iteration_count % print_interval == 0:
                            percentage_complete = (iteration_count / total_iterations) * 100
                            print(f"Progress: {percentage_complete:.2f}% complete ({iteration_count}/{total_iterations}
iterations)")
    print(f"Results written to {filename}")
```

Plotting routines not shown.

References

Berkowitz, G. A., & Peters, J. S. (1993). Chloroplast inner-envelope ATPase acts as a primary H^+ Pump. *Plant Physiology*, *102*, 261-267. https://doi.org/10.1104/pp.102.1.261

Bölter, B., & Soll, J. (2001). Ion channels in the outer membranes of chloroplasts and mitochondria: open doors or regulated gates? *EMBO Journal, 20,* 935-940. https://doi.org/10.1093/emboj/20.5.935

Bose, J., Munns, R., Shabala, S., Gilliham, M., Pogson, B., & Tyerman, S. D. (2017). Chloroplast function and ion regulation in plants growing on saline soils: lessons from halophytes. *Journal of Experimental Botany*, *68*, 3129-3143. https://doi.org/10.1093/jxb/erx142

Dreyer, I. (2021). Nutrient cycling is an important mechanism for homeostasis in plant cells. *Plant Physiology*, *187*, 2246-2261. https://doi.org/10.1093/plphys/kiab217

Dreyer, I., Hernández-Rojas, N., Bolua-Hernández, Y., de los Angeles Tapia-Castillo, V., Astola-Mariscal, S. Z., Díaz-Pico, E., Mérida-Quesada, F., Vergara-Valladares, F., Arrey-Salas, O., Rubio-Meléndez, M. E., Riedelsberger, J., & Michard, E. (2024). Homeostats: The hidden rulers of ion homeostasis in plants. *Quantitative Plant Biology*, *5*. https://doi.org/10.1017/qpb.2024.8

Ivashikina, N., & Hedrich, R. (2005). K^+ currents through SV-type vacuolar channels are sensitive to elevated luminal sodium levels. *Plant Journal*, *41*, 606-614. https://doi.org/10.1111/j.1365-313X.2004.02324.x

Jaślan, D., Dreyer, I., Lu, J.P., O'Malley, R., Dindas, J., Marten, I., et al. (2019) Voltage-dependent gating of SV channel TPC1 confers vacuole excitability. *Nature Communications*, *10*, Article 2659. https://doi.org/10.1038/s41467-019-10599-x

Li, K. K., Grauschopf, C., Hedrich, R., Dreyer, I., & Konrad, K. R. (2024). K^+ and pH homeostasis in plant cells is controlled by a synchronized K^+/H^+ antiport at the plasma and vacuolar membrane. *New Phytologist*, *241*, 1525-1542. https://doi.org/10.1111/nph.19436

Tyerman, S. D., Beilby, M., Whittington, J., Juswono, U., Newman, I., & Shabala, S. (2001). Oscillations in proton transport revealed from simultaneous measurements of net current and net proton fluxes from isolated root protoplasts: MIFE meets patch-clamp. *Australian Journal of Plant Physiology*, *28*, 591-604. https://doi.org/10.1071/Pp01030
