# Supplementary material for: Cellular aspects of Na+ homeostasis in plants: Quantitative approaches
Source: Quant Plant Biol. 2026 Mar 2;7:e13. doi: 10.1017/qpb.2026.10040 (PMC13280860; doi:10.1017/qpb.2026.10040)
Supplement: Tyerman et al. supplementary material 2 — Tyerman et al. supplementary material [file S263288282610040Xsup002.pdf]

Supplementary Table S1. Measurements of cytosolic Na^+, K^+ and Cl^- concentration either directly from mature cells or meristematic cells with very small vacuoles and no chloroplasts, or from protoplasts using cytosol-specific SBF1 dye. External NaCl refers to the rooting medium in which the plants were grown, or in the case of protoplasts and cell cultures, the solution in which they were suspended. Square brackets indicate data from the more salt tolerant cultivar or species.

**Species**	**Tissue or cell type**	**$[NaCl]_{ext}$ (mM)**	**Ion concentration (mM)**			**Method of analysis**	**Reference**
			**Na^+**	**K^+**	**Cl^-**		
Barley (2 cvs, salt tolerant, salt sensitive	Root cortical cells (10-20 mm from tip)	200	19 [2]# 19 [28]#	63[59]# 39[59]#	- -	Triple-barrelled H^+, K^+ and Na^+ -selective microelectrodes, also has V_m	Carden et al., 2003
Arabidopsis	Root hairs (5 mM Ca^{2+} added to medium)	30 90	1 15	-	-	Fluorescent dye SPF1 and PBF1	Halperin & Lynch, 2003
Cotton	Root tip (1 mm)	1 150	10 25	80 110	-	Extract*	Zhong & Läuchli, 1994
Atriplex amnicola	Root tip (1 mm)	25 200 400	5 30 50	150 120 100	- -	Extract*	Jeschke et al., 1986
Barley (2 cvs)	Shoot apex (reproductive meristem; 2 - 3 mm)	1 100 175	6 [5] 42 [44] 17 [22]	150 [165] 133 [154] 133 [155]	13 [12] 17 [19] 19 [15]	Extract*	Munns & Rawson, 1999
Wheat (2 cvs)	Shoot apex (reproductive meristem; 2 - 3 mm)	1 100 175	4 [2] 18 [4] 76 [9]	141 [143] 143 [182] 147 [175]	5 [8] 25 [14] 30 [16]	Extract*	Munns & Rawson, 1999
Lettuce	Shoot meristem (10 µm)	10 80	15 25	55 30	15 15	X-ray microanalysis	Lazof & Läuchli, 1991
Rice (2 cvs)	Mesophyll and root protoplasts	5 50 100	2 [4] 7 [13] 15 [25]	- - -	- - -	Fluorescent dye SBF1	Kader & Lindberg, 2005
Rice (2 cvs)	Cells cultured from grain	1 150	2 [5] 25 [80]	-	-	Fluorescent dye SBF1	Anil et al., 2007
Arabidopsis	Mesophyll protoplasts	1 100	2 7	-	-	Fluorescent dye SBF1	Morgan et al., 2022

#Activities (mM), *Hot water extraction of tissues.

Supplementary Table S2. Measurements of [Na^+] (mM) in chloroplasts from plants grown in low or high NaCl. Data are from aqueous extraction of chloroplasts or by X-ray microanalysis of snap-frozen leaves *in situ*. Also shown is the contrast between chloroplasts and the bulk [Na^+] in the leaves from which chloroplasts were isolated. Rhodes grass (*Chloris gayana*) is a C_4 halophyte and *M. crystallinum* is a facultative CAM plant.

**Species**	**Low salt**			**High salt**			**Method**	**References**
	**Added NaCl**	chloroplast	Leaf *(vacuole)*	**External NaCl (mM)**	chloroplast	leaf		
Glycophytes								
Mungbean, 2 cvs	**none**	13	*(8)*	**75**	30	*(10)*	Cryo SEM X-ray	Iqbal et al., 2024
Arabidopsis	**none**	2	2	**150**	4	28	Isolated chloroplasts	Müller et al., 2014
Wheat	**none**	3	7	**-**	-	-	Protoplasts	Robinson & Downton, 1984
Barley	**none**	5	9	**-**	-	-	Protoplasts	*ibid.*
Pea	**none**	41	16	**-**	-	-	Isolated chloroplasts	*ibid.*
Halophyte related								
Sugarbeet	**none**	71	28	**-**	-	-	Isolated chloroplasts	*ibid.*
Spinach	**none**	96	9.5	**200**	165	345	Isolated chloroplasts	Robinson et al., 1983
Spinach	**none**	72	7	**-**			Isolated chloroplasts	Robinson & Downton, 1984
Spinach	**none**	7	2	**300**	22	405	Isolated chloroplasts	Schroppel-Meier & Kaiser, 1988
Halophytes								
Rhodes grass:								
mesophyll	**none**	30	*(34)*	**200**	90	*(307)*	Cryo SEM X-ray	Oi et al., 2022
bundle sheath	**none**	10	*(20)*	**200**	34	*(195)*	Cryo SEM X-ray	*ibid.*
S. australis	**none**	106	10	**350**	115	650	Isolated chloroplasts	Robinson & Downton, 1985
S. maritima	**-**	-	-	**340**	75-93	565	Cryo SEM X-ray	Harvey et al., 1981
S. maritima	**none**	28	-	**340**	84	-	Cryo SEM X-ray	Hajibagheri et al., 1984
M. crystallinum	**20 mM**	99-148	97	**400**	156-234	498	Isolated chloroplasts	Demmig & Winter, 1986
M. crystallinum	**none**	15	-	**400**	110	-	Isolated chloroplasts	Cosentino et al., 2010

Table S3. Na^+ permeating transporters and channels and other transport linked to Na^+ at the plasma membrane and tonoplast.

**Plasma membrane**				
**Gene Name**	**Gene Expression Localisation**	**Effect of high NaCl**	**Na^+ current characteristics**	**Reference**
AtGLR1;1	Roots, stem, petioles. Leaf mesophyll	Downregulated	Low affinity. K^+:Na^+ selectivity = 1:0.8- to 1:0.4 (pore domain expressed in oocytes)	eFP Browser; Roy et al., 2008; Tapken & Hollmann, 2008
AtGLR1;4	Root, stem, petioles. Leaf mesophyll, guard cells	Not affected	Low affinity. Selectivity not reported (oocyte-expressed pore domain)	eFP Browser; Roy et al., 2008; Tapken & Hollmann, 2008
AtGLR3;7	Roots, stem, petioles, leaf epidermis, mesophyll, guard cells	Downregulated	Low affinity (oocytes). Selectivity not reported	eFP Browser; Roy et al., 2008
AtCNGC1	Roots, especially cortical cells & stele .Leaf mesophyll, guard cells	Upregulated	Low affinity. K^+:Na^+ selectivity = 1:1 (HEK293 cells, oocytes)	Balagué et al., 2003; eFP Browser; Hua et al., 2003; Leng et al., 2002; Talke et al., 2003
AtCNGC3	Root: epidermis, cortex, hairs. Leaf: mesophyll, vascular bundle	Transiently upregulated	Low affinity K^+ & Na^+ (yeast). Selectivity not reported	Balagué et al., 2003; eFP Browser; Hua et al., 2003; Leng et al., 2002; Talke et al., 2003
AtCNGC4	Leaf mesophyll cells. Not in roots	Not affected	Low affinity. K^+:Na^+ selectivity = 1:1 (oocytes)	Balagué et al., 2003; eFP Browser; Talke et al., 2003
AtCNGC10	Preferentially in roots. Leaf mesophyll, guard cells	Not affected	Low affinity (HEK293 cells, yeast) . Selectivity not reported	Christopher et al., 2007; eFP Browser; Guo et al., 2008; Talke et al., 2003
AtPIP2;1	Roots, leaves	Downregulated	Low affinity. K^+:Na^+ selectivity = 1:0.5 to 1:0.65 (oocytes)	Boursiac et al., 2005; eFP Browser; Qiu et al., 2025
OsPIP2;4	Root exodermis, endodermis, sclerenchyma, pericycle	Not reported	Low affinity. K^+:Na^+ selectivity = 1:1. K_m = 28 mM (oocytes)	Tran et al., 2025
HvPIP2;8	Roots, shoots, leaves	Root: no change. Shoots: transient increase	Low affinity. K^+:Na^+ selectivity = 1:1. (oocytes)	eFP Browser; Shibasaka et al., 2012; Tran et al., 2020
AtHKT1;1	Root & leaf xylem parenchyma & phloem. Root epidermis, cortex.	Expression peaks at 30 mM Na^+ or expression unaffected by Na^+	Na^+ selective (oocytes). Regulates vascular [Na^+]. Contributes to root Na^+ uptake (*in planta*)	Berthomieu et al., 2003; Rus et al., 2001; Sunarpi et al., 2005; Wang et al., 2015; Zhang et al., 2008
OsHKT1;1	Root epidermis, exodermis, cortex, stele (mainly phloem). Leaf bulliform cells, vascular tissues	Roots: upregulated. Shoots: downregulated	Na^+ selective. K_m = 20 µM & 1 mM (yeast, oocytes)	Garciadeblás et al., 2003; Horie et al., 2001; Imran et al., 2020; Jabnoune et al., 2009; Khan et al., 2020; Wang et al., 2015

HvHKT1;1	Roots, stele, parenchyma cells, epidermis, senescent leaves	Upregulated	Na^+ selective. Inhibited by high $[K^+]_{ext}$ (yeast, oocytes)	Han et al., 2018; Tian et al., 2024; Zhu et al., 2024
OsHKT1;3	Root cortex, stele, vasculature. Leaf vasculature, bulliform & mesophyll. On Golgi membrane	Upregulated	Na^+ selective. K_m = 3.5 mM (oocytes)	Jabnoune et al., 2009; Khan et al., 2020; Kobayashi et al., 2017; Rosas-Santiago et al., 2015
HvHKT1;3	Roots, leaves. Golgi membrane	Shoot: downregulated. Root: upregulated	Na^+ selective (oocytes)	Rosas-Santiago et al., 2015; Tian et al., 2024
OsHTK1;4	Leaf sheaths	Upregulated	Na^+ selective (oocytes)	Cotsaftis et al., 2012; Khan et al., 2020; Suzuki et al., 2016
HvHKT1;4	Leaf epidermis	Up & downregulation reported	Na^+ selective (oocytes)	Tian et al., 2024; Zhu et al., 2024
TmHKT1;4-A1	Roots, leaves	Roots: weak affect. Leaves: upregulated	Na^+ selective. High $[K^+]_{ext}$ enhances Na^+ current. K_m = 3 mM (oocytes)	Ben Amar et al., 2014; Tounsi et al., 2021
TmHKT1;4-A2 Nax1	Roots. Leaf sheaths, vascular tissue	Weak affect	Na^+ selective. Na^+ current enhanced by high $[K^+]_{ext}$. K_m = 12 mM (oocytes)	Ben Amar et al., 2014; Huang et al., 2006; James et al., 2011; Munns et al., 2012; Tounsi et al., 2016, 2021
OsHKT1;5	Root & shoot parenchyma	Upregulated	Na^+ selective (oocytes)	Cotsaftis et al., 2012; Garciadeblás et al., 2003; Khan et al., 2020; Kobayashi et al., 2017; Ren et al., 2005
HvHKT1;5	Root epidermis, parenchyma, pericycle, stele	Roots: upregulated. Shoots: downregulated	Na^+ selective. Inhibited by high $[K^+]_{ext}$ (oocytes)	Huang et al., 2020; Tian et al., 2024; Zhu et al., 2024
TmHKT1;5-A Nax2	Roots, parenchyma, pericycle	Not affected	Na^+ selective. Dual affinity: $[Na^+]_{ext}$ < 100 µM, K_m = 18 µM, $[Na^+]_{ext}$ > 100 µM, K_m = 1 mM. High affinity abolished when $[K^+]_{ext}$ > $[Na^+]_{ext}$. (oocytes)	Byrt et al., 2014; James et al., 2011; Munns et al., 2012; Xu et al., 2018, 2020
TaHKT1;5-D Kna1	Roots, xylem parenchyma, pericycle	Not affected	Na^+ selective. Dual affinity: $[Na^+]_{ext}$ < 100 µM, K_m = 15 µM. $[Na^+]_{ext}$ > 100 µM, K_m = 4 mM. High affinity abolished when $[K^+]_{ext}$ > $[Na^+]_{ext}$. (oocytes)	Byrt et al., 2014; Xu et al., 2018, 2020
OsHKT2;1	Root: epidermis, endodermis, exodermis, cortex, stele (mainly phloem). Leaf: vascular tissues, bulliform & mesophyll cells	Downregulated in roots	High affinity Na^+-K^+ co-transport, low affinity Na^+ uniport. K_m = 9.5 µM & 2.2 mM (oocytes), 11 mM (yeast) 14 µM (tobacco BY2 cells)	Garciadeblás et al., 2003; Haro et al., 2005; Horie et al., 2001, 2007; Jabnoune et al., 2009; Malagoli et al., 2008; Oomen et al., 2012; Yao et al., 2010

HvHKT2;1 Eight isoforms	Root cortex. Leaf blades, sheaths	Downregulated in leaf sheaths & roots. Upregulated in leaf blades	Low $[Na^+]_{ext}$: high affinity Na^+-K^+ co-transport. High $[Na^+]_{ext}$: low affinity Na^+ transport. K_m = 7 mM (oocytes)	Haro et al., 2005; Mian et al., 2011; Tian et al., 2024; Wang et al., 1998; Zhu et al., 2024
OsHKT2;2	Roots	Low Na^+: upregulated. High Na^+: downregulated	Low $[Na^+]_{ext}$: Na^+-K^+ co-transport (1:1) (oocytes, tobacco BY2 cells). High $[Na^+]_{ext}$: Na^+-selective (oocytes, BY2 cells). K_m = 77 µM (BY2 cells)	Horie et al., 2001; Jabnoune et al., 2009; Oomen et al., 2012; Yao et al., 2010
OsHKT2;2/1 (pseudo-gene)	Roots	Decreased expression	High & low affinity Na^+-K^+ co-transport (oocytes, yeast)	Oomen et al., 2012
HvHKT2;2	Roots & shoot, low expression	Roots: downregulated. Leaf: up & downregulation reported	High affinity Na^+-K^+ co-transport, low affinity Na^+ uniport (assumed from structure)	Tian et al., 2024; Zhu et al., 2024
TaHKT2;1 (at least six isoforms)	Root cortical cells. Leaf vasculature, sheaths	Upregulated in roots & leaf sheaths. Downregulated in leaf blades	High affinity Na^+-K^+ co-transport at low $[Na^+]_{ext}$. Low affinity Na^+ transport at high $[Na^+]_{ext}$. Inhibited by high $[K^+]_{ext}$ (oocytes)	Almeida et al., 2013; Ariyarathna et al., 2014; Gassmann et al., 1996; Laurie et al., 2002; Riedelsberger et al., 2021; Rubio et al., 1995; Schachtman & Schroeder, 1994
OsHKT2;3	Roots, leaf blade & sheath	Not reported	High affinity Na^+-K^+ co-transport, low affinity Na^+ uniport (assumed from structure)	Hamamoto et al., 2015
OsHKT2;4	Root vasculature, hairs. Leaf blade, sheath, epidermis	Not reported	High affinity Na^+-K^+ co-transport, low affinity Na^+ uniport (yeast, oocytes)	Horie et al., 2011; Lan et al., 2010
TaLCT1	Roots, leaves	Upregulated in roots	Low affinity. Na^+ & K^+ transport. Selectivity: $Na^+ > Rb^+$ (yeast)	Amtmann et al., 2001; Schachtman et al., 1997
HvHAK1	Roots	Not reported	Low affinity Na^+ uptake in yeast	Santa-María et al., 1997
OsHAK2	Ubiquitous	Not reported	Na^+ selective in *E. coli*. Oocytes: K^+ & Na^+ transport not detected	Gupta et al., 2008; Horie et al., 2011; Morita et al., 2023; Okada et al., 2018
OsHAK4	Vegetative stage: minimal expression. Reproductive stage: high expression in phloem, peduncle, rachis	Not affected	Na^+/H^+ symport. Na^+ selective. Dual affinity: $[Na^+]_{ext}$ < 5 mM, K_m = 180 µM. $[Na^+]_{ext}$ > 10 mM, linear current response (oocytes)	Che et al., 2024
OsHAK12	Root vasculature, hairs, exodermis, cortex, endodermis. Shoot: stems, leaf mesophyll	Upregulated	Na^+-selective (yeast)	Zhang et al., 2021

OsHAK18	Root & shoot vasculature	Not reported	Uncertain. K^+ & Na^+ transport or K^+ selective. Does not transport Na^+ in yeast or have a Na^+ binding site	Peng et al., 2023; Saha et al., 2023; Shen et al., 2023
AtNRT1.1	Root: epidermis, conductive tissues. Leaf: mesophyll, guard cells	Downregulated	Low affinity. $[NO_3^-]_{ext}$-dependent Na^+ transport (*in planta*)	Alvarez-Aragón & Rodríguez-Navarro, 2017; Liu et al., 2020
AtNRT1.2	Root: epidermis, cortex, hairs. Leaf: mesophyll, guard cells	Root: no affect. Shoots: phosphorylated & downregulated by SOS2	Low affinity. $[NO_3^-]_{ext}$-dependent Na^+ transport (oocytes)	Huang et al., 1999; Liu et al., 2025b; Okamoto et al., 2003
AtCCC1	Roots: epidermis, pericycle, parenchyma. Shoots: stem, leaves, trichomes, hydathodes	Not affected	High affinity. Stoichiometry: 1 Na^+:1 K^+: 2 Cl^- or 1 K^+:1 Cl^- (oocytes)	Colmenero-Flores et al., 2007; eFP Browser; Henderson et al., 2015
OsCCC1	Roots, leaf, basal node	Not affected	High affinity. Stoichiometry: 1 Na^+:1 K^+: 2 Cl^- or 1 K^+:1 Cl^- (yeast)	Chen et al., 2016
AtNHX7 (AtSOS1)	Entire root. Leaf mesophyll, guard cells, parenchyma. More abundant in roots than shoots	Upregulated. Phosphorylated & activated by CBL4-CIPK24 (AtSOS3-AtSOS2)	Na^+ selective. K_m = 23 mM (PM vesicles)	eFP Browser; Qiu et al., 2002, 2003; Quintero et al., 2002; Ramakrishna et al., 2025; Shi et al., 2000, 2002, 2003
OsSOS1	Roots, leaves	Upregulated. Phosphorylated & activated by OsCIPK24 & OsCBL4	Na^+ selective. K_m = 29 mM (PM vesicles)	Martínez-Atienza et al., 2007
**Tonoplast**				
**Gene Name**	**Gene Expression Localisation**	**Expression under high NaCl**	**Na^+ current characteristics in heterologous systems**	**Reference**
AtNHX1	Entire root, especially vasculature. Leaf: epidermis, mesophyll, especially guard cells & vasculature. Stems, cortex	Upregulated	K^+:Na^+ selectivity: various. 1:1 (yeast liposomes) 1:0.78, 1:0.61, 1:3, 1:1.8 (isolated leaf vacuoles). K_m = 6 to 30 mM (isolated leaf vacuoles)	Apse et al., 1999, 2003; Bassil et al., 2011, 2019; Venema et al., 2002; Yamaguchi et al., 2003, 2005; Yokoi et al., 2002
AtNHX2	Entire root, especially stele. Leaf, especially guard cells. Stems	Upregulated	K^+:Na^+ selectivity = 1:1 (yeast liposomes). K_m = 18 mM (isolated leaf vacuoles)	Bassil et al., 2011, 2019; Venema et al., 2002; Yokoi et al., 2002
AtNHX3	Predominantly in roots. Leaf, especially guard cells. Stems	No response & upregulation reported	Na^+ selective. K_m = 15 mM (isolated leaf vacuoles)	Bassil et al., 2019; Liu et al., 2010; Yokoi et al., 2002
AtNHX4	Root, leaf, stems, guard cells	Upregulated in root	K^+ and Na^+ selectivity not reported. K_m = 16 mM (isolated leaf vacuoles)	Bassil et al., 2019; Li et al., 2009; Yokoi et al., 2002

AtNHX7 (AtSOS1)	As above	Increases at or redirected to tonoplast	As above	Liu et al., 2025a; Ramakrishna et al., 2025
AtTPC1	Root. Leaf mesophyll, guard cells. Stems	Shoots: transiently upregulated. Roots: no response	K^+:Na^+ selectivity = 1:1 (mesophyll vacuoles) Unidirectional to vacuole	Furuichi et al., 2001; Ivashikina & Hedrich, 2005; Jaślan et al., 2019; Ranf et al., 2008
AtTPK1	Roots. Leaf: mesophyll, guard cells, vasculature	No response at transcriptional level. Activated by CPK-mediated phosphorylation & 14-3-3s	K^+ only (mesophyll vacuoles)	Czempinski et al., 2002; Gobert et al., 2007; Latz et al., 2007, 2013; Schönknecht et al., 2002
AtNCL	Ubiquitously expressed	Upregulated	Na^+(K^+)/Ca^{2+} exchange. Affinity: Na^+ > K^+. Vacuolar Ca^{2+} sequestration & Na^+ release (mammalian CHO-K1 cells) or Na^+ vacuolar sequestration & Ca^{2+} release (yeast).	eFP Browser; Li et al., 2016; Wang et al., 2012

References

Almeida, P., Katschnig, D., & de Boer, A. H. (2013). HKT Transporters - state of the art. *International Journal of Molecular Sciences, 14*, 20359-20385. https://doi.org/10.3390/ijms141020359

Alvarez-Aragón, R., & Rodríguez-Navarro, A. (2017). Nitrate-dependent shoot sodium accumulation and osmotic functions of sodium in Arabidopsis under saline conditions. *Plant Journal*, *91*, 208-219. https://doi.org/10.1111/tpj.13556

Amtmann, A., Fischer, M., Marsh, E. L., Stefanovic, A., Sanders, D., & Schachtman, D. P. (2001). The wheat cDNA generates hypersensitivity to sodium in a salt-sensitive yeast strain. *Plant Physiology, 126,* 1061-1071. https://doi.org/10.1104/pp.126.3.1061

Anil, V. S., Krishnamurthy, H., & Mathew, M. K. (2007). Limiting cytosolic Na^+ confers salt tolerance to rice cells in culture: a two-photon microscopy study of SBFI-loaded cells. *Physiologia Plantarum*, *129*, 607-621. https://doi.org/10.1111/j.1399-3054.2006.00854.x

Apse, M. P., Aharon, G. S., Snedden, W. A., & Blumwald, E. (1999). Salt tolerance conferred by overexpression of a vacuolar Na^+/H^+ antiport in Arabidopsis. *Science*, *285*, 1256-1258. https://doi.org/10.1126/science.285.5431.1256

Apse, M. P., Sottosanto, J. B., & Blumwald, E. (2003). Vacuolar cation/H^+ exchange, ion homeostasis, and leaf development are altered in a T-DNA insertional mutant of AtNHX1, the Arabidopsis vacuolar Na^+/H^+ antiporter. *Plant Journal*, *36*, 229-239. https://doi.org/10.1046/j.1365-313X.2003.01871.x

Ariyarathna, H., Ul-Haq, T., Colmer, T. D., & Francki, M. G. (2014). Characterization of the multigene family TaHKT 2;1 in bread wheat and the role of gene members in plant Na^+ and K^+ status. *BMC Plant Biology, 14,* Article 159. https://doi.org/10.1186/1471-2229-14-159

Balagué, C., Lin, B. Q., Alcon, C., Flottes, G., Malmström, S., Köhler, C., Neuhaus, G., Pelletier, G., Gaymard, F., & Roby, D. (2003). HLM1, an essential signaling component in the hypersensitive response, is a member of the cyclic nucleotide-gated channel ion channel family. *Plant Cell*, *15*, 365-379. https://doi.org/10.1105/tpc.006999

Bassil, E., Tajima, H., Liang, Y. C., Ohto, M., Ushijima, K., Nakano, R., Esumi, T., Coku, A., Belmonte, M., & Blumwald, E. (2011). The Arabidopsis Na^+/H^+ antiporters NHX1 and NHX2 control vacuolar pH and K^+ homeostasis to regulate growth, flower development, and reproduction. *Plant Cell*, *23*, 3482-3497. https://doi.org/10.1105/tpc.111.089581

Bassil, E., Zhang, S. Q., Gong, H. J., Tajima, H., & Blumwald, E. (2019). Cation specificity of vacuolar NHX-Type cation/H^+ antiporters. *Plant Physiology*, *179*, 616-629. https://doi.org/10.1104/pp.18.01103

Ben Amar, S., Brini, F., Sentenac, H., Masmoudi, K., & Véry, A. A. (2014). Functional characterization in *Xenopus* oocytes of Na^+ transport systems from durum wheat reveals diversity among two HKT1;4 transporters. *Journal of Experimental Botany, 65,* 213-222. https://doi.org/10.1093/jxb/ert361

Berthomieu, P., Conéjéro, G., Nublat, A., Brackenbury, W. J., Lambert, C., Savio, C., Uozumi, N., Oiki, S., Yamada, K., Cellier, F., Gosti, F., Simonneau, T., Essah, P. A., Tester, M., Véry, A. A., Sentenac, H., & Casse, F. (2003). Functional analysis of AtHKT1 in Arabidopsis shows that Na^+ recirculation by the phloem is crucial for salt tolerance. *EMBO Journal*, *22*, 2004-2014. https://doi.org/10.1093/emboj/cdg207

Boursiac, Y., Chen, S., Luu, D. T., Sorieul, M., van den Dries, N., & Maurel, C. (2005). Early effects of salinity on water transport in Arabidopsis roots. Molecular and cellular features of aquaporin expression. *Plant Physiology*, *139*, 790-805. https://doi.org/10.1104/pp.105.065029

Byrt, C. S., Xu, B., Krishnan, M., Lightfoot, D. J., Athman, A., Jacobs, A. K., Watson-Haigh, N. S., Plett, D., Munns, R., Tester, M., & Gilliham, M. (2014). The Na^+ transporter, TaHKT1;5-D, limits shoot Na^+ accumulation in bread wheat. *Plant Journal, 80,* 516-526. https://doi.org/10.1111/tpj.12651

Carden, D. E., Walker, D. J., Flowers, T. J., & Miller, A. J. (2003). Single-cell measurements of the contributions of cytosolic Na^+ and K^+ to salt tolerance. *Plant Physiology*, *131*, 676-683. https://doi.org/10.1104/pp.011445

Che, J., Yamaji, N., Wang, S. F., Xia, Y., Yang, S. Y., Su, Y. H., Shen, R. F., & Ma, J. F. (2024). OsHAK4 functions in retrieving sodium from the phloem at the reproductive stage of rice. *Plant Journal, 120,* 76-90. https://doi.org/10.1111/tpj.16971

Chen, Z. C., Yamaji, N., Fujii-Kashino, M., & Ma, J. F. (2016). A cation-chloride cotransporter gene is required for cell elongation and osmoregulation in rice. *Plant Physiology, 171,* 494-507. https://doi.org/10.1104/pp.16.00017

Christopher, D. A., Borsics, T., Yuen, C. Y. L., Ullmer, W., Andème-Ondzighi, C., Andres, M. A., Kang, B. H., & Staehelin, L. A. (2007). The cyclic nucleotide gated cation channel AtCNGC10 traffics from the ER via Golgi vesicles to the plasma membrane of Arabidopsis root and leaf cells. *BMC Plant Biology*, *7*, Article 48. https://doi.org/10.1186/1471-2229-7-48

Colmenero-Flores, J. M., Martínez, G., Gamba, G., Vázquez, N., Iglesias, D. J., Brumós, J., & Talón, M. (2007). Identification and functional characterization of cation-chloride cotransporters in plants. *Plant Journal*, *50*, 278-292. https://doi.org/10.1111/j.1365-313X.2007.03048.x

Cosentino, C., Fischer-Schliebs, E., Bertl, A., Thiel, G., & Homann, U. (2010). Na^+/H^+ antiporters are differentially regulated in response to NaCl stress in leaves and roots

of *Mesembryanthemum crystallinum*. *New Phytologist*, *186*, 669-680. https://doi.org/10.1111/j.1469-8137.2010.03208.x

Cotsaftis, O., Plett, D., Shirley, N., Tester, M., & Hrmova, M. (2012). A two-staged model of Na^+ exclusion in rice explained by 3D modeling of HKT transporters and alternative splicing. *PLOS One, 7*. https://doi.org/10.1371/journal.pone.0039865

Czempinski, K., Frachisse, J. M., Maurel, C., Barbier-Brygoo, H., & Mueller-Roeber, B. (2002). Vacuolar membrane localization of the 'two-pore' K^+ channel KCO1. *Plant Journal, 29,* 809-820. https://doi.org/10.1046/j.1365-313X.2002.01260.x

Demmig, B., & Winter, K. (1986). Sodium, potassium, chloride and proline concentrations of chloroplasts isolated from a halophyte, *Mesembryanthemum crystallinum* L. *Planta*, *168*(3), 421-426. https://doi.org/10.1007/Bf00392371

eFP Browser: *Arabidopsis eFP Browser*. https://bar.utoronto.ca/efp/cgi-bin/efpWeb.cgi

Furuichi, T., Cunningham, K. W., & Muto, S. (2001). A putative two pore channel AtTPC1 mediates Ca^{2+} flux in Arabidopsis leaf cells. *Plant and Cell Physiology*, *42*, 900-905. https://doi.org/10.1093/pcp/pce145

Garciadeblás, B., Senn, M. E., Bañuelos, M. A., & Rodríguez-Navarro, A. (2003). Sodium transport and HKT transporters: the rice model. *Plant Journal, 34,* 788-801. https://doi.org/10.1046/j.1365-313X.2003.01764.x

Gassmann, W., Rubio, F., & Schroeder, J. I. (1996). Alkali cation selectivity of the wheat root high-affinity potassium transporter HKT1. *Plant Journal, 10,* 869-882. https://doi.org/10.1046/j.1365-313X.1996.10050869.x

Gobert, A., Isayenkov, S., Voelker, C., Czempinski, K., & Maathuis, F. J. M. (2007). The two-pore channel TPK1 gene encodes the vacuolar K^+ conductance and plays a role in K^+ homeostasis. *Proceedings of the National Academy of Sciences of the United States of America, 104,* 10726-10731. https://doi.org/10.1073/pnas.0702595104

Guo, K. M., Babourina, O., Christopher, D. A., Borsics, T., & Rengel, Z. (2008). The cyclic nucleotide-gated channel, AtCNGC10, influences salt tolerance in Arabidopsis. *Physiologia Plantarum*, *134*, 499-507. https://doi.org/10.1111/j.1399-3054.2008.01157.x

Gupta, M., Qiu, X. H., Wang, L., Xie, W. B., Zhang, C. J., Xiong, L. Z., Lian, X. M., & Zhang, Q. F. (2008). KT/HAK/KUP potassium transporters gene family and their whole-life cycle expression profile in rice (*Oryza sativa*). *Molecular Genetics and Genomics, 280,* 437-452. https://doi.org/10.1007/s00438-008-0377-7

Hajibagheri, M. A., Harvey, D. M. R., & Flowers, T. J. (1984). Photosynthetic oxygen evolution in relation to ion contents in the chloroplasts of *Suaeda maritima*. *Plant Science Letters*, *34*, 353-362. https://doi.org/10.1016/S0304-4211(84)80015-2

Hamamoto, S., Horie, T., Hauser, F., Deinlein, U., Schroeder, J. I., & Uozumi, N. (2015). HKT transporters mediate salt stress resistance in plants: from structure and function to the field. *Current Opinion in Biotechnology, 32*, 113-120. https://doi.org/10.1016/j.copbio.2014.11.025

Halperin, S. J., & Lynch, J. P. (2003). Effects of salinity on cytosolic Na^+ and K^+ in root hairs of: measurements using the fluorescent dyes SBFI and PBFI. *Journal of Experimental Botany*, *54*, 2035-2043. https://doi.org/10.1093/jxb/erg219

Han, Y., Yin, S. Y., Huang, L., Wu, X. L., Zeng, J. B., Liu, X. H., Qiu, L., Munns, R., Chen, Z. H., & Zhang, G. P. (2018). A sodium transporter HvHKT1;1 confers salt tolerance in barley via regulating tissue and cell ion homeostasis. *Plant and Cell Physiology, 59,* 1976-1989. https://doi.org/10.1093/pcp/pcy116

Haro, R., Bañuelos, M. A., Senn, M. A. E., Barrero-Gil, J., & Rodríguez-Navarro, A. (2005). HKT1 mediates sodium uniport in roots. Pitfalls in the expression of HKT1 in yeast. *Plant Physiology, 139,* 1495-1506. https://doi.org/10.1104/pp.105.067553

Harvey, D. M. R., Hall, J. L., Flowers, T. J., & Kent, B. (1981). Quantitative ion localization within *Suaeda maritima* leaf mesophyll cells. *Planta*, *151*, 555-560. https://doi.org/10.1007/Bf00387435

Henderson, S. W., Wege, S., Qiu, J., Blackmore, D. H., Walker, A. R., Tyerman, S. D., Walker, R. R., & Gilliham, M. (2015). Grapevine and Arabidopsis cation-chloride cotransporters localize to the golgi and trans-golgi network and indirectly influence long-distance ion transport and plant salt tolerance. *Plant Physiology*, *169*, 2215-2229. https://doi.org/10.1104/pp.15.00499

Horie, T., Costa, A., Kim, T. H., Han, M. J., Horie, R., Leung, H. Y., Miyao, A., Hirochika, H., An, G., & Schroeder, J. I. (2007). Rice OsHKT2;1 transporter mediates large Na^+ influx component into K^+-starved roots for growth. *EMBO Journal, 26,* 3003-3014. https://doi.org/10.1038/sj.emboj.7601732

Horie, T., Sugawara, M., Okada, T., Taira, K., Kaothien-Nakayama, P., Katsuhara, M., Shinmyo, A., & Nakayama, H. (2011). Rice sodium-insensitive potassium transporter, OsHAK5, confers increased salt tolerance in tobacco BY2 cells. *Journal of Bioscience and Bioengineering, 111,* 346-356. https://doi.org/10.1016/j.jbiosc.2010.10.014

Horie, T., Yoshida, K., Nakayama, H., Yamada, K., Oiki, S., & Shinmyo, A. (2001). Two types of HKT transporters with different properties of Na^+ and K^+ transport in *Oryza sativa*. *Plant Journal, 27,* 129-138. https://doi.org/10.1046/j.1365-313x.2001.01077.x

Hua, B. G., Mercier, R. W., Leng, Q., & Berkowitz, G. A. (2003). Plants do it differently. A new basis for potassium/sodium selectivity in the pore of an ion channel. *Plant Physiology*, *132*, 1353-1361. https://doi.org/10.1104/pp.103.020560

Huang, N. C., Liu, K. H., Lo, H. J., & Tsay, Y. F. (1999). Cloning and functional characterization of an Arabidopsis nitrate transporter gene that encodes a constitutive component of low-affinity uptake. *Plant Cell*, *11*, 1381-1392. https://doi.org/10.1105/tpc.11.8.1381

Huang, L., Kuang, L. H., Wu, L. Y., Shen, Q. F., Han, Y., Jiang, L. X., Wu, D. Z., & Zhang, G. P. (2020). The HKT transporter HvHKT1;5 negatively regulates salt tolerance. *Plant Physiology, 182,* 584-596. https://doi.org/10.1104/pp.19.00882

Huang, S. B., Spielmeyer, W., Lagudah, E. S., James, R. A., Platten, J. D., Dennis, E. S., & Munns, R. (2006). A sodium transporter (HKT7) is a candidate for *Nax1*, a gene for salt tolerance in durum wheat. *Plant Physiology, 142,* 1718-1727. https://doi.org/10.1104/pp.106.088864

Imran, S., Horie, T., & Katsuhara, M. (2020). Expression and ion transport activity of rice variants. *Plants-Basel, 9*. https://doi.org/10.3390/plants9010016

Iqbal, M. S., Clode, P. L., Malik, A., Erskine, W., & Kotula, L. (2024). Salt tolerance in mungbean is associated with controlling Na and Cl transport across roots, regulating Na and Cl accumulation in chloroplasts and maintaining high K in root and leaf mesophyll cells. *Plant Cell and Environment*, *47*, 3638-3653. https://doi.org/10.1111/pce.14943

Ivashikina, N., & Hedrich, R. (2005). K^+ currents through SV-type vacuolar channels are sensitive to elevated luminal sodium levels. *Plant Journal*, *41*, 606-614. https://doi.org/10.1111/j.1365-313X.2004.02324.x

Jabnoune, M., Espeout, S., Mieulet, D., Fizames, C., Verdeil, J. L., Conéjéro, G., Rodríguez-Navarro, A., Sentenac, H., Guiderdoni, E., Abdelly, C., & Véry, A. A. (2009). Diversity in expression patterns and functional properties in the rice HKT Transporter family. *Plant Physiology, 150,* 1955-1971. https://doi.org/10.1104/pp.109.138008

James, R. A., Blake, C., Byrt, C. S., & Munns, R. (2011). Major genes for Na^+ exclusion, Nax1 and Nax2 (wheat HKT1;4 and HKT1;5), decrease Na^+ accumulation in bread wheat leaves under saline and waterlogged conditions. *Journal of Experimental Botany, 62,* 2939-2947. https://doi.org/10.1093/jxb/err003

Jaślan, D., Dreyer, I., Lu, J.P., O'Malley, R., Dindas, J., Marten, I., et al. (2019). Voltage-dependent gating of SV channel TPC1 confers vacuole excitability. *Nature Communications*, *10*, Article 2659. https://doi.org/10.1038/s41467-019-10599-x

Jeschke, W. D., Aslam, Z., & Greenway, H. (1986). Effects of NaCl on ion relations and carbohydrate status of roots and on osmotic regulation of roots and shoots of *Atriplex amnicola*. *Plant Cell and Environment*, *9*, 559-569. https://doi.org/10.1111/j.1365-3040.1986.tb01587.x

Kader, M. A., & Lindberg, S. (2005). Uptake of sodium in protoplasts of salt-sensitive and salt-tolerant cultivars of rice, *Oryza sativa* L. determined by the fluorescent dye SBFI. *Journal of Experimental Botany*, *56*, 3149-3158. https://doi.org/10.1093/jxb/eri312

Khan, I., Mohamed, S., Regnault, T., Mieulet, D., Guiderdoni, E., Sentenac, H., & Véry, A. A. (2020). Constitutive contribution by the rice OsHKT1;4 Na^+ transporter to xylem sap desalinization and low Na^+ accumulation in young leaves under low as high external Na^+ conditions. *Frontiers in Plant Science, 11,* 1130. https://doi.org/10.3389/fpls.2020.01130

Kobayashi, N. I., Yamaji, N., Yamamoto, H., Okubo, K., Ueno, H., Costa, A., Tanoi, K., Matsumura, H., Fujii-Kashino, M., Horiuchi, T., Al Nayef, M., Shabala, S., An, G., Ma, J. F., & Horie, T. (2017). OsHKT1;5 mediates Na^+ exclusion in the vasculature to protect leaf blades and reproductive tissues from salt toxicity in rice. *Plant Journal, 91,* 657-670. https://doi.org/10.1111/tpj.13595

Lan, W. Z., Wang, W., Wang, S. M., Li, L. G., Buchanan, B. B., Lin, H. X., Gao, J. P., & Luan, S. (2010). A rice high-affinity potassium transporter (HKT) conceals a calcium-permeable cation channel. *Proceedings of the National Academy of Sciences of the United States of America, 107,* 7089-7094. https://doi.org/10.1073/pnas.1000698107

Latz, A., Becker, D., Hekman, M., Müeller, T., Beyhl, D., Marten, I., Eing, C., Fischer, A., Dunkel, M., Bertl, A., Rapp, U. R., & Hedrich, R. (2007). TPK1, a Ca^{2+}-regulated Arabidopsis vacuole two-pore K^+ channel is activated by 14-3-3 proteins. *Plant Journal, 52,* 449-459. https://doi.org/10.1111/j.1365-313X.2007.03255.x

Latz, A., Mehlmer, N., Zapf, S., Mueller, T. D., Wurzinger, B., Pfister, B., Csaszar, E., Hedrich, R., Teige, M., & Becker, D. (2013). Salt stress triggers phosphorylation of the vacuolar K^+ channel TPK1 by calcium-dependent protein kinases (CDPKs). *Molecular Plant, 6,* 1274-1289. https://doi.org/10.1093/mp/sss158

Laurie, S., Feeney, K. A., Maathuis, F. J. M., Heard, P. J., Brown, S. J., & Leigh, R. A. (2002). A role for HKT1 in sodium uptake by wheat roots. *Plant Journal, 32,* 139-149. https://doi.org/10.1046/j.1365-313X.2002.01410.x

Lazof, D., & Laüchli, A. (1991). The nutritional status of the apical meristem of *Lactuca sativa* as affected by NaCl salinization - an electron-probe microanalytic study. *Planta*, *184*, 334-342. https://doi.org/10.1007/BF00195334

Leng, Q., Mercier, R. W., Hua, B. G., Fromm, H., & Berkowitz, G. A. (2002). Electrophysiological analysis of cloned cyclic nucleotide-gated ion channels. *Plant Physiology*, *128*, 400-410. https://doi.org/10.1104/pp.128.2.400

Li, H. T., Liu, H., Gao, X. S., & Zhang, H. X. (2009). Knock-out of Arabidopsis gene enhances tolerance to salt stress. *Biochemical and Biophysical Research Communications*, *382*, 637-641. https://doi.org/10.1016/j.bbrc.2009.03.091

Li, P. H., Zhang, G. Y., Gonzales, N., Guo, Y. Q., Hu, H. H., Park, S., & Zhao, J. (2016). Ca^{2+}-regulated and diurnal rhythm-regulated Na^{+}/Ca^{2+} exchanger AtNCL affects flowering time and auxin signalling in Arabidopsis. *Plant Cell and Environment*, *39*, 377-392. https://doi.org/10.1111/pce.12620

Liu, H., Tang, R. J., Zhang, Y., Wang, C. T., Lv, Q. D., Gao, X. S., Li, W. B., & Zhang, H. X. (2010). AtNHX3 is a vacuolar K^{+}/H^{+} antiporter required for low-potassium tolerance in. *Plant Cell and Environment*, *33*, 1989-1999. https://doi.org/10.1111/j.1365-3040.2010.02200.x

Liu, X. S., Feng, S. J., Wang, M. Q., Zhao, Y. N., Cao, H. W., Rono, J. K., & Yang, Z. M. (2020). OsNHAD is a chloroplast membrane-located transporter required for resistance to salt stress in rice (*Oryza sativa*). *Plant Science*, *291*, Article 110359. https://doi.org/10.1016/j.plantsci.2019.110359

Liu, G. Y., Yu, X., Zeng, Y. L., Li, B. Y., Wang, R., Wang, X. F., Zhao, X. Y., Jiang, L. W., & Guo, Y. (2025a). SOS2-FREE1 regulates SOS1 tonoplast sorting to promote Na^{+} compartmentalization in vacuole during salt stress response. *Journal of Integrative Plant Biology. 67,* 2545-2560. https://doi.org/10.1111/jipb.13970

Liu, X. X., Zhang, L., Zhao, Z. Q., Zheng, Y., Ren, Y., Zhao, X. M., Zhang, S. Z., Yang, G. D., Huang, J. G., Yan, K., Li, C. L., Zheng, C. C., & Wu, C. G. (2025b). Regulation of the non-selective Na^{+} plus importer capacity of NRT1.2/NPF4.6/AIT1 by SOS2-mediated phosphorylation in Arabidopsis. *Cell Reports*, *44*. https://doi.org/10.1016/j.celrep.2025.115729

Malagoli, P., Britto, D. T., Schulze, L. M., & Kronzucker, H. J. (2008). Futile Na^{+} cycling at the root plasma membrane in rice (*Oryza sativa* L.): kinetics, energetics, and relationship to salinity tolerance. *Journal of Experimental Botany, 59*, 4109-4117. https://doi.org/10.1093/jxb/ern249

Martínez-Atienza, J., Jiang, X. Y., Garciadeblas, B., Mendoza, I., Zhu, J. K., Pardo, J. M., & Quintero, F. J. (2007). Conservation of the salt overly sensitive pathway in rice. *Plant Physiology, 143,* 1001-1012. https://doi.org/10.1104/pp.106.092635

Mian, A., Oomen, R., Isayenkov, S., Sentenac, H., Maathuis, F. J. M., & Véry, A. A. (2011). Over-expression of an Na^{+}- and K^{+}-permeable HKT transporter in barley improves salt tolerance. *Plant Journal, 68,* 468-479. https://doi.org/10.1111/j.1365-313X.2011.04701.x

Morgan, S. H., Kader, M. A., & Lindberg, S. (2022). Cytosolic sodium influx in mesophyll protoplasts of *Arabidopsis thaliana*, wt, *sos1:1* and *nhx1* Differs and induces different calcium changes. *Plants-Basel*, *11*. https://doi.org/10.3390/plants11243439

Morita, S., Tamba, N., Shibasaka, M., Sasano, S., Kadoike, T., Urase, Y., Maruyama, M., Fukuoka, A., Yanai, J., Masumura, T., Ogihara, Y., Satoh, S., Tanaka, K., Katsuhara, M.,

& Nakayama, H. (2023). *In planta* evidence that the HAK transporter OsHAK2 is involved in Na^+ transport in rice. *Bioscience Biotechnology and Biochemistry, 87,* 482-490. https://doi.org/10.1093/bbb/zbad020

Müller, M., Kunz, H. H., Schroeder, J. I., Kemp, G., Young, H. S., & Neuhaus, H. E. (2014). Decreased capacity for sodium export out of Arabidopsis chloroplasts impairs salt tolerance, photosynthesis and plant performance. *Plant Journal*, *78*(4), 646-658. https://doi.org/10.1111/tpj.12501

Munns, R., James, R. A., Xu, B., Athman, A., Conn, S. J., Jordans, C., Byrt, C. S., Hare, R. A., Tyerman, S. D., Tester, M., Plett, D., & Gilliham, M. (2012). Wheat grain yield on saline soils is improved by an ancestral Na^+ transporter gene. *Nature Biotechnology, 30,* 360-364. https://doi.org/10.1038/nbt.2120

Munns, R., & Rawson, H. M. (1999). Effect of salinity on salt accumulation and reproductive development in the apical meristem of wheat and barley. *Australian Journal of Plant Physiology*, *26*, 459-464. https://doi.org/10.1071/Pp99049

Oi, T., Clode, P. L., Taniguchi, M., Colmer, T. D., & Kotula, L. (2022). Salt tolerance in relation to elemental concentrations in leaf cell vacuoles and chloroplasts of a C_4 monocotyledonous halophyte. *Plant Cell and Environment*, *45*, 1490-1506. https://doi.org/10.1111/pce.14279

Okada, T., Yamane, S., Yamaguchi, M., Kato, K., Shinmyo, A., Tsunemitsu, Y., Iwasaki, K., Ueno, D., & Demura, T. (2018). Characterization of rice KT/HAK/KUP potassium transporters and K^+ uptake by HAK1 from *Oryza sativa*. *Plant Biotechnology, 35,* 101-111. https://doi.org/10.5511/plantbiotechnology.18.0308a

Okamoto, M., Vidmar, J. J., & Glass, A. D. M. (2003). Regulation of NRT1 and NRT2 gene families of *Arabidopsis thaliana*: Responses to nitrate provision. *Plant and Cell Physiology*, *44*, 304-317. https://doi.org/10.1093/pcp/pcg036

Oomen, R., Benito, B., Sentenac, H., Rodríguez-Navarro, A., Talón, M., Véry, A. A., & Domingo, C. (2012). HKT2;2/1, a K^+-permeable transporter identified in a salt-tolerant rice cultivar through surveys of natural genetic polymorphism. *Plant Journal, 71,* 750-762. https://doi.org/10.1111/j.1365-313X.2012.05031.x

Peng, L. R., Xiao, H. J., Li, R., Zeng, Y., Gu, M., Moran, N., Yu, L., & Xu, G. H. (2023). Potassium transporter OsHAK18 mediates potassium and sodium circulation and sugar translocation in rice. *Plant Physiology, 193,* 2003-2020. https://doi.org/10.1093/plphys/kiad435

Qiu, Q. S., Barkla, B. J., Vera-Estrella, R., Zhu, J. K., & Schumaker, K. S. (2003). Na^+/H^+ exchange activity in the plasma membrane of Arabidopsis. *Plant Physiology*, *132*, 1041-1052. https://doi.org/10.1104/pp.102.010421

Qiu, Q. S., Guo, Y., Dietrich, M. A., Schumaker, K. S., & Zhu, J. K. (2002). Regulation of SOS1, a plasma membrane Na^+/H^+ exchanger in *Arabidopsis thaliana*, by SOS2 and SOS3. *Proceedings of the National Academy of Sciences of the United States of America*, *99*, 8436-8441. https://doi.org/10.1073/pnas.122224699

Qiu, J., McGaughey, S. A., Byrt, C. S., & Tyerman, S. D. (2025). Post-translational modification acts as a digital like switch influencing AtPIP2;1 water and cation permeability. *Scientific Reports*, *15*. https://doi.org/10.1038/s41598-025-06200-9

Quintero, F. J., Ohta, M., Shi, H. Z., Zhu, J. K., & Pardo, J. M. (2002). Reconstitution in yeast of the Arabidopsis SOS signaling pathway for Na^+ homeostasis. *Proceedings of the National Academy of Sciences of the United States of America*, *99*, 9061-9066. https://doi.org/10.1073/pnas.132092099

Ramakrishna, P., Gámez-Arjona, F. M., Bellani, E., Martin-Olmos, C., Escrig, S., De Bellis, D., De Luca, A., Pardo, J. M., Quintero, F. J., Genoud, C., Sánchez-Rodriguez, C., Geldner, N., & Meibom, A. (2025). Elemental cryo-imaging reveals SOS1-dependent vacuolar sodium accumulation. *Nature*, *637*. https://doi.org/10.1038/s41586-024-08403-y

Ranf, S., Wünnenberg, P., Lee, J., Becker, D., Dunkel, M., Hedrich, R., Scheel, D., & Dietrich, P. (2008). Loss of the vacuolar cation channel, AtTPC1, does not impair Ca^{2+} signals induced by abiotic and biotic stresses. *Plant Journal*, *53*, 287-299. https://doi.org/10.1111/j.1365-313X.2007.03342.x

Ren, Z. H., Gao, J. P., Li, L. G., Cai, X. L., Huang, W., Chao, D. Y., Zhu, M. Z., Wang, Z. Y., Luan, S., & Lin, H. X. (2005). A rice quantitative trait locus for salt tolerance encodes a sodium transporter. *Nature Genetics, 37,* 1141-1146. https://doi.org/10.1038/ng1643

Riedelsberger, J., Miller, J. K., Valdebenito-Maturana, B., Piñeros, M. A., Gonzalez, W., & Dreyer, I. (2021). Plant HKT channels: an updated view on structure, function and gene regulation. International *Journal of Molecular Sciences, 22,* Article 1892. https://doi.org/10.3390/ijms22041892

Robinson, S. P., & Downton, W. (1985). Potassium, sodium and chloride ion concentrations in leaves and isolated chloroplasts of the halophyte *Suaeda australis* R. Br. *Functional Plant Biology*, *12*, 471-479. https://doi.org/10.1071/PP9850471

Robinson, S. P., & Downton, W. J. S. (1984). Potassium, sodium, and chloride content of isolated intact chloroplasts in relation to ionic compartmentation in leaves. Archives of *Biochemistry and Biophysics, 228,* 197-206. https://doi.org/10.1016/0003-9861(84)90061-4

Robinson, S. P., Downton, W. J. S., & Millhouse, J. A. (1983). Photosynthesis and ion content of leaves and isolated-chloroplasts of salt-stressed spinach. *Plant Physiology*, *73*, 238-242. https://doi.org/10.1104/pp.73.2.238

Rosas-Santiago, P., Lagunas-Gómez, D., Barkla, B. J., Vera-Estrella, R., Lalonde, S., Jones, A., Frommer, W. B., Zimmermannova, O., Sychrová, H., & Pantoja, O. (2015). Identification of rice cornichon as a possible cargo receptor for the Golgi-localized sodium transporter OsHKT1;3. *Journal of Experimental Botany, 66,* 2733-2748. https://doi.org/10.1093/jxb/erv069

Roy, S. J., Gilliham, M., Berger, B., Essah, P. A., Cheffings, C., Miller, A. J., Davenport, R. J., Liu, L. H., Skynner, M. J., Davies, J. M., Richardson, P., Leigh, R. A., & Tester, M. (2008). Investigating glutamate receptor-like gene co-expression in *Arabidopsis thaliana*. *Plant Cell and Environment*, *31*, 861-871. https://doi.org/10.1111/j.1365-3040.2008.01801.x

Rubio, F., Gassmann, W., & Schroeder, J. I. (1995). Sodium-driven potassium uptake by the plant potassium transporter HKT1 and mutations conferring salt tolerance. *Science, 270,* 1660-1663. https://doi.org/10.1126/science.270.5242.1660

Rus, A., Yokoi, S., Sharkhuu, A., Reddy, M., Lee, B. H., Matsumoto, T. K., Koiwa, H., Zhu, J. K., Bressan, R. A., & Hasegawa, P. M. (2001). AtHKT1 is a salt tolerance determinant that controls Na^+ entry into plant roots. *Proceedings of the National Academy of Sciences of the United States of America*, *98*, 14150-14155. https://doi.org/10.1073/pnas.241501798

Saha, J., Chaudhuri, D., Kundu, A., Bhattacharya, S., Roy, S., & Giri, K. (2023). Phylogenetic, structural, functional characterisation and effect of exogenous spermidine on rice (*Oryza sativa*) HAK transporters under salt stress. *Functional Plant Biology, 50,* 160-182. https://doi.org/10.1071/fp22059

Santa-María, G. E., Rubio, F., Dubcovsky, J., & Rodríguez-Navarro, A. (1997). The HAK1 gene of barley is a member of a large gene family and encodes a high-affinity potassium transporter. *Plant Cell, 9,* 2281-2289. https://doi.org/10.1105/tpc.9.12.2281

Schachtman, D. P., Kumar, R., Schroeder, J. I., & Marsh, E. L. (1997). Molecular and functional characterization of a novel low-affinity cation transporter (LCT1) in higher plants. *Proceedings of the National Academy of Sciences of the United States of America, 94,* 11079-11084. https://doi.org/DOI 10.1073/pnas.94.20.11079

Schachtman, D. P., & Schroeder, J. I. (1994). Structure and transport mechanism of a high affinity potassium uptake transporter from higher plants. *Nature, 370,* 655-658. https://doi.org/10.1038/370655a0

Schönknecht, G., Spoormaker, P., Steinmeyer, R., Brüggeman, L., Ache, P., Dutta, R., Reintanz, B., Godde, M., Hedrich, R., & Palme, K. (2002). KCO1 is a component of the slow-vacuolar (SV) ion channel. *FEBS Letters, 511,* 28-32. https://doi.org/10.1016/S0014-5793(01)03273-2

Schroppel-Meier, G., & Kaiser, W. M. (1988). Ion homeostasis in chloroplasts under salinity and mineral deficiency. 1. Solute concentrations in leaves and chloroplasts from

spinach plants under NaCl or $NaNO_3$ salinity. *Plant Physiology*, *87*, 822-827. https://doi.org/10.1104/pp.87.4.822

Shen, L. K., Fan, W. X., Li, N., Wu, Q., Chen, D., Luan, J. X., Zhang, G. A., Tian, Q. X., Jing, W., Zhang, Q., & Zhang, W. H. (2023). Rice potassium transporter OsHAK18 mediates phloem K^+ loading and redistribution. *Plant Journal, 116,* 201-216. https://doi.org/10.1111/tpj.16371

Shi, H. Z., Ishitani, M., Kim, C. S., & Zhu, J. K. (2000). The *Arabidopsis thaliana* salt tolerance gene SOS1 encodes a putative Na^+/H^+ antiporter. *Proceedings of the National Academy of Sciences of the United States of America*, *97*, 6896-6901. https://doi.org/10.1073/pnas.120170197

Shi, H. Z., Lee, B. H., Wu, S. J., & Zhu, J. K. (2003). Overexpression of a plasma membrane Na^+/H^+ antiporter gene improves salt tolerance in *Arabidopsis thaliana*. *Nature Biotechnology*, *21*, 81-85. https://doi.org/10.1038/nbt766

Shi, H. Z., Quintero, F. J., Pardo, J. M., & Zhu, J. K. (2002). The putative plasma membrane Na^+/H^+ antiporter SOS1 controls long-distance Na^+ transport in plants. *Plant Cell*, *14*, 465-477. https://doi.org/10.1105/tpc.010371

Shibasaka, M., Sasano, S., Utsugi, S., & Katsuhara, M. (2012). Functional characterization of a novel plasma membrane intrinsic protein2 in barley. *Plant Signaling & Behavior, 7,* 1648-1652. https://doi.org/10.4161/psb.22294

Sunarpi, Horie, T., Motoda, J., Kubo, M., Yang, H., Yoda, K., Horie, R., Chan, W. Y., Leung, H. Y., Hattori, K., Konomi, M., Osumi, M., Yamagami, M., Schroeder, J. I., & Uozumi, N. (2005). Enhanced salt tolerance mediated by AtHKT1 transporter-induced Na^+ unloading from xylem vessels to xylem parenchyma cells. *Plant Journal*, *44*, 928-938. https://doi.org/10.1111/j.1365-313X.2005.02595.x

Suzuki, K., Yamaji, N., Costa, A., Okuma, E., Kobayashi, N. I., Kashiwagi, T., Katsuhara, M., Wang, C., Tanoi, K., Murata, Y., Schroeder, J. I., Ma, J. F., & Horie, T. (2016). OsHKT1;4-mediated Na^+ transport in stems contributes to Na^+ exclusion from leaf blades of rice at the reproductive growth stage upon salt stress. *BMC Plant Biology, 16,* 22. https://doi.org/10.1186/s12870-016-0709-4

Talke, I. N., Blaudez, D., Maathuis, F. J. M., & Sanders, D. (2003). CNGCs: prime targets of plant cyclic nucleotide signalling? *Trends in Plant Science*, *8*, 286-293. https://doi.org/10.1016/s1360-1385(03)00099-2

Tapken, D., & Hollmann, M. (2008). *Arabidopsis thaliana* glutamate receptor ion channel function demonstrated by ion pore transplantation. *Journal of Molecular Biology, 383,* 36-48. https://doi.org/10.1016/j.jmb.2008.06.076

Tian, Q. X., Tao, X. Y., Dong, M. Y., Yu, T. Y., Fang, Y. X., Xue, D. W., & Zhang, X. Q. (2024). Genome-wide identification and genetic characterization of HKT potassium

transporters and their response to abiotic stress in barley. *Plant Growth Regulation, 104,* 1589-1604. https://doi.org/10.1007/s10725-024-01244-9

Tounsi, S., Ben Amar, S., Masmoudi, K., Sentenac, H., Brini, F., & Véry, A. A. (2016). Characterization of Two HKT1; 4 Transporters from *Triticum monococcum* to elucidate the determinants of the wheat salt tolerance Nax1 QTL. *Plant and Cell Physiology, 57,* 2047-2057. https://doi.org/10.1093/pcp/pcw123

Tounsi, S., Saïdi, M. N., Abdelhedi, R., Feki, K., Bahloul, N., Alcon, C., Masmoudi, K., & Brini, F. (2021). Functional analysis of TmHKT1;4-A2 promoter through deletion analysis provides new insight into the regulatory mechanism underlying abiotic stress adaptation. *Planta, 253,* Article 18. https://doi.org/10.1007/s00425-020-03533-9

Tran, S. T. H., Horie, T., Imran, S., Qiu, J. E., McGaughey, S., Byrt, C. S., Tyerman, S. D., & Katsuhara, M. (2020). A Survey of barley PIP aquaporin ionic conductance reveals Ca^{2+}-sensitive Na^+ and K^+ conductance. *International Journal of Molecular Sciences, 21,* 7135. https://doi.org/10.3390/ijms21197135

Tran, S. T. H., Katsuhara, M., Mito, Y., Onishi, A., Higa, A., Ono, S., Paul, N. C., Horie, R., Harada, Y., & Horie, T. (2025). OsPIP2;4 aquaporin water channel primarily expressed in roots of rice mediates both water and nonselective Na^+ and K^+ conductance. *Scientific Reports, 15,* 12857, Article 12857. https://doi.org/10.1038/s41598-025-96259-1

Venema, K., Quintero, F. J., Pardo, J. M., & Donaire, J. P. (2002). The Arabidopsis Na^+/H^+ exchanger AtNHX1 catalyzes low affinity Na^+ and K^+ transport in reconstituted liposomes. *Journal of Biological Chemistry*, *277*, 2413-2418. https://doi.org/10.1074/jbc.M105043200

Wang, P., Li, Z. W., Wei, J. S., Zhao, Z. L., Sun, D. Y., & Cui, S. J. (2012). A Na^+/Ca^{2+} exchanger-like protein (AtNCL) involved in salt stress in Arabidopsis. *Journal of Biological Chemistry*, *287*, 44062-44070. https://doi.org/10.1074/jbc.M112.351643

Wang, Q., Guan, C., Wang, P., Lv, M. L., Ma, Q., Wu, G. Q., Bao, A. K., Zhang, J. L., & Wang, S. M. (2015). AtHKT1;1 and AtHAK5 mediate low-affinity Na^+ uptake in *Arabidopsis thaliana* under mild salt stress. *Plant Growth Regulation*, *75*, 615-623. https://doi.org/10.1007/s10725-014-9964-2

Wang, T. B., Gassmann, W., Rubio, F., Schroeder, J. I., & Glass, A. D. M. (1998). Rapid up-regulation of *HKT1*, a high-affinity potassium transporter gene, in roots of barley and wheat following withdrawal of potassium. *Plant Physiology, 118,* 651-659. https://doi.org/10.1104/pp.118.2.651

Xu, B., Hrmova, M., & Gilliham, M. (2020). High affinity Na^+ transport by wheat HKT1;5 is blocked by K^+. *Plant Direct, 4,* Article e00275. https://doi.org/10.1002/pld3.275

Xu, B., Waters, S., Byrt, C. S., Plett, D., Tyerman, S. D., Tester, M., Munns, R., Hrmova, M., & Gilliham, M. (2018). Structural variations in wheat HKT1;5 underpin differences in Na^+

transport capacity. *Cellular and Molecular Life Sciences, 75,* 1133-1144. https://doi.org/10.1007/s00018-017-2716-5

Yamaguchi, T., Aharon, G. S., Sottosanto, J. B., & Blumwald, E. (2005). Vacuolar Na^+/H^+ antiporter cation selectivity is regulated by calmodulin from within the vacuole in a Ca^{2+}- and pH-dependent manner. *Proceedings of the National Academy of Sciences of the United States of America*, *102*, 16107-16112. https://doi.org/10.1073/pnas.0504437102

Yamaguchi, T., Apse, M. P., Shi, H. Z., & Blumwald, E. (2003). Topological analysis of a plant vacuolar Na^+/H^+ antiporter reveals a luminal C terminus that regulates antiporter cation selectivity. *Proceedings of the National Academy of Sciences of the United States of America*, *100*, 12510-12515. https://doi.org/10.1073/pnas.2034966100

Yao, X., Horie, T., Xue, S. W., Leung, H. Y., Katsuhara, M., Brodsky, D. E., Wu, Y., & Schroeder, J. I. (2010). Differential sodium and potassium transport selectivities of the rice OsHKT2;1 and OsHKT2;2 transporters in plant cells. *Plant Physiology, 152,* 341-355. https://doi.org/10.1104/pp.109.145722

Yokoi, S., Quintero, F. J., Cubero, B., Ruiz, M. T., Bressan, R. A., Hasegawa, P. M., & Pardo, J. M. (2002). Differential expression and function of NHX Na^+/H^+ antiporters in the salt stress response. *Plant Journal*, *30*, 529-539. https://doi.org/10.1046/j.1365-313X.2002.01309.x

Zhang, H., Kim, M. S., Sun, Y., Dowd, S. E., Shi, H. Z., & Paré, P. W. (2008). Soil bacteria confer plant salt tolerance by tissue-specific regulation of the sodium transporter HKT1. *Molecular Plant-Microbe Interactions*, *21*, 737-744. https://doi.org/10.1094/mpmi-21-6-0737

Zhang, L. A., Sun, X. Y., Li, Y. F., Luo, X., Song, S. W., Chen, Y., Wang, X. H., Mao, D. D., Chen, L. B., & Luan, S. (2021). Rice Na^+-permeable transporter OsHAK12 mediates shoots Na^+ exclusion in response to salt stress. *Frontiers in Plant Science, 12.* https://doi.org/10.3389/fpls.2021.771746

Zhong, H. L., & Laüchli, A. (1994). Spatial distribution of solutes, K, Na, Ca and their deposition rates in the growth zone of primary cotton roots: Effects of NaCl and $CaCl_2$. *Planta*, *194*, 34-41. https://doi.org/10.1007/BF00201032

Zhu, J., Sun, C. Q., Zhang, Y. H., Zhang, M. N., Zhao, C. C., Lv, C., Guo, B. J., Wang, F. F., Zhou, M. X., & Xu, R. G. (2024). Functional analysis on the role of HvHKT1.4 in barley (*Hordeum vulgare* L.) salinity tolerance. *Plant Physiology and Biochemistry, 215,* Article 109061. https://doi.org/10.1016/j.plaphy.2024.109061
