# Supplementary material for: Cellular aspects of Na+ homeostasis in plants: Quantitative approaches
Source: Quant Plant Biol. 2026 Mar 2;7:e13. doi: 10.1017/qpb.2026.10040 (PMC13280860; doi:10.1017/qpb.2026.10040)
Supplement: Peer review history

# Reviewer report: Review: Cellular aspects of Na ^**+**^ homeostasis in plants: Quantitative approaches — R0/PR2

**DOI:** 10.1017/qpb.2026.10040.pr2

Tyerman et al provide a very interesting overview of the Na+ ion in plants. They clearly present the current knowledge (and the borders of knowledge) in this context. Interestingly, the authors also implemented a quantitative modeling approach and expanded the “homeostat concept” introduced by a Chilean group for K+ to sodium. Conclusions from the model pave the way for further theoretical analyses of the roles of the twins Na+ and K+ in plants. This manuscript is very well written and the provided Figures are of very high quality. Nevertheless, this reviewer encourages the authors for a further careful check of the text taking the following observations into account (this reviewer may have missed other minor points):

*) Tables 3, and S3: it is not clear why the word “Biology” appears at several positions. Either explain clearly or remove.

*) l.368: “mv” -> “mV”

*) l.369: “though” -> “through”

*) l.469 “Fig. S2” -> Fig. S2 refers to Figure 5D. Line 469 still refers to Figure 4. Please check.

*) l.473-475 “). The dashed diagonal line indicates the approximate conductances required to achieve a (homeostatic) internal concentration of 10 mM Na+ at different [Na+]ext.” -> It is not clear what is meant. Please check.

*) Table S2 -> 3 numbers are highlighted in yellow and a reference in green. Why?

*) References to Tables S1, S2, S3 -> there are unintentional line breaks in some references: (1) Carden et al., (2) Halperin et al., (3) Kader et al., (4) Morgan et al., (5) Morita et al., (6) Oi et al., (7) Shen et al., (8) Zhang et al.

*) Figure S1 -> Would it be useful to present also the mathematical function that describes this curve?

*) Figure S4 -> Would it be useful to present also the mathematical function that describes this curve?

# Reviewer report: Review: Cellular aspects of Na ^**+**^ homeostasis in plants: Quantitative approaches — R0/PR3

**DOI:** 10.1017/qpb.2026.10040.pr3

Dear Editor and Authors

The manuscript of Tyerman et al. appears to me to be separated in two parts. In section 1 and 2, sodium homeostasis is explained in a classical way, by discussing the current state of knowledge about concentrations of sodium in different cell compartments and properties of ion transport proteins that have been related with translocation of sodium. In addition to providing information about sodium concentrations in the apoplast and cytosol, section 2 also discusses sodium transport in mitochondria and chloroplasts. This part of the paper is written in a comprehensive way and certainly useful for readers that would like to get insight information about sodium transport systems in plants. It would have been straight forward to discuss sodium transport at the plasma membrane and tonoplast in a similar way.

However, the conception of the manuscript changes dramatically with section 3. From here on, the idea of a “ion homeostat”, as described by Dreyer (2021) and Dreyer et al. (2024), is applied to sodium transport at the plasma membrane (section 3) and the tonoplast (section 4). The conclusions in section 5 only refer to these “Na+ homeostat” sections and not to section 2. I must admit that I can only follow part of the information that is provided in section 3 and 4 and it is likely that this modelling approach will only be appreciated by few specialists.

Because of the difference in concept between the two parts of the manuscript, my advice is to separate both. The first part can be complemented with a discussion on ion transport proteins on the plasma membrane and tonoplast and serve as a classical review. Part two is a modelling approach of sodium transport at the plasma membrane and tonoplast, using the ion homeostat hypothesis. As already explained above, I had a hard time to understand these sections and I think the modelling approach and graphs need a more detailed description, in order to convince a large audience.

Below, I list a number of other points of concern, which may help the authors to improve both parts of the manuscript.

Minor points of concern to part 1:

1. The following mistakes were found in the abbreviations:

GORK = Guard cell outward rectifier

CNGC = Cyclic nucleotide gated

NHX = Sodium/proton exchanger

KUP = Potassium uptake protein

2. Line 105 and at other positions, NaCl or Na+ is written without the addition of “concentration”.

3. Line 120, please explain the properties of HKT1,5-D in particular, and HKT-transporters in general.

4. Line 124, what is meant with “no homeostasis”?

5. Line 144, add reference for data obtained with maize.

6. Line 157, it is stated that [Na+]cyt data are lacking, but later on values are presented.

7. Line 166, what are “highly cytoplasmic” cells?

8. Table 1, please point out what the units are for the numbers in this table (probably mM).

9. Line 179, the statement that “homeostasis, control” is not perfect does not fit with the definition given on lines 90-93. Based on this definition homeostasis, it is not expected that cell keep the value of the respective ion at one and the same level. Instead homeostasis, will ensures that the ion concertation is kept with limits.

10. Section 2.4 and 2.5. I wonder why plants can grow without Na+, if this ion has an essential function in mitochondria and/or chloroplasts. Do plants have a backup mechanism for the lack of Na+?

11. Line 233, this line doesn’t make sense. Does Na+ eject itself?

12. Line 234, what is meant with a strong negative phenotype?

13. Line 243, what is meant with the light phase of photosynthesis?

14. Line 265, what does “a convincing contribution” mean?

15. Lines 279-280, please explain more clearly what caused higher whole leaf Na+ concentrations in knockdown lines.

16. Line 284, Here probably the “chloroplast stroma” is meant.

General points of concern related to part 2.

1. The paper of Berthomieu et al. (2003) shows that HKT1 is mainly expressed in the phloem cells of Arabidopsis, which is not in line with a role in a general homeostat that is active in all kinds of leaf cells. Is there a reason to assume that HKT1 has a different expression pattern in other plant species?

2. It remains unclear to the reader how the models shown in Figs 3, 4 and 5 will respond to changes in the extracellular Na+ concentration. In these models, the ensemble of ion transporters in the plasma membrane should act as a Na+ homeostat. So, one would like to know, how well this system can keep the cytosolic Na+ concentration at a steady level and what is the mechanism is behind this.

3. In support of figure 5, Li et al. (2017) found that the NPF7.3 transporter may function as a H+/K+ antiporter in Arabidopsis (Plant Cell 29: 2016–2026).

4. Just as for the other models, I could not understand, why the transporters at the tonoplast, shown in Fig. 6A, function as a Na+ homeostat. I think this need a better explanation.

# Editor report: Recommendation: Cellular aspects of Na ^**+**^ homeostasis in plants: Quantitative approaches — R0/PR4

**DOI:** 10.1017/qpb.2026.10040.pr4

**Affiliations:** CBSM - Faculty of Engineering, Universidad de Talca, Chile

Dears Stephen, Tracey, Jay and Rana,

Your manuscript “Cellular aspects of Na+ homeostasis in plants: Quantitative approaches” has been seen by two reviewers. While reviewer 1 (with modeling experience) appreciated the entire manuscript, reviewer 2 (apparently with little modeling experience) only appreciated the first part (until the model). From my personal point of view, the modeling approach is valuable, although I have to admit that it is tough to understand for unexperienced readers. To guarantee that the MS is appreciated by a larger readership, I invite you to tackle the issues raised by the reviewers in a major revision. You may have two options: (a) follow the advice of reviewer 2 and let the modeling part aside for the moment; (b) reduce the modeling part in the main text to a minimum (conclusions that can be drawn from modeling) and present the details in the supplementary material.

The statements of reviewer 2 coincide with my own experience that modeling needs to be explained in tiny steps to make it accessible to readers outside the modeling field. Thank you for your valuable contribution to the Research Topic “Quantitative approaches to cellular aspects of plant ion homeostasis”.

Best regards, Ingo

# Editor report: Decision: Cellular aspects of Na ^**+**^ homeostasis in plants: Quantitative approaches — R0/PR5

**DOI:** 10.1017/qpb.2026.10040.pr5

**Affiliations:** RDP, ENS de Lyon, France

No accompanying comment.

# Reviewer report: Review: Cellular aspects of Na ^**+**^ homeostasis in plants: Quantitative approaches — R1/PR7

**DOI:** 10.1017/qpb.2026.10040.pr7

In their revised version, Tyerman et al have extensively addressed the concerns of the reviewers. The following minor points were noted:

*) Figure 1: The color of point (12) in the legend (black text on light brown background) is different from the Figure (white text on green background). The authors may like to correct this.

*) l.117: please check the unit. “the cytosol is just a thin layer of 1-2 μM around the organelles.” -> should be “1-2 μm”.

*) Figure 2, legend: “The homeostat shown in (A) has limits for a range of g values such that ENa cannot be less than Vm or greater than the equilibrium potential for H+”. -> not entirely clear. Probably, it is meant “The homeostat shown in (A) has limits irrespective of the activities of the transporters (g values) such that ENa cannot be less than Vm or greater than the equilibrium potential for H+”.

*) Figure 2, legend, l.319: “depends on the values of g” -> To help the reader, it might be better to explain also here what g values are: “depends on the activities of the transporters (values of g).”

*) Figure 2, panel C: the value “0” at the right axis should read “0.1”

*) Supplementary Material: There are yellow frames behind Figures S4 and S7, which the authors may want to remove.

# Editor report: Recommendation: Cellular aspects of Na ^**+**^ homeostasis in plants: Quantitative approaches — R1/PR8

**DOI:** 10.1017/qpb.2026.10040.pr8

**Affiliations:** CBSM - Faculty of Engineering, Universidad de Talca, Chile

Dears Stephen, Tracey, Jay and Rana,

Your manuscript “Cellular aspects of Na+ homeostasis in plants: Quantitative approaches” has been peer-reviewed again. There are still a few minor points that need to be clarified.

Thank you again for your valuable contribution to the Research Topic “Quantitative approaches to cellular aspects of plant ion homeostasis”.

Best regards, Ingo

# Editor report: Decision: Cellular aspects of Na ^**+**^ homeostasis in plants: Quantitative approaches — R1/PR9

**DOI:** 10.1017/qpb.2026.10040.pr9

**Affiliations:** RDP, ENS de Lyon, France

No accompanying comment.

# Editor report: Recommendation: Cellular aspects of Na ^**+**^ homeostasis in plants: Quantitative approaches — R2/PR11

**DOI:** 10.1017/qpb.2026.10040.pr11

**Affiliations:** CBSM - Faculty of Engineering, Universidad de Talca, Chile

Dears Stephen, Tracey, Jay and Rana,

thanks for the final changes. There are no further issues. Thank you again for your valuable contribution to the Research Topic “Quantitative approaches to cellular aspects of plant ion homeostasis”.

Best regards, Ingo

# Editor report: Decision: Cellular aspects of Na ^**+**^ homeostasis in plants: Quantitative approaches — R2/PR12

**DOI:** 10.1017/qpb.2026.10040.pr12

**Affiliations:** RDP, ENS de Lyon, France

No accompanying comment.
